# The mutational constraint spectrum quantified from variation in 141,456 humans

**DOI:** 10.1038/s41586-020-2308-7

**Published:** 2020-05-27

**Authors:** Konrad J. Karczewski, Laurent C. Francioli, Grace Tiao, Beryl B. Cummings, Jessica Alföldi, Qingbo Wang, Ryan L. Collins, Kristen M. Laricchia, Andrea Ganna, Daniel P. Birnbaum, Laura D. Gauthier, Harrison Brand, Matthew Solomonson, Nicholas A. Watts, Daniel Rhodes, Moriel Singer-Berk, Eleina M. England, Eleanor G. Seaby, Jack A. Kosmicki, Raymond K. Walters, Katherine Tashman, Yossi Farjoun, Eric Banks, Timothy Poterba, Arcturus Wang, Cotton Seed, Nicola Whiffin, Jessica X. Chong, Kaitlin E. Samocha, Emma Pierce-Hoffman, Zachary Zappala, Anne H. O’Donnell-Luria, Eric Vallabh Minikel, Ben Weisburd, Monkol Lek, James S. Ware, Christopher Vittal, Irina M. Armean, Louis Bergelson, Kristian Cibulskis, Kristen M. Connolly, Miguel Covarrubias, Stacey Donnelly, Steven Ferriera, Stacey Gabriel, Jeff Gentry, Namrata Gupta, Thibault Jeandet, Diane Kaplan, Christopher Llanwarne, Ruchi Munshi, Sam Novod, Nikelle Petrillo, David Roazen, Valentin Ruano-Rubio, Andrea Saltzman, Molly Schleicher, Jose Soto, Kathleen Tibbetts, Charlotte Tolonen, Gordon Wade, Michael E. Talkowski, Genome Aggregation Database Consortium, Carlos A. Aguilar Salinas, Tariq Ahmad, Christine M. Albert, Diego Ardissino, Gil Atzmon, John Barnard, Laurent Beaugerie, Emelia J. Benjamin, Michael Boehnke, Lori L. Bonnycastle, Erwin P. Bottinger, Donald W. Bowden, Matthew J. Bown, John C. Chambers, Juliana C. Chan, Daniel Chasman, Judy Cho, Mina K. Chung, Bruce Cohen, Adolfo Correa, Dana Dabelea, Mark J. Daly, Dawood Darbar, Ravindranath Duggirala, Josée Dupuis, Patrick T. Ellinor, Roberto Elosua, Jeanette Erdmann, Tõnu Esko, Martti Färkkilä, Jose Florez, Andre Franke, Gad Getz, Benjamin Glaser, Stephen J. Glatt, David Goldstein, Clicerio Gonzalez, Leif Groop, Christopher Haiman, Craig Hanis, Matthew Harms, Mikko Hiltunen, Matti M. Holi, Christina M. Hultman, Mikko Kallela, Jaakko Kaprio, Sekar Kathiresan, Bong-Jo Kim, Young Jin Kim, George Kirov, Jaspal Kooner, Seppo Koskinen, Harlan M. Krumholz, Subra Kugathasan, Soo Heon Kwak, Markku Laakso, Terho Lehtimäki, Ruth J. F. Loos, Steven A. Lubitz, Ronald C. W. Ma, Daniel G. MacArthur, Jaume Marrugat, Kari M. Mattila, Steven McCarroll, Mark I. McCarthy, Dermot McGovern, Ruth McPherson, James B. Meigs, Olle Melander, Andres Metspalu, Benjamin M. Neale, Peter M. Nilsson, Michael C. O’Donovan, Dost Ongur, Lorena Orozco, Michael J. Owen, Colin N. A. Palmer, Aarno Palotie, Kyong Soo Park, Carlos Pato, Ann E. Pulver, Nazneen Rahman, Anne M. Remes, John D. Rioux, Samuli Ripatti, Dan M. Roden, Danish Saleheen, Veikko Salomaa, Nilesh J. Samani, Jeremiah Scharf, Heribert Schunkert, Moore B. Shoemaker, Pamela Sklar, Hilkka Soininen, Harry Sokol, Tim Spector, Patrick F. Sullivan, Jaana Suvisaari, E. Shyong Tai, Yik Ying Teo, Tuomi Tiinamaija, Ming Tsuang, Dan Turner, Teresa Tusie-Luna, Erkki Vartiainen, Marquis P. Vawter, James S. Ware, Hugh Watkins, Rinse K. Weersma, Maija Wessman, James G. Wilson, Ramnik J. Xavier, Benjamin M. Neale, Mark J. Daly, Daniel G. MacArthur

**Affiliations:** 1 Program in Medical and Population Genetics, Broad Institute of MIT and Harvard, Cambridge, MA USA; 2 Analytic and Translational Genetics Unit, Massachusetts General Hospital, Boston, MA USA; 3 Program in Biological and Biomedical Sciences, Harvard Medical School, Boston, MA USA; 4 Program in Bioinformatics and Integrative Genomics, Harvard Medical School, Boston, MA USA; 5 Center for Genomic Medicine, Massachusetts General Hospital, Boston, MA USA; 6 Institute for Molecular Medicine Finland, Helsinki, Finland; 7 Data Sciences Platform, Broad Institute of MIT and Harvard, Cambridge, MA USA; 8 Centre for Translational Bioinformatics, William Harvey Research Institute, Barts and the London School of Medicine and Dentistry, Queen Mary University of London and Barts Health NHS Trust, London, UK; 9 Stanley Center for Psychiatric Research, Broad Institute of MIT and Harvard, Cambridge, MA USA; 10 National Heart & Lung Institute and MRC London Institute of Medical Sciences, Imperial College London, London, UK; 11 Cardiovascular Research Centre, Royal Brompton & Harefield Hospitals NHS Trust, London, UK; 12 Department of Pediatrics, University of Washington, Seattle, WA USA; 13 Wellcome Sanger Institute, Wellcome Genome Campus, Hinxton, Cambridge UK; 14 Vertex Pharmaceuticals Inc, Boston, MA USA; 15 Division of Genetics and Genomics, Boston Children’s Hospital, Boston, MA USA; 16 Department of Pediatrics, Harvard Medical School, Boston, MA USA; 17 Department of Genetics, Yale School of Medicine, New Haven, CT USA; 18 Broad Genomics, Broad Institute of MIT and Harvard, Cambridge, MA USA; 19 Department of Neurology, Harvard Medical School, Boston, MA USA; 150 Present Address: Centre for Population Genomics, Garvan Institute of Medical Research, and UNSW Sydney, Sydney, New South Wales Australia; 151 Present Address: Centre for Population Genomics, Murdoch Children’s Research Institute, Melbourne, Victoria Australia; 20 Unidad de Investigacion de Enfermedades Metabolicas, Instituto Nacional de Ciencias Medicas y Nutricion, Mexico City, Mexico; 21 Peninsula College of Medicine and Dentistry, Exeter, UK; 22 Division of Preventive Medicine, Brigham and Women’s Hospital, Boston, MA USA; 23 Division of Cardiovascular Medicine, Brigham and Women’s Hospital and Harvard Medical School, Boston, MA USA; 24 Department of Cardiology, University Hospital, Parma, Italy; 25 Department of Biology, Faculty of Natural Sciences, University of Haifa, Haifa, Israel; 26 Department of Medicine, Albert Einstein College of Medicine, Bronx, NY USA; 27 Department of Genetics, Albert Einstein College of Medicine, Bronx, NY USA; 28 Department of Quantitative Health Sciences, Lerner Research Institute, Cleveland Clinic, Cleveland, OH USA; 29 Sorbonne Université, APHP, Gastroenterology Department, Saint Antoine Hospital, Paris, France; 30 Framingham Heart Study, National Heart, Lung, & Blood Institute and Boston University, Framingham, MA USA; 31 Department of Medicine, Boston University School of Medicine, Boston, MA USA; 32 Department of Epidemiology, Boston University School of Public Health, Boston, MA USA; 33 Department of Biostatistics, Center for Statistical Genetics, University of Michigan, Ann Arbor, MI USA; 34 National Human Genome Research Institute, National Institutes of Health, Bethesda, MD USA; 35 The Charles Bronfman Institute for Personalized Medicine, Icahn School of Medicine at Mount Sinai, New York, NY USA; 36 Department of Biochemistry, Wake Forest School of Medicine, Winston-Salem, NC USA; 37 Center for Genomics and Personalized Medicine Research, Wake Forest School of Medicine, Winston-Salem, NC USA; 38 Center for Diabetes Research, Wake Forest School of Medicine, Winston-Salem, NC USA; 39 Department of Cardiovascular Sciences and NIHR Leicester Biomedical Research Centre, University of Leicester, Leicester, UK; 40 NIHR Leicester Biomedical Research Centre, Glenfield Hospital, Leicester, UK; 41 Department of Epidemiology and Biostatistics, Imperial College London, London, UK; 42 Department of Cardiology, Ealing Hospital NHS Trust, Southall, UK; 43 Imperial College Healthcare NHS Trust, Imperial College London, London, UK; 44 Department of Medicine and Therapeutics, The Chinese University of Hong Kong, Hong Kong, China; 45 Department of Medicine, Harvard Medical School, Boston, MA USA; 46 Program for Neuropsychiatric Research, McLean Hospital, Belmont, MA USA; 47 Department of Medicine, University of Mississippi Medical Center, Jackson, MI USA; 48 Department of Epidemiology, Colorado School of Public Health, Aurora, CO USA; 49 Department of Medicine and Pharmacology, University of Illinois at Chicago, Chicago, IL USA; 50 Department of Genetics, Texas Biomedical Research Institute, San Antonio, TX USA; 51 Department of Biostatistics, Boston University School of Public Health, Boston, MA USA; 52 Cardiac Arrhythmia Service and Cardiovascular Research Center, Massachusetts General Hospital, Boston, MA USA; 53 Cardiovascular Epidemiology and Genetics, Hospital del Mar Medical Research Institute (IMIM), Barcelona, Catalonia Spain; 54 Centro de Investigación Biomédica en Red Enfermedades Cardiovaculares (CIBERCV), Barcelona, Catalonia Spain; 55 Department of Medicine, Medical School, University of Vic-Central University of Catalonia, Vic, Catalonia Spain; 56 Institute for Cardiogenetics, University of Lübeck, Lübeck, Germany; 57 DZHK (German Research Centre for Cardiovascular Research), partner site Hamburg/Lübeck/Kiel, Lübeck, Germany; 58 University Heart Center Lübeck, Lübeck, Germany; 59 Estonian Genome Center, Institute of Genomics, University of Tartu, Tartu, Estonia; 60 Helsinki University and Helsinki University Hospital, Clinic of Gastroenterology, Helsinki, Finland; 61 Diabetes Unit, Massachusetts General Hospital, Boston, MA USA; 62 Center for Genomic Medicine, Massachusetts General Hospital, Boston, MA USA; 63 Program in Metabolism, Broad Institute of MIT and Harvard, Cambridge, MA USA; 64 Institute of Clinical Molecular Biology (IKMB), Christian-Albrechts-University of Kiel, Kiel, Germany; 65 Bioinformatics Consortium, Massachusetts General Hospital, Boston, MA USA; 66 Cancer Genome Computational Analysis Group, Broad Institute of MIT and Harvard, Cambridge, MA USA; 67 Department of Pathology, Massachusetts General Hospital, Boston, MA USA; 68 Cancer Center, Massachusetts General Hospital, Boston, MA USA; 69 Endocrinology and Metabolism Department, Hadassah-Hebrew University Medical Center, Jerusalem, Israel; 70 Department of Psychiatry and Behavioral Sciences, SUNY Upstate Medical University, Syracuse, NY USA; 71 Institute for Genomic Medicine, Columbia University Medical Center, Hammer Health Sciences, New York, NY USA; 72 Department of Genetics and Development, Columbia University Medical Center, Hammer Health Sciences, New York, NY USA; 73 Centro de Investigacion en Salud Poblacional, Instituto Nacional de Salud Publica, Cuernavaca, Mexico; 74 Genomics, Diabetes and Endocrinology, Lund University, Lund, Sweden; 75 Lund University Diabetes Centre, Malmö, Sweden; 76 Human Genetics Center, University of Texas Health Science Center at Houston, Houston, TX USA; 77 Department of Neurology, Columbia University, New York, NY USA; 78 Institute of Genomic Medicine, Columbia University, New York, NY USA; 79 Institute of Biomedicine, University of Eastern Finland, Kuopio, Finland; 80 Department of Psychiatry, Helsinki University Central Hospital, Lapinlahdentie, Helsinki, Finland; 81 Department of Medical Epidemiology and Biostatistics, Karolinska Institutet, Stockholm, Sweden; 82 Icahn School of Medicine at Mount Sinai, New York, NY USA; 83 Department of Neurology, Helsinki University Central Hospital, Helsinki, Finland; 84 Department of Public Health, Faculty of Medicine, University of Helsinki, Helsinki, Finland; 85 Cardiovascular Disease Initiative and Program in Medical and Population Genetics, Broad Institute of MIT and Harvard, Cambridge, MA USA; 86 Center for Genome Science, Korea National Institute of Health, Chungcheongbuk-do, South Korea; 87 MRC Centre for Neuropsychiatric Genetics & Genomics, Cardiff University School of Medicine, Cardiff, UK; 88 Department of Health, National Institute for Health and Welfare (THL), Helsinki, Finland; 89 Section of Cardiovascular Medicine, Department of Internal Medicine, Yale School of Medicine, New Haven, CT USA; 90 Division of Pediatric Gastroenterology, Emory University School of Medicine, Atlanta, GA USA; 91 Department of Internal Medicine, Seoul National University Hospital, Seoul, South Korea; 92 Institute of Clinical Medicine, The University of Eastern Finland, Kuopio, Finland; 93 Kuopio University Hospital, Kuopio, Finland; 94 Department of Clinical Chemistry, Fimlab Laboratories and Finnish Cardiovascular Research Center-Tampere, Faculty of Medicine and Health Technology, Tampere University, Tampere, Finland; 95 The Mindich Child Health and Development Institute, Icahn School of Medicine at Mount Sinai, New York, NY USA; 96 Li Ka Shing Institute of Health Sciences, The Chinese University of Hong Kong, Hong Kong, China; 97 Hong Kong Institute of Diabetes and Obesity, The Chinese University of Hong Kong, Hong Kong, China; 98 Cardiovascular Research REGICOR Group, Hospital del Mar Medical Research Institute (IMIM), Barcelona, Catalonia Spain; 99 Department of Genetics, Harvard Medical School, Boston, MA USA; 100 Oxford Centre for Diabetes, Endocrinology and Metabolism, University of Oxford, Churchill Hospital, Headington, Oxford UK; 101 Wellcome Centre for Human Genetics, University of Oxford, Oxford, UK; 102 Oxford NIHR Biomedical Research Centre, Oxford University Hospitals NHS Foundation Trust, John Radcliffe Hospital, Oxford, UK; 103 F Widjaja Foundation Inflammatory Bowel and Immunobiology Research Institute, Cedars-Sinai Medical Center, Los Angeles, CA USA; 104 Atherogenomics Laboratory, University of Ottawa Heart Institute, Ottawa, Canada; 105 Division of General Internal Medicine, Massachusetts General Hospital, Boston, MA USA; 106 Department of Clinical Sciences, University Hospital Malmo Clinical Research Center, Lund University, Malmo, Sweden; 107 Department of Clinical Sciences, Lund University, Skane University Hospital, Malmo, Sweden; 108 Instituto Nacional de Medicina Genómica (INMEGEN), Mexico City, Mexico; 109 Medical Research Institute, Ninewells Hospital and Medical School, University of Dundee, Dundee, UK; 110 Department of Molecular Medicine and Biopharmaceutical Sciences, Graduate School of Convergence Science and Technology, Seoul National University, Seoul, South Korea; 111 Department of Psychiatry, Keck School of Medicine at the University of Southern California, Los Angeles, CA USA; 112 Department of Psychiatry and Behavioral Sciences, Johns Hopkins University School of Medicine, Baltimore, MD USA; 113 Division of Genetics and Epidemiology, Institute of Cancer Research, London, UK; 114 Medical Research Center, Oulu University Hospital, Oulu, Finland and Research Unit of Clinical Neuroscience, Neurology, University of Oulu, Oulu, Finland; 115 Research Center, Montreal Heart Institute, Montreal, Quebec Canada; 116 Department of Medicine, Faculty of Medicine, Université de Montréal, Quebec, Canada; 117 Department of Biomedical Informatics, Vanderbilt University Medical Center, Nashville, TN USA; 118 Department of Medicine, Vanderbilt University Medical Center, Nashville, TN USA; 119 Department of Biostatistics and Epidemiology, Perelman School of Medicine at the University of Pennsylvania, Philadelphia, PA USA; 120 Department of Medicine, Perelman School of Medicine at the University of Pennsylvania, Philadelphia, PA USA; 121 Center for Non-Communicable Diseases, Karachi, Pakistan; 122 National Institute for Health and Welfare, Helsinki, Finland; 123 Deutsches Herzzentrum München, Munich, Germany; 124 Technische Universität München, Munich, Germany; 125 Division of Cardiovascular Medicine, Nashville VA Medical Center and Vanderbilt University, School of Medicine, Nashville, TN USA; 126 Department of Psychiatry, Icahn School of Medicine at Mount Sinai, New York, NY USA; 127 Department of Genetics and Genomic Sciences, Icahn School of Medicine at Mount Sinai, New York, NY USA; 128 Institute for Genomics and Multiscale Biology, Icahn School of Medicine at Mount Sinai, New York, NY USA; 129 Institute of Clinical Medicine, Neurology, University of Eastern Finlad, Kuopio, Finland; 130 Department of Twin Research and Genetic Epidemiology, King’s College London, London, UK; 131 Departments of Genetics and Psychiatry, University of North Carolina, Chapel Hill, NC USA; 132 Saw Swee Hock School of Public Health, National University of Singapore, National University Health System, Singapore, Singapore; 133 Department of Medicine, Yong Loo Lin School of Medicine, National University of Singapore, Singapore, Singapore; 134 Duke-NUS Graduate Medical School, Singapore, Singapore; 135 Life Sciences Institute, National University of Singapore, Singapore, Singapore; 136 Department of Statistics and Applied Probability, National University of Singapore, Singapore, Singapore; 137 Folkhälsan Institute of Genetics, Folkhälsan Research Center, Helsinki, Finland; 138 HUCH Abdominal Center, Helsinki University Hospital, Helsinki, Finland; 139 Center for Behavioral Genomics, Department of Psychiatry, University of California, San Diego, CA USA; 140 Institute of Genomic Medicine, University of California, San Diego, CA USA; 141 Juliet Keidan Institute of Pediatric Gastroenterology, Shaare Zedek Medical Center, The Hebrew University of Jerusalem, Jerusalem, Israel; 142 Instituto de Investigaciones Biomédicas UNAM, Mexico City, Mexico; 143 Instituto Nacional de Ciencias Médicas y Nutrición Salvador Zubirán, Mexico City, Mexico; 144 Radcliffe Department of Medicine, University of Oxford, Oxford, UK; 145 Department of Gastroenterology and Hepatology, University of Groningen and University Medical Center Groningen, Groningen, The Netherlands; 146 Department of Physiology and Biophysics, University of Mississippi Medical Center, Jackson, MS USA; 147 Program in Infectious Disease and Mi--crobiome, Broad Institute of MIT and Harvard, Cambridge, MA USA; 148 Center for Computational and Integrative Biology, Massachusetts General Hospital, Boston, MA USA; 149 Department of Psychiatry and Human Behavior, University of California Irvine, Irvine, CA USA

**Keywords:** Medical genomics, Rare variants

## Abstract

Genetic variants that inactivate protein-coding genes are a powerful source of information about the phenotypic consequences of gene disruption: genes that are crucial for the function of an organism will be depleted of such variants in natural populations, whereas non-essential genes will tolerate their accumulation. However, predicted loss-of-function variants are enriched for annotation errors, and tend to be found at extremely low frequencies, so their analysis requires careful variant annotation and very large sample sizes^1^. Here we describe the aggregation of 125,748 exomes and 15,708 genomes from human sequencing studies into the Genome Aggregation Database (gnomAD). We identify 443,769 high-confidence predicted loss-of-function variants in this cohort after filtering for artefacts caused by sequencing and annotation errors. Using an improved model of human mutation rates, we classify human protein-coding genes along a spectrum that represents tolerance to inactivation, validate this classification using data from model organisms and engineered human cells, and show that it can be used to improve the power of gene discovery for both common and rare diseases.

## Abstract (web summary)

A catalogue of predicted loss-of-function variants in 125,748 whole-exome and 15,708 whole-genome sequencing datasets from the Genome Aggregation Database (gnomAD) reveals the spectrum of mutational constraints that affect these human protein-coding genes.

## Main

The physiological function of most genes in the human genome remains unknown. In biology, as in many engineering and scientific fields, breaking the individual components of a complex system can provide valuable insight into the structure and behaviour of that system. For the discovery of gene function, a common approach is to introduce disruptive mutations into genes and determine their effects on cellular and physiological phenotypes in mutant organisms or cell lines^2^. Such studies have yielded valuable insight into eukaryotic physiology and have guided the design of therapeutic agents^3^. However, although studies in model organisms and human cell lines have been crucial in deciphering the function of many human genes, they remain imperfect proxies for human physiology.

Obvious ethical and technical constraints prevent the large-scale engineering of loss-of-function mutations in humans. However, recent exome and genome sequencing projects have revealed a surprisingly high burden of natural pLoF variation in the human population, including stop-gained, essential splice, and frameshift variants^1,4^, which can serve as natural models for inactivation of human genes. Such variants have already revealed much about human biology and disease mechanisms, through many decades of study of the genetic basis of severe Mendelian diseases^5^, most of which are driven by disruptive variants in either the heterozygous or homozygous state. These variants have also proved valuable in identifying potential therapeutic targets: confirmed LoF variants in the *PCSK9* gene have been causally linked to low levels of low-density lipoprotein cholesterol^6^, and have ultimately led to the development of several inhibitors of PCSK9 that are now in clinical use for the reduction of cardiovascular disease risk. A systematic catalogue of pLoF variants in humans and the classification of genes along a spectrum of tolerance to inactivation would provide a valuable resource for medical genetics, identifying candidate disease-causing mutations, potential therapeutic targets, and windows into the normal function of many currently uncharacterized human genes.

Several challenges arise when assessing LoF variants at scale. LoF variants are on average deleterious, and are thus typically maintained at very low frequencies in the human population. Systematic genome-wide discovery of these variants requires whole-exome or whole-genome sequencing of very large numbers of samples. In addition, LoF variants are enriched for false positives compared with synonymous or other benign variants, including mapping, genotyping (including somatic variation), and particularly, annotation errors^1^, and careful filtering is required to remove such artefacts.

Population surveys of coding variation enable the evaluation of the strength of natural selection at a gene or region level. As natural selection purges deleterious variants from human populations, methods to detect selection have modelled the reduction in variation (constraint)^7^ or shift in the allele frequency distribution^8^, compared to an expectation. For analyses of selection on coding variation, synonymous variation provides a convenient baseline, controlling for other potential population genetic forces that may influence the amount of variation as well as technical features of the local sequence. A model of constraint was previously applied to define a set of 3,230 genes with a high probability of intolerance to heterozygous pLoF variation (pLI)^4^ and estimated the selection coefficient for variants in these genes^9^. However, the ability to comprehensively characterize the degree of selection against pLoF variants is particularly limited, as for small genes, the expected number of mutations is still very low, even for samples of up to 60,000 individuals^4,10^. Furthermore, the previous dichotomization of pLI, although convenient for the characterization of a set of genes, disguises variability in the degree of selective pressure against a given class of variation and overlooks more subtle levels of intolerance to pLoF variation. With larger sample sizes, a more accurate quantitative measure of selective pressure is possible.

Here, we describe the detection of pLoF variants in a cohort of 125,748 individuals with whole-exome sequence data and 15,708 individuals with whole-genome sequence data, as part of the Genome Aggregation Database (gnomAD; https://gnomad.broadinstitute.org), the successor to the Exome Aggregation Consortium (ExAC). We develop a continuous measure of intolerance to pLoF variation, which places each gene on a spectrum of LoF intolerance. We validate this metric by comparing its distribution to several orthogonal indicators of constraint, including the incidence of structural variation and the essentiality of genes as measured using mouse gene knockout experiments and cellular inactivation assays. Finally, we demonstrate that this metric improves the interpretation of genetic variants that influence rare disease and provides insight into common disease biology. These analyses provide, to our knowledge, the most comprehensive catalogue so far of the sensitivity of human genes to disruption.

In a series of accompanying manuscripts, other complementary analyses of this dataset are described. Using an overlapping set of 14,237 whole genomes, the discovery and characterization of a wide variety of structural variants (large deletions, duplications, insertions, or other rearrangements of DNA) is reported^11^. The value of pLoF variants for the discovery and validation of therapeutic drug targets is explored^12^, and a case study of the use of these variants from gnomAD and other large reference datasets is provided to validate the safety of inhibition of LRRK2—a candidate therapeutic target for Parkinson’s disease^13^. By combining the gnomAD dataset with a large collection of RNA sequencing data from adult human tissues^14^, the value of tissue expression data in the interpretation of genetic variation across a range of human diseases is reported^15^. Finally, the effect of two understudied classes of human variation—multi-nucleotide variants^16^ and variants that create or disrupt open-reading frames in the 5′ untranslated region of human genes—is characterized and investigated^17^.

## A high-quality catalogue of variation

**Fig. 1:**
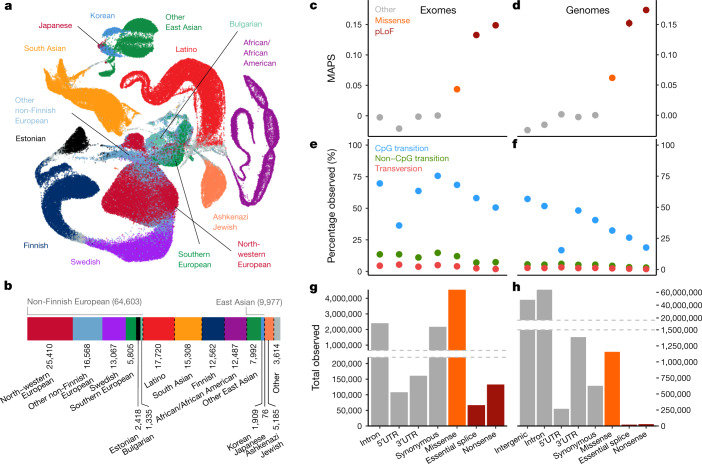
Aggregation of 141,456 exome and genome sequences. **a**, Uniform manifold approximation and projection (UMAP)^46,47^ plot depicting the ancestral diversity of all individuals in gnomAD, using seven principal components. Note that long-range distances in the UMAP space are not a proxy for genetic distance. **b**, The number of individuals by population and subpopulation in the gnomAD database. Colours representing populations in **a** and **b** are consistent. **c**, **d**, The mutability-adjusted proportion of singletons^4^ (MAPS) is shown across functional categories for SNVs in exomes (**c**; *x* axis shared with **e** and **g**) and genomes (**d**; *x* axis shared with **f** and **h**). Higher values indicate an enrichment of lower frequency variants, which suggests increased deleteriousness. **e**, **f**, The proportion of possible variants observed for each functional class for each mutational type for exomes (**e**) and genomes (**f**). CpG transitions are more saturated, except where selection (for example, pLoFs) or hypomethylation (5′ untranslated region) decreases the number of observations. **g**, **h**, The total number of variants observed in each functional class for exomes (**g**) and genomes (**h**). Error bars in **c**–**f** represent 95% confidence intervals (note that in some cases these are fully contained within the plotted point).

**Extended Data Fig. 1:**
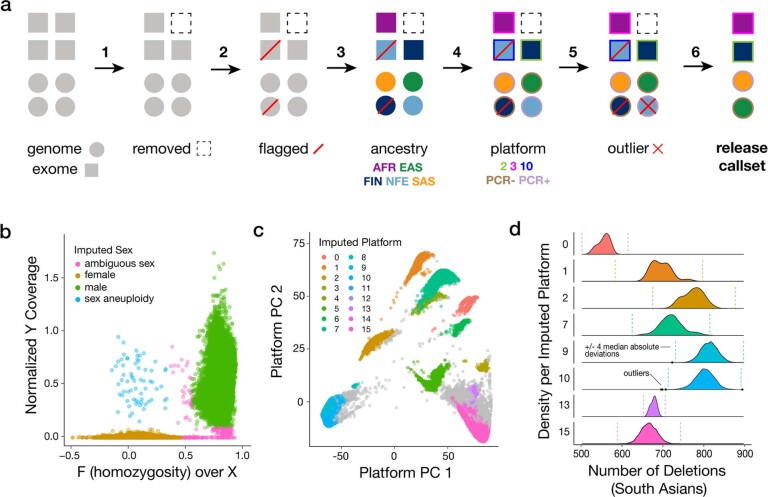
Overview of the sample quality control workflow. **a**, Exome (square) and genome (circle) samples underwent quality control in the following stages: hard filtering (step 1), relatedness inference (step 2), ancestry inference (step 3), platform inference (step 4, for exomes only), and population- and platform-specific outlier filtering (step 5). See Supplementary Information for further details. Except for samples failing hard filters (dotted outline), all quality control analyses were applied to all samples, regardless of the presence or absence of other quality control flags (such as relatedness, lack of release permissions, or outlier status; red diagonal bar). Assignment of ancestry labels is represented by fill colour and accompanying three-letter ancestry group abbreviation. Assignment of platform labels is represented by outline colour and a numbered label for exomes (corresponding to imputed platforms) and a PCR ± label for genomes. The final set of samples included in the gnomAD release (125,748 exomes and 15,708 genomes) was defined to be the set of unrelated samples with release permissions, no hard filter flags, and no population- and platform-specific outlier metrics (step 6). **b**, In exomes, the chromosomal sex of samples was inferred based on the inbreeding coefficient on chromosome X and the coverage of chromosome Y into male (green), female (amber), ambiguous sex (pink), and sex chromosome aneuploid (blue). **c**, The top two principal components from PCA-HDBSCAN analysis of exome capture regions. Sequencing platforms were inferred for exome samples based on principal component analysis of biallelic variant call rates over all known exome capture regions, and samples were assigned a cluster label (0–15, or unknown) using HDBSCAN. **d**, We performed platform- and population-specific outlier filtering for several quality-control metrics. The distribution of the number of deletions in samples from south Asian individuals across platforms is shown. Distributions (and accordingly, median and median absolute deviations) for these metrics varied widely both by population and sequencing platform (numbered on the *y* axis). Outliers (black dots) were defined as samples with values outside four median absolute deviations (shown by dotted vertical lines) from the median of a given metric.

We aggregated whole-exome sequencing data from 199,558 individuals and whole-genome sequencing data from 20,314 individuals. These data were obtained primarily from case–control studies of common adult-onset diseases, including cardiovascular disease, type 2 diabetes and psychiatric disorders. Each dataset, totalling more than 1.3 and 1.6 petabytes of raw sequencing data, respectively, was uniformly processed, joint variant calling was performed on each dataset using a standardized BWA-Picard-GATK pipeline^18^, and all data processing and analysis was performed using Hail^19^. We performed stringent sample quality control (Extended Data Fig. 1), removing samples with lower sequencing quality by a variety of metrics, samples from second-degree or closer related individuals across both data types, samples with inadequate consent for the release of aggregate data, and samples from individuals known to have a severe childhood-onset disease as well as their first-degree relatives. The final gnomAD release contains genetic variation from 125,748 exomes and 15,708 genomes from unique unrelated individuals with high-quality sequence data, spanning 6 global and 8 sub-continental ancestries (Fig. 1a, b), which we have made publicly available at https://gnomad.broadinstitute.org. We also provide subsets of the gnomAD datasets, which exclude individuals who are cases in case–control studies, or who are cases of a few particular disease types such as cancer and neurological disorders, or who are also aggregated in the Bravo TOPMed variant browser (https://bravo.sph.umich.edu).

**Extended Data Fig. 2:**
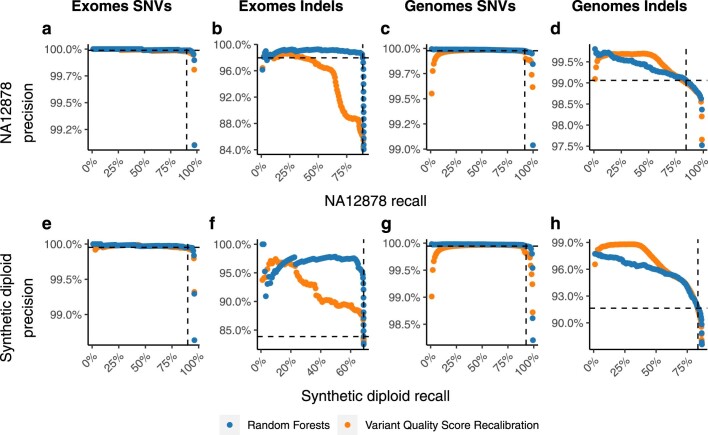
Variant calling performance for common variants. **a**–**h**, Precision-recall curves are shown for variant calls in two samples with independent gold-standard data, NA12878^49^ (**a**–**d**) and a synthetic diploid mixture^50^ (**e**–**h**). The random forest (blue) approach described here is compared to the current state-of-the-art GATK variant quality score recalibration (orange) for exome SNVs (**a**, **e**) and indels (**b**, **f**), and genome SNVs (**c**, **g**) and indels (**d**, **h**). Note that the indels presented in **f** and **h** exclude 1-base-pair (bp) indels as they are not well characterized in the synthetic diploid mixture gold standard sample. In all cases, at the thresholds chosen (dashed lines representing 10% and 20% of SNVs and indels filtered, respectively), random forest outperforms or is similar to variant quality score recalibration.

**Extended Data Fig. 3:**
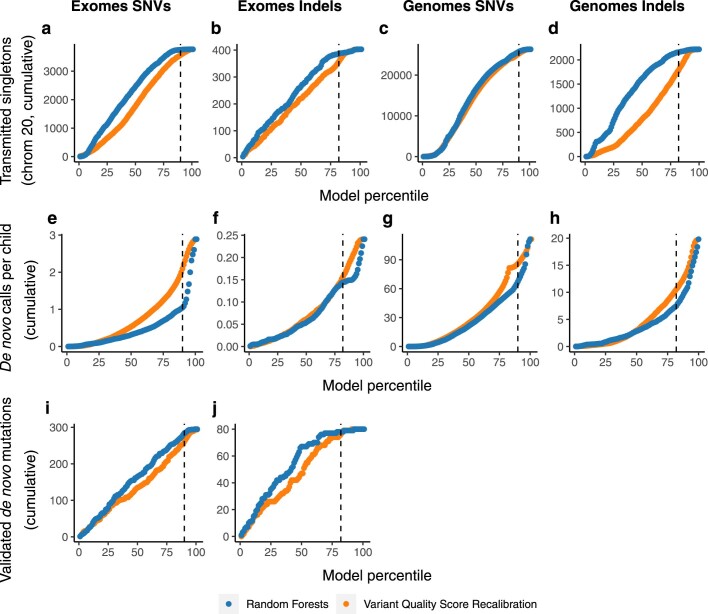
Variant calling performance for rare variants. **a**–**j**, The *x* axes show the cumulative ranked percentile for our random forest (blue) model and, as a comparison, for the current state-of-the-art GATK variant quality score recalibration (orange). That is, the point at 10 shows the performance of the 10% best-scored data; the point at 50 shows the performance 50% best-scored data. **a**–**d**, The number of transmitted singletons (singletons in the unrelated individuals that are transmitted to an offspring) on chromosome 20 for exome SNVs (**a**) and indels (**b**), and genome SNVs (**c**) and indels (**d**). Chromosome 20 was not used for training our random forest model. We expect most of these to be real variants because we observe Mendelian transmission of an allele that was sequenced independently in a parent and child. **e**–**h**, The number of bi-allelic de novo calls per child (4,568 exomes, 212 genomes) outside of low-complexity regions. The expectation is that there is approximately 1.6 de novo SNV (**e**) and 0.1 de novo indels per exome (**f**), and 65 de novo SNVs (**g**) and 5 de novo indels (**h**) per genome^20^. **i**, **j**, The number of independently validated de novo mutations, available for a subset of 331 exome samples for which de novo mutations were validated as part of other studies^51^. In all cases, at the thresholds chosen (dashed lines representing 10% and 20% of SNVs and indels filtered, respectively), random forest outperforms or is similar to variant quality score recalibration.

Among these individuals, we discovered 17.2 million and 261.9 million variants in the exome and genome datasets, respectively; these variants were filtered using a custom random forest process (Supplementary Information) to 14.9 million and 229.9 million high-quality variants. Comparing our variant calls in two samples for which we had independent gold-standard variant calls, we found that our filtering achieves very high precision (more than 99% for single nucleotide variants (SNVs), over 98.5% for indels in both exomes and genomes) and recall (over 90% for SNVs and more than 82% for indels for both exomes and genomes) at the single sample level (Extended Data Fig. 2). In addition, we leveraged data from 4,568 and 212 trios included in our exome and genome call-sets, respectively, to assess the quality of our rare variants. We found that our model retains over 97.8% of the transmitted singletons (singletons in the unrelated individuals that are transmitted to an offspring) on chromosome 20 (which was not used for model training) (Extended Data Fig. 3a–d). In addition, the number of putative de novo calls after filtering are in line with expectations^20^ (Extended Data Fig. 3e–h), and our model had a recall of 97.3% for de novo SNVs and 98% for de novo indels based on 375 independently validated de novo variants in our whole-exome trios (295 SNVs and 80 indels) (Extended Data Fig. 3i, j). Altogether, these results indicate that our filtering strategy produced a call-set with high precision and recall for both common and rare variants.

**Extended Data Fig. 4:**
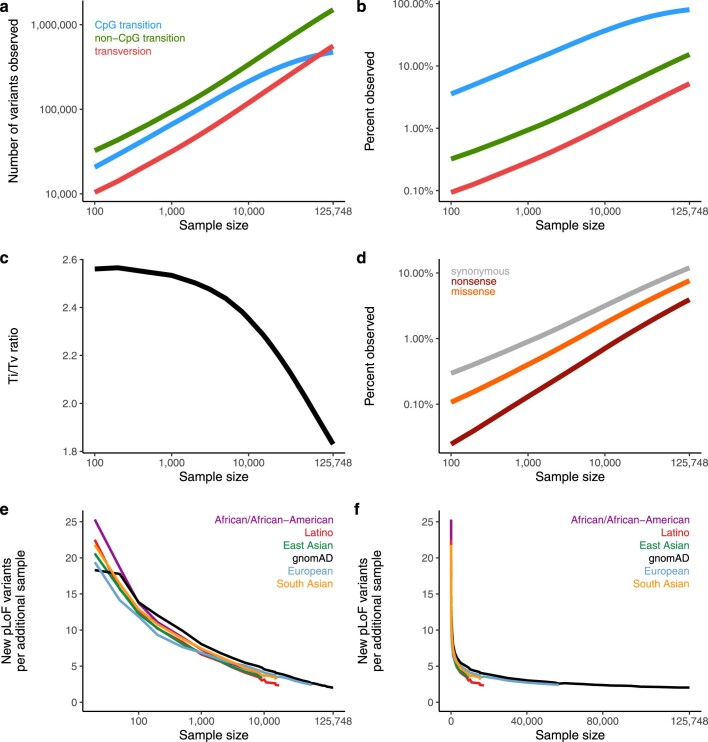
Variant discovery at large sample sizes. **a**, **b**, The total number of variants observed (**a**) and the proportion of possible variants observed (**b**) as a function of sample size, broken down by variant class. At large sample sizes, CpG transitions become saturated, as previously described^4^. Colours are consistent in **a** and **b. c**, This results in a decrease of the transition/transversion (Ti/Tv) ratio. **d**, When broken down by functional class, we observe the effects of selection, in which synonymous variants have the highest proportion observed, followed by missense and pLoF variants. **e**, **f**, The number of additional pLoF variants introduced into the cohort as a function of sample size on a log (**e**) and linear (**f**) scale. Here, gnomAD (black) refers to a uniform sampling from the population distribution of the full cohort of exome-sequenced individuals.

These variants reflect the expected patterns based on mutation and selection: we observe 84.9% of all possible consistently methylated CpG-to-TpG transitions that would create synonymous variants in the human exome (Supplementary Table 14), which indicates that at this sample size, we are beginning to approach mutational saturation of this highly mutable and weakly negatively selected variant class. However, we only observe 52% of methylated CpG stop-gained variants, which illustrates the action of natural selection removing a substantial fraction of gene-disrupting variants from the population (Fig. 1c–h). Across all mutational contexts, only 11.5% and 3.7% of the possible synonymous and stop-gained variants, respectively, are observed in the exome dataset, which indicates that current sample sizes remain far from capturing complete mutational saturation of the human exome (Extended Data Fig. 4).

## Identifying loss-of-function variants

**Fig. 2:**
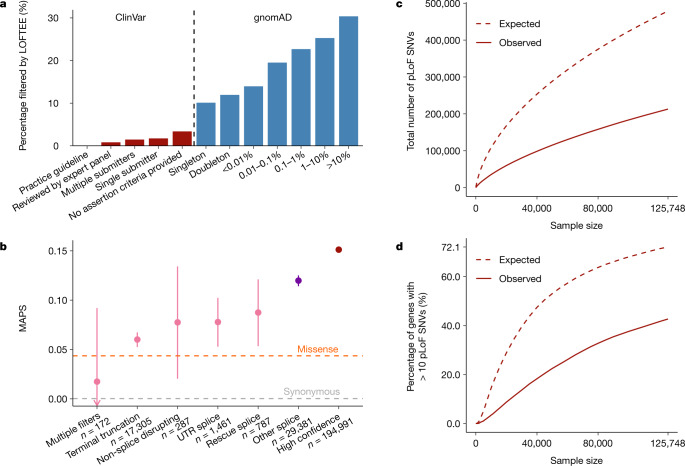
Generating a high-confidence set of pLoF variants. **a**, The percentage of variants filtered by LOFTEE grouped by ClinVar status and gnomAD frequency. Despite not using frequency information, LOFTEE removes a larger proportion of common variants, and a very low proportion of reported disease-causing variation. **b**, MAPS (see Fig. 1c, d) is shown by LOFTEE designation and filter. Variants filtered out by LOFTEE exhibit frequency spectra that are similar to those of missense variants; predicted splice variants outside the essential splice site are more rare, and high-confidence variants are very likely to be singletons. Only SNVs with at least 80% call rate are included here. Error bars represent 95% confidence intervals. **c**, **d**, The total number of pLoF variants (**c**), and proportion of genes with more than ten pLoF variants (**d**) observed and expected (in the absence of selection) as a function of sample size (downsampled from gnomAD). Selection reduces the number of variants observed, and variant discovery approximately follows a square-root relationship with the number of samples. At current sample sizes, we would expect to identify more than 10 pLoF variants for 72.1% of genes in the absence of selection.

**Extended Data Fig. 5:**
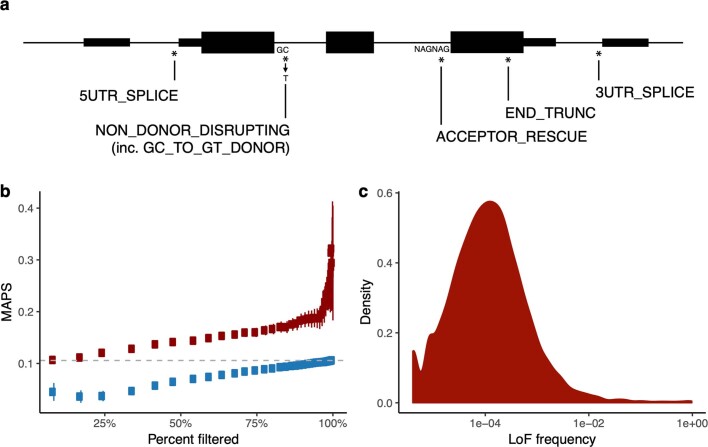
Using LOFTEE to create a high-confidence set of pLoF variation. **a**, Schematic of LOFTEE filters. LOFTEE filters out putative stop-gained, essential splice, and frameshift variants based on sequence and transcript context, as well as flagging exonic features such as conservation (not shown). For instance, variants that are not predicted to disrupt splicing based on retention of a strong splice site, or rescue of a nearby splice site. Additional filters not shown include: ANC_ALLELE (the alternative allele is the ancestral allele), NON_ACCEPTOR_DISRUPTING and DONOR_RESCUE (opposite to those already shown). **b**, To tune the END_TRUNC filter, we retained variants that pass the 50-bp rule (are more than 50 bp before the 3′-most splice site). The overall MAPS score for variants that fail this rule is shown in grey. For the remaining 39,072 variants, we computed the sum of the genomic evolutionary rate profiling (GERP) score of bases deleted by the variant. At 40 bins of this score, we compute the MAPS score for those variants retained at this threshold (red) compared to variants removed at this threshold (blue), and plot this as a function of the proportion of variants filtered at this threshold. We chose the 50% point as it retains variants with a MAPS score of 0.14, while removing variants with a MAPS score of 0.06. Error bars represent 95% confidence intervals. **c**, Density plot of aggregate pLoF frequency computed from high-confidence pLoF variants discovered using LOFTEE.

Some LoF variants will result in embryonic lethality in humans in a heterozygous state, whereas others are benign even at homozygosity, with a wide spectrum of effects in between. Throughout this manuscript, we define pLoF variants to be those that introduce a premature stop (stop-gained), shift-reported transcriptional frame (frameshift), or alter the two essential splice-site nucleotides immediately to the left and right of each exon (splice) found in protein-coding transcripts, and ascertain their presence in the cohort of 125,748 individuals with exome sequence data. As these variants are enriched for annotation artefacts^1^, we developed the loss-of-function transcript effect estimator (LOFTEE) package, which applies stringent filtering criteria from first principles (such as removing terminal truncation variants, as well as rescued splice variants, that are predicted to escape nonsense-mediated decay) to pLoF variants annotated by the variant effect predictor (Extended Data Fig. 5a). Despite not using frequency information, we find that this method disproportionately removes pLoF variants that are common in the population, which are known to be enriched for annotation errors^1^, while retaining rare, probable deleterious variations, as well as reported pathogenic variation (Fig. 2a). LOFTEE distinguishes high-confidence pLoF variants from annotation artefacts, and identifies a set of putative splice variants outside the essential splice site. The filtering strategy of LOFTEE is conservative in the interest of increasing specificity, filtering some potentially functional variants that display a frequency spectrum consistent with that of missense variation (Fig. 2b). Applying LOFTEE v1.0, we discover 443,769 high-confidence pLoF variants, of which 413,097 fall on the canonical transcripts of 16,694 genes. The number of pLoF variants per individual is consistent with previous reports^1^, and is highly dependent on the frequency filters chosen (Supplementary Table 17).

Aggregating across variants, we created a gene-level pLoF frequency metric to estimate the proportion of haplotypes that contain an inactive copy of each gene. We find that 1,555 genes have an aggregate pLoF frequency of at least 0.1% across all individuals in the dataset (Extended Data Fig. 5c), and 3,270 genes have an aggregate pLoF frequency of at least 0.1% in any one population. Furthermore, we characterized the landscape of genic tolerance to homozygous inactivation, identifying 4,332 pLoF variants that are homozygous in at least one individual. Given the rarity of true homozygous LoF variants, we expected substantial enrichment of such variants for sequencing and annotation errors, and we subjected this set to additional filtering and deep manual curation before defining a set of 1,815 genes (2,636 high-confidence variants) that are likely to be tolerant to biallelic inactivation (Supplementary Data 7).

## The LoF intolerance of human genes

**Extended Data Fig. 6:**
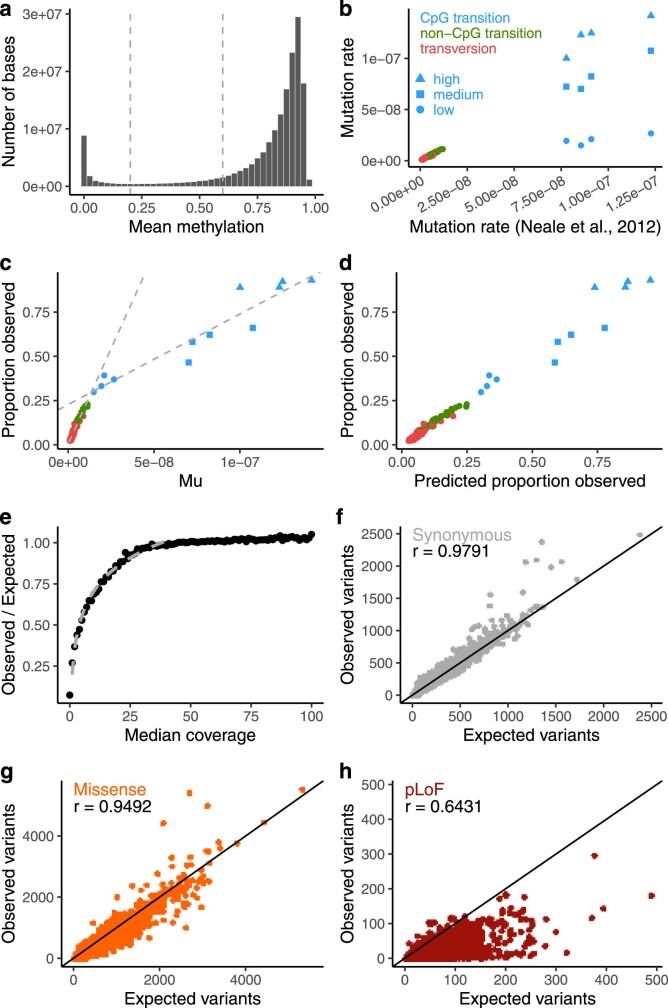
Computing the depletion of variation of functional categories. **a**, The distribution of mean methylation values across 37 tissues and across every CpG dinucleotide in the genome. We divided the genome into 3 levels (low methylation, missing or < 0.2; medium, 0.2–0.6; and high, >0.6) and computed all ensuing metrics based on these categories. **b**, Comparison of estimates of the mutation rate with previous estimates^52^. For transversions and non-CpG transitions, we observe a strong correlation (linear regression *r* = 0.98; *P* = 2.6 × 10^−65^). For CpG transitions, the new estimates are calculated separately for the three levels of methylation and track with these levels. Colours and shapes are consistent in **b**–**d**. **c**, For **c**–**e**, only synonymous variants are considered. The proportion of possible variants observed for each context is correlated with the mutation rate. We compute two fit lines, one for CpG transitions, and one for other contexts to calibrate our estimates. **d**, Calibration of each context to compute a predicted proportion observed after fitting the two models in **c**, which is used to calculate an expected number of variants at high coverage. **e**, With an expectation computed from high coverage regions, the observed/expected ratio follows a logarithmic trend with the median coverage below 40×, which is used to correct low coverage bases in the final expectation model. **f**–**h**, For each transcript, the observed number of variants is plotted against the expected number from the model described above, for synonymous (**f**), missense (**g**), and pLoF (**h**) variants, and the linear regression coefficient is shown. Note that the expectation does not include selection, and so, pLoF and, to a lesser extent, missense variants exhibit lower observed values than expected.

Just as a preponderance of pLoF variants is useful for identifying LoF-tolerant genes, we can conversely characterize the intolerance of a gene to inactivation by identifying marked depletions of predicted LoF variation^4,7^. Here, we present a refined mutational model, which incorporates methylation, base-level coverage correction, and LOFTEE (Supplementary Information, Extended Data Fig. 6), to predict expected levels of variation under neutrality. Under this updated model, the variation in the number of synonymous variants observed is accurately captured (*r* = 0.979). We then applied this method to detect depletion of pLoF variation by comparing the number of observed pLoF variants against our expectation in the gnomAD exome data from 125,748 individuals—more than doubling the sample size of ExAC, the previously largest exome collection^4^. For this dataset, we computed a median of 17.9 expected pLoF variants per gene (Fig. 2c) and found that 72.1% of genes have more than 10 pLoF variants (powered to be classified into the most constrained genes) (Supplementary Information) expected on the canonical transcript (Fig. 2d), an increase from 13.2% and 62.8%, respectively, in ExAC.

**Extended Data Fig. 7:**
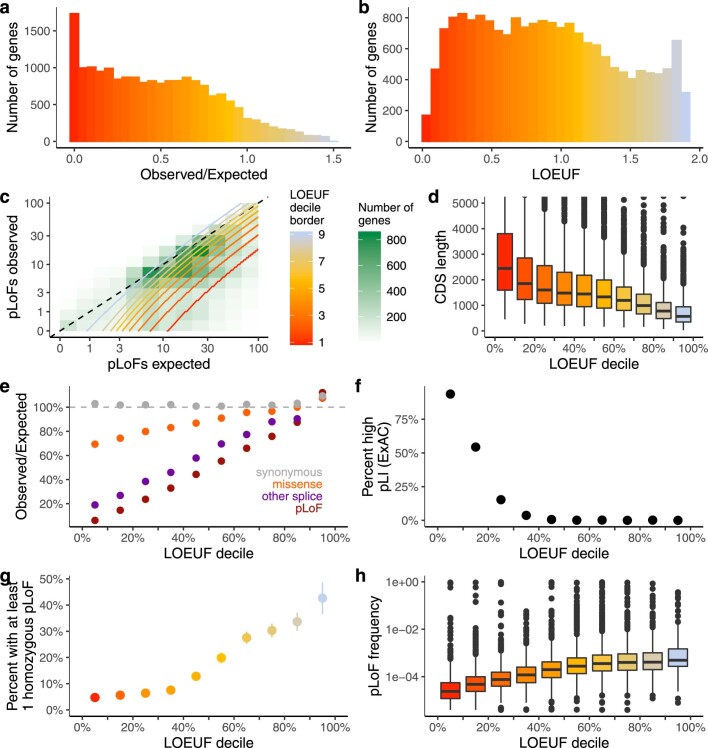
Genomic properties of constrained genes. **a**, **b**, Histogram of the observed/expected ratio of pLoF variation (**a**) and LOEUF (**b**). Most genes have fewer observed variants than expected (median observed/expected = 0.48), and the genes with no observed pLoFs are distinguished between confidently constrained genes and noise by LOEUF. **c**, A 2D density plot of the number of observed versus expected pLoF variants. The boundaries of each decile are plotted as gradients (that is, the most constrained decile is below the lowest red line). **d**, The LOEUF of a gene is correlated with its coding sequence length (beta = −1.07 × 10^−^^4^; *P* < 10^−100^): thus, for all downstream statistical tests, we adjust for gene length or remove genes with fewer than 10 expected pLoFs. **e**, Observed/expected ratios of various functional classes across genes within each LOEUF decile. The most constrained decile has approximately 6% of the expected pLoFs, while synonymous variants are not depleted and missense variants exhibit modest depletion. **f**, The percentage of each LOEUF decile that was described in ExAC as constrained, or pLI > 0.9^4^. **g**, The percentage of each LOEUF decile that have at least one homozygous pLoF variant. **h**, Box plots of the aggregate pLoF frequency for each LOEUF decile. Centre line denotes the median; box limits denote upper and lower quartiles; whiskers denote 1.5× the interquartile range; points denote outliers). In **e**–**g**, error bars represent 95% confidence intervals (note that in some cases these are fully contained within the plotted point).

The smaller sample size in ExAC required a transformation of the observed and expected values for the number of pLoF variants in each gene into the pLI: this metric estimates the probability that a gene falls into the class of LoF-haploinsufficient genes (approximately 10% observed/expected variation) and is ideally used as a dichotomous metric (producing 3,230 genes with pLI > 0.9). Here, our refined model and substantially increased sample size enabled us to directly assess the degree of intolerance to pLoF variation in each gene using the continuous metric of the observed/expected ratio and to estimate a confidence interval around the ratio. We find that the median observed/expected ratio is 48%, which indicates that, as noted previously, most genes exhibit at least moderate selection against pLoF variation, and that the distribution of the observed/expected ratio is not dichotomous, but continuous (Extended Data Fig. 7a). For downstream analyses, unless otherwise specified, we use the 90% upper bound of this confidence interval, which we term the loss-of-function observed/expected upper bound fraction (LOEUF) (Extended Data Fig. 7b, c), and bin 19,197 genes into deciles of approximately 1,920 genes each. At current sample sizes, this metric enables the quantitative assessment of constraint with a built-in confidence value, and distinguishes small genes (for example, those with observed = 0, expected = 2; LOEUF = 1.34) from large genes (for example, observed = 0, expected = 100; LOEUF = 0.03), while retaining the continuous properties of the direct estimate of the ratio (Supplementary Information). At one extreme of the distribution, we observe genes with a very strong depletion of pLoF variation (first LOEUF decile aggregate observed/expected approximately 6%) (Extended Data Fig. 7e), including genes previously characterized as high pLI (Extended Data Fig. 7f). By contrast, we find unconstrained genes that are relatively tolerant of inactivation, including many that contain homozygous pLoF variants (Extended Data Fig. 7g).

We note that the use of the upper bound means that LOEUF is a conservative metric in one direction: genes with low LOEUF scores are confidently depleted for pLoF variation, whereas genes with high LOEUF scores are a mixture of genes without depletion, and genes that are too small to obtain a precise estimate of the observed/expected ratio. In general, however, the scale of gnomAD means that gene length is rarely a substantive confounder for the analyses described here, and all downstream analyses are adjusted for the length of the coding sequence or filtered to genes with at least ten expected pLoFs (Supplementary Information).

## Validation of the LoF-intolerance score

**Fig. 3:**
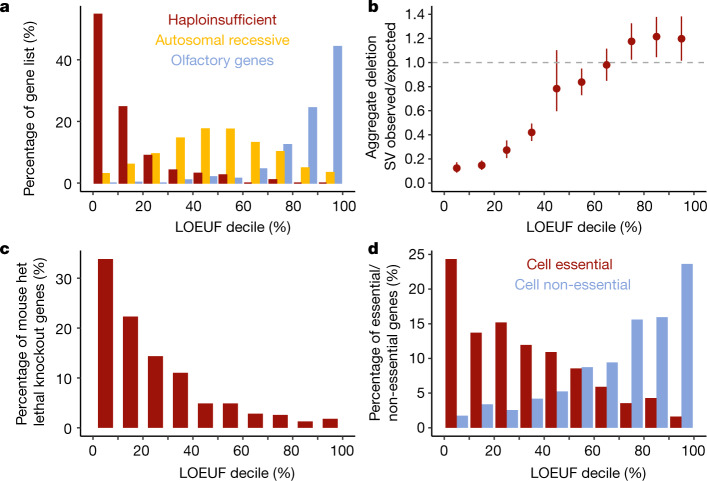
The functional spectrum of pLoF impact. **a**, The percentage of genes in a set of curated gene lists represented in each LOEUF decile. Haploinsufficient genes are enriched among the most constrained genes, whereas recessive genes are spread in the middle of the distribution, and olfactory receptor genes are largely unconstrained. **b**, The occurrence of 6,735 rare LoF deletion structural variants (SVs) is correlated with LOEUF (computed from SNVs; linear regression *r* = 0.13; *P* = 9.8 × 10^−68^). Error bars represent 95% confidence intervals from bootstrapping. **c**, **d**, Constrained genes are more likely to be lethal when heterozygously inactivated in mouse and cause cellular lethality when disrupted in human cells (**c**), whereas unconstrained genes are more likely to be tolerant of disruption in cellular models (**d**). For all panels, more constrained genes are shown on the left.

The LOEUF metric allows us to place each gene along a continuous spectrum of tolerance to inactivation. We examined the correlation of this metric with several independent measures of genic sensitivity to disruption. First, we found that LOEUF is consistent with the expected behaviour of well-established gene sets: known haploinsufficient genes are strongly depleted of pLoF variation, whereas olfactory receptors are relatively unconstrained, and genes with a known autosomal recessive mechanism, for which selection against heterozygous disruptive variants tends to be present but weak^9^, fall in the middle of the distribution (Fig. 3a). In addition, LOEUF is positively correlated with the occurrence of 6,735 rare autosomal deletion structural variants overlapping protein-coding exons identified in a subset of 6,749 individuals with whole-genome sequencing data in this manuscript^11^ (*r* = 0.13; *P* = 9.8 × 10^−68^) (Fig. 3b).

This constraint metric also correlates with results in model systems: in 389 genes with orthologues that are embryonically lethal after heterozygous deletion in mouse^21,22^, we find a lower LOEUF score (mean = 0.488), compared with the remaining 18,808 genes (mean = 0.962; *t*-test *P* = 10^−78^) (Fig. 3c). Similarly, the 678 genes that are essential for human cell viability as characterized by CRISPR screens^23^ are also depleted for pLoF variation (mean LOEUF = 0.63) in the general population compared to background (18,519 genes with mean LOEUF = 0.964; *t*-test *P* = 9 × 10^−71^), whereas the 777 non-essential genes are more likely to be unconstrained (mean LOEUF = 1.34, compared to remaining 18,420 genes with mean LOEUF = 0.936; *t*-test *P* = 3 × 10^−92^) (Fig. 3d).

## Biological properties of constraint

**Fig. 4:**
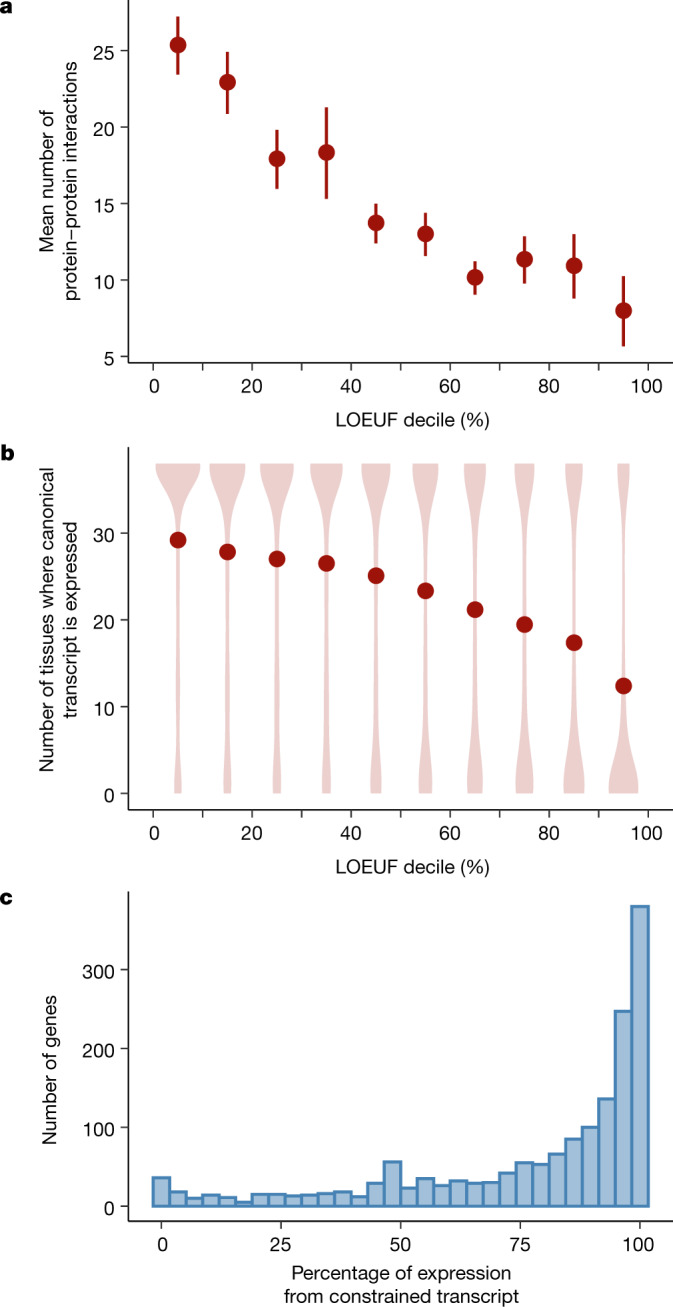
Biological properties of constrained genes and transcripts. **a**, The mean number of protein–protein interactions is plotted as a function of LOEUF decile: more constrained genes have more interaction partners (LOEUF linear regression *r* = −0.14; *P* = 1.7 × 10^−51^). Error bars correspond to 95% confidence intervals. **b**, The number of tissues where a gene is expressed (transcripts per million > 0.3), binned by LOEUF decile, is shown as a violin plot with the mean number overlaid as points: more constrained genes are more likely to be expressed in several tissues (LOEUF linear regression *r* = −0.31; *P* < 1 × 10^−100^). **c**, For 1,740 genes in which there exists at least one constrained and one unconstrained transcript, the proportion of expression derived from the constrained transcript is plotted as a histogram.

**Extended Data Fig. 8:**
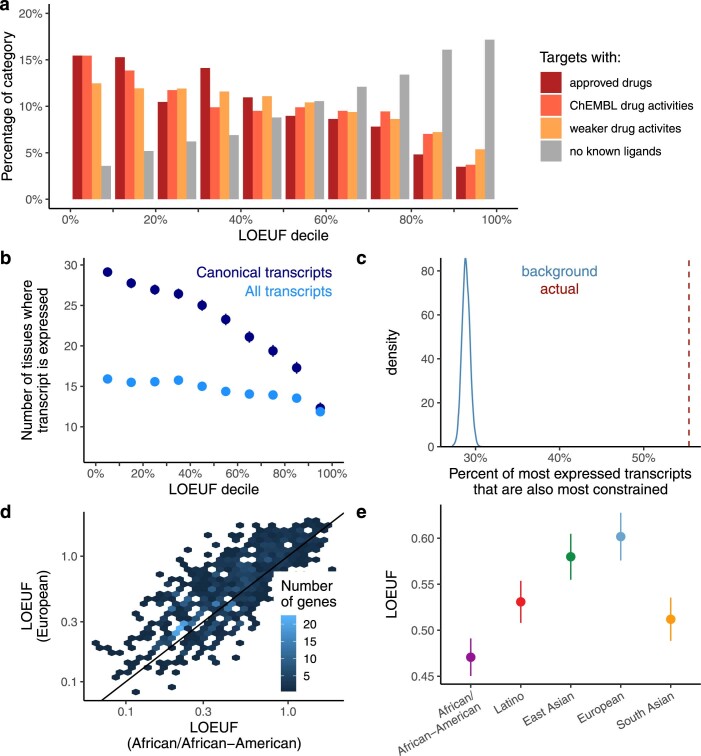
Biological properties of constrained genes. **a**, The percentage of genes in each functional category from Pharos (see Supplementary Information) is broken down by the LOEUF decile. **b**, The mean number of tissues in which a transcript is expressed, binned by transcript-based LOEUF decile, is shown for all transcripts and canonical transcripts. **c**, The percentage of genes in which the most expressed transcript is also the most constrained is plotted in red, which is enriched compared to a permuted set (blue). **d**, For 927 genes with expected pLoF ≥10 in both the African/African American and European population subsets (*n* = 8,128), the LOEUF scores are highly correlated (linear regression *r* = 0.78, *P* < 10^−100^), with a lower mean score observed in the African/African American population (0.49 versus 0.62; two-sided *t*-test *P* = 4.1 × 10^−14^), which has a higher effective population size. **e**, The mean LOEUF score for 865 genes with expected pLoF ≥ 10 in all populations (*n* = 8,128). Error bars represent 95% confidence intervals.

We investigated the properties of genes and transcripts as a function of their tolerance to pLoF variation (LOEUF). First, we found that LOEUF correlates with the degree of connection of a gene in protein-interaction networks (*r* = −0.14; *P* = 1.7 × 10^−51^ after adjusting for gene length) (Fig. 4a) and functional characterization (Extended Data Fig. 8a). In addition, constrained genes are more likely to be ubiquitously expressed across 38 tissues in the Genotype-Tissue Expression (GTEx) project (Fig. 4b) (LOEUF *r* = −0.31; *P* < 1 × 10^−100^) and have higher expression on average (LOEUF *ρ* = −0.28; *P* < 1 × 10^−100^), consistent with previous results^4^. Although most results in this study are reported at the gene level, we have also extended our framework to compute LOEUF for all protein-coding transcripts, allowing us to explore the extent of differential constraint of transcripts within a given gene. In cases in which a gene contained transcripts with varying levels of constraint, we found that transcripts in the first LOEUF decile were more likely to be expressed across tissues than others in the same gene (*n* = 1,740 genes), even when adjusted for transcript length (Fig. 4c) (constrained transcripts are on average 6.34 transcripts per million higher; *P* = 2.2 × 10^−14^). Furthermore, we found that the most constrained transcript for each gene was typically the most highly expressed transcript in tissues with disease relevance^24^ (Extended Data Fig. 8c), which supports the need for transcript-based variant interpretation, as explored in more depth in an accompanying manuscript^15^.

Finally, we investigated potential differences in LOEUF across human populations, restricting to the same sample size across all populations to remove bias due to differential power for variant discovery. As the smallest population in our exome dataset (African/African American) has only 8,128 individuals, our ability to detect constraint against pLoF variants for individual genes is limited. However, for well-powered genes (expected pLoF ≥ 10) (Supplementary Information), we observed a lower mean observed/expected ratio and LOEUF across genes among African/African American individuals, a population with a larger effective population size, compared with other populations (Extended Data Fig. 8d, e), consistent with the increased efficiency of selection in populations with larger effective population sizes^25,26^.

## Constraint informs disease aetiologies

**Extended Data Fig. 9:**
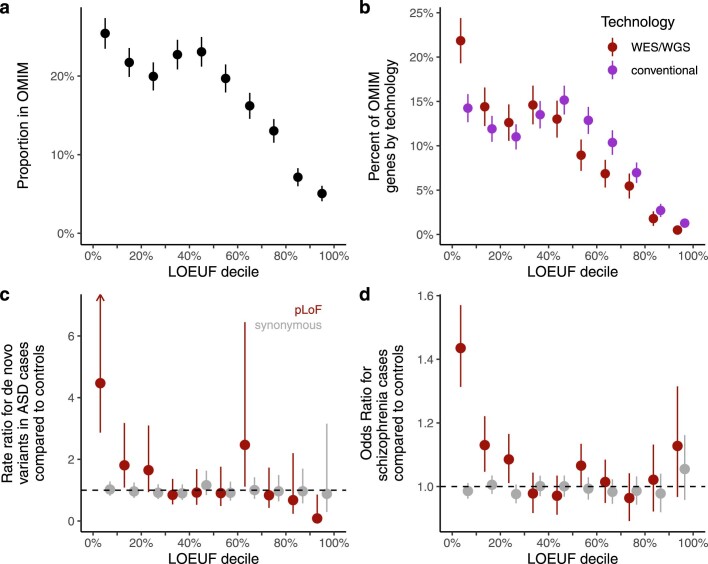
Applications of constraint metrics to rare variant analysis of disease. **a**, Proportion of each LOEUF decile found in OMIM. **b**, Proportion of disease-associated genes discovered by whole-exome/genome sequencing (WES/WGS) compared to conventional (typically linkage) methods, plotted by LOEUF decile. The former are more constrained (LOEUF 0.674 versus 0.806, two-sided *t*-test *P* = 1.2 × 10^−16^), which suggests that these techniques are more effective for picking up genes with a de novo mechanism of disease, compared to recessive genes identified by linkage methods. **c**, Similar to Fig. 5a, the rate ratio is defined by the rate of de novo variants (number per patient) in autism cases divided by the rate in controls. pLoF variants in the most constrained decile of the genome are approximately fourfold more likely to be found in cases compared to controls. **d**, The mean odds ratio of a logistic regression of schizophrenia^28^ is plotted for each LOEUF decile. Error bars in **a**–**d** correspond to 95% confidence intervals.

The LOEUF metric can be applied to improve molecular diagnosis and advance our understanding of disease mechanisms. Disease-associated genes, discovered by different technologies over the course of many years across all categories of inheritance and effects, span the entire spectrum of LoF tolerance (Extended Data Fig. 9a). However, in recent years, high-throughput sequencing technologies have enabled the identification of highly deleterious variants that are de novo or only inherited in small families or trios, leading to the discovery of novel disease genes under extreme constraint against pLoF variation that could not have been identified by linkage approaches that rely on broadly inherited variation (Extended Data Fig. 9b). This result is consistent with a recent analysis that shows a post-whole-exome/whole-genome sequencing era enrichment for gene–disease relationships attributable to de novo variants^27^.

**Fig. 5:**
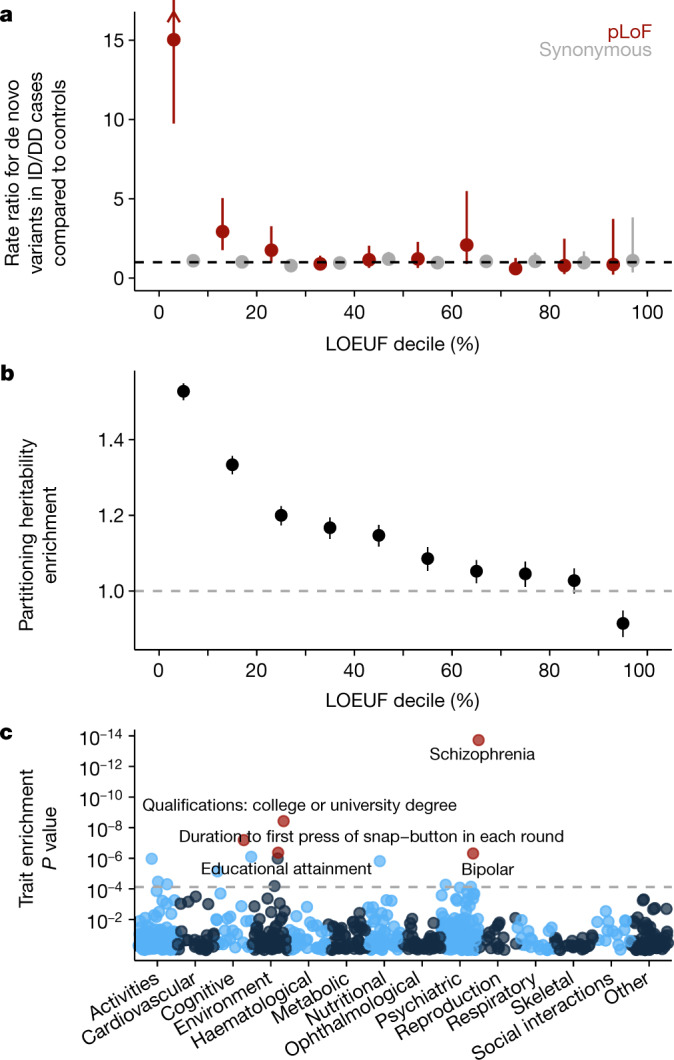
Disease applications of constraint. **a**, The rate ratio is defined by the rate of de novo variants (number per patient) in 5,305 cases of intellectual disability/developmental delay (ID/DD) divided by the rate in 2,179 controls. pLoF variants in the most constrained decile of the genome are approximately 11-fold more likely to be found in cases compared to controls. Error bars represent 95% confidence intervals. **b**, Marginal enrichment in per-SNV heritability explained by common (minor allele frequency > 5%) variants within 100-kb of genes in each LOEUF decile, estimated by linkage disequilibrium (LD) score regression^48^. Enrichment is compared to the average SNV genome-wide. The results reported here are from random effects meta-analysis of 276 independent traits (subsetted from the 658 traits with UK Biobank or large-scale consortium GWAS results). Error bars represent 95% confidence intervals. **c**, Conditional enrichment in per-SNV common variant heritability tested using regression of linkage disequilibrium score in each of 658 common disease and trait GWAS results. *P* values evaluate whether per-SNV heritability is proportional to the LOEUF of the nearest gene, conditional on 75 existing functional, linkage disequilibrium, and minor-allele-frequency-related genomic annotations. Colours alternate by broad phenotype category.

Rare variants, which are more likely to be deleterious, are expected to exhibit stronger effects on average in constrained genes (previously shown using pLI from ExAC^28^), with an effect size related to the severity and reproductive fitness of the phenotype. In an independent cohort of 5,305 individuals with intellectual disability or developmental disorders and 2,179 controls, the rate of pLoF de novo variation in cases is 15-fold higher in genes belonging to the most constrained LOEUF decile, compared with controls (Fig. 5a), with a slightly increased rate (2.9-fold) in the second highest decile but not in others. A similar, but attenuated enrichment (4.4-fold in the most constrained decile) is seen for de novo variants in 6,430 patients with autism spectrum disorder (Extended Data Fig. 9c). Furthermore, in burden tests of rare variants (allele count across both cases and controls = 1) of patients with schizophrenia^28^, we find a significantly higher odds ratio in constrained genes (Extended Data Fig. 9d).

**Extended Data Fig. 10:**
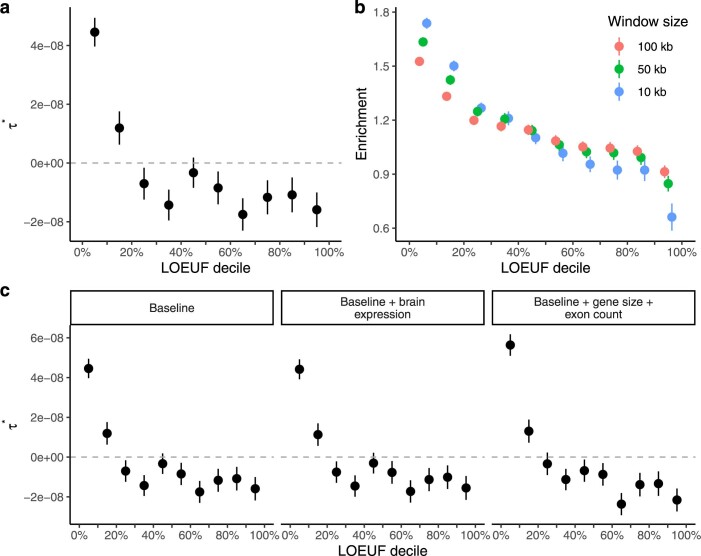
Applications of constraint metrics to common variant analysis of disease. **a**, The ${\hat{{\rm{\tau }}}}^{\ast }$ coefficient (see Supplementary Information) for each LOEUF decile across 276 independent traits. Unlike the enrichment measure reported in Fig. 5, ${\hat{{\rm{\tau }}}}^{\ast }$is adjusted for 74 baseline genomics annotations. Positive values of ${\hat{{\rm{\tau }}}}^{\ast }$ indicate greater per-SNP heritability than would be expected based on the other annotations in the baseline model, whereas negative values indicate depleted per-SNP heritability compared to that baseline expectation. **b**, Enrichment coefficient for each LOEUF decile using different window sizes to define which SNPs to include upstream and downstream of each gene. **c**, Enrichment coefficient for each LOEUF decile across traits after controlling for brain expression and gene size. Results are consistent with those shown in Fig. 5, which indicates that brain gene expression and gene size do not fully explain the enrichment of heritability observed in constrained genes. Error bars represent 95% confidence intervals.

Finally, although pLoF variants are predominantly rare, other more common variation in constrained genes may also be deleterious, including the effects of other coding or regulatory variants. In a heritability partitioning analysis of association results for 658 traits in the UK Biobank and other large-scale genome-wide association study (GWAS) efforts, we find an enrichment of common variant associations near genes that is linearly related to LOEUF decile across numerous traits (Fig. 5b). Schizophrenia and educational attainment are the most enriched traits (Fig. 5c), consistent with previous observations in associations between rare pLoF variants and these phenotypes^29–31^. This enrichment persists even when accounting for gene size, expression in GTEx brain samples, and previously tested annotations of functional regions and evolutionary conservation, and suggests that some heritable polygenic diseases and traits, particularly cognitive or psychiatric ones, have an underlying genetic architecture that is driven substantially by constrained genes (Extended Data Fig. 10).

## Discussion

In this paper and accompanying publications, we present the largest, to our knowledge, catalogue of harmonized variant data from any species so far, incorporating exome or genome sequence data from more than 140,000 humans. The gnomAD dataset of over 270 million variants is publicly available (https://gnomad.broadinstitute.org), and has already been widely used as a resource for estimates of allele frequency in the context of rare disease diagnosis (for a recent review, see Eilbeck et al.^32^), improving power for disease gene discovery^33–35^, estimating genetic disease frequencies^36,37^, and exploring the biological effect of genetic variation^38,39^. Here, we describe the application of this dataset to calculate a continuous metric that describes a spectrum of tolerance to pLoF variation for each protein-coding gene in the human genome. We validate this method using known gene sets and data from model organisms, and explore the value of this metric for investigating human gene function and discovery of disease genes.

We have focused on high-confidence, high-impact pLoF variants, calibrating our analysis to be highly specific to compensate for the increased false-positive rate among deleterious variants. However, some additional error modes may still exist, and indeed, several recent experiments have proposed uncharacterized mechanisms for escape from nonsense-mediated mRNA decay^40,41^. Furthermore, such a stringent approach will remove some true positives. For example, terminal truncations that are removed by LOFTEE may still exert a LoF mechanism through the removal of crucial C-terminal domains, despite the escape of the gene from nonsense-mediated decay. In addition, current annotation tools are incapable of detecting all classes of LoF variation and typically miss, for instance, missense variants that inactivate specific gene functions, as well as high-impact variants in regulatory regions. Future work will benefit from the increasing availability of high-throughput experimental assays that can assess the functional effect of all possible coding variants in a target gene^42^, although scaling these experimental assays to all protein-coding genes represents a huge challenge. Identifying constraint in individual regulatory elements outside coding regions will be even more challenging, and require much larger sample sizes of whole genomes as well as improved functional annotation^43^. We discuss one class of high-impact regulatory variants in a companion manuscript^17^, but many remain to be fully characterized.

Although the gnomAD dataset is of unprecedented scale, it has important limitations. At this sample size, we remain far from saturating all possible pLoF variants in the human exome; even at the most mutable sites in the genome (methylated CpG dinucleotides), we observe only half of all possible stop-gained variants. A substantial fraction of the remaining variants are likely to be heterozygous lethal, whereas others will exhibit an intermediate selection coefficient; much larger sample sizes (in the millions to hundreds of millions of individuals) will be required for comprehensive characterization of selection against all individual LoF variants in the human genome. Such future studies would also benefit substantially from increased ancestral diversity beyond the European-centric sampling of many current studies, which would provide opportunities to observe very rare and population-specific variation, as well as increase power to explore population differences in gene constraint. In particular, current reference databases including gnomAD have a near-complete absence of representation from the Middle East, central and southeast Asia, Oceania, and the vast majority of the African continent^44^, and these gaps must be addressed if we are to fully understand the distribution and effect of human genetic variation.

It is also important to understand the practical and evolutionary interpretation of pLoF constraint. In particular, it should be noted that these metrics primarily identify genes undergoing selection against heterozygous variation, rather than strong constraint against homozygous variation^45^. In addition, the power of the LOEUF metric is affected by gene length, with approximately 30% of the coding genes in the genome still insufficiently powered for detection of constraint even at the scale of gnomAD (Fig. 2d). Substantially larger sample sizes and careful analysis of individuals enriched for homozygous pLoFs (see below) will be useful for distinguishing these possibilities. Furthermore, selection is largely blind to phenotypes emerging after reproductive age, and thus genes with phenotypes that manifest later in life, even if severe or fatal, may exhibit much weaker intolerance to inactivation. Despite these caveats, our results demonstrate that pLoF constraint divides protein-coding genes in a way that correlates usefully with their probability of disease impact and other biological properties, and confirm the value of constraint in prioritizing candidate genes in studies of both rare and common diseases.

Examples such as *PCSK9* demonstrate the value of human pLoF variants for identifying and validating targets for therapeutic intervention across a wide range of human diseases. As discussed in more detail in an accompanying manuscript^12^, careful attention must be paid to a variety of complicating factors when using pLoF constraint to assess candidates. More valuable information comes from directly exploring the phenotypic effect of LoF variants on carrier humans, both through ‘forward genetics’ approaches such as gene mapping to identify genes that cause Mendelian disease, as well as ‘reverse genetics’ approaches that leverage large collections of sequenced humans to find and clinically characterize individuals with disruptive mutations in specific genes. Although clinical data are currently available for only a small subset of gnomAD individuals, future efforts that integrate sequencing and deep phenotyping of large biobanks will provide valuable insight into the biological implications of partial disruption of specific genes. This is illustrated in a companion manuscript that explores the clinical correlates of heterozygous pLoF variants in the *LRRK2* gene, demonstrating that life-long partial inactivation of this gene is likely to be safe in humans^13^.

Such examples, and the sheer scale of pLoF discovery in this dataset, suggest the near-future feasibility and considerable value of a human ‘knockout’ project—a systematic attempt to discover the phenotypic consequences of functionally disruptive mutations, in either the heterozygous or homozygous state, for all human protein-coding genes. Such an approach will require cohorts of samples from millions of sequenced and deeply, consistently phenotyped individuals and, for the discovery of ‘complete’ knockouts, would benefit substantially from the targeted inclusion of large numbers of samples from populations that have either experienced strong demographic bottlenecks or high levels of recent parental relatedness (consanguinity)^12^. Such a resource would allow the construction of a comprehensive map that directly links gene-disrupting variation to human biology.

### Reporting summary

Further information on research design is available in the Nature Research Reporting Summary linked to this paper.

## Online content

Any methods, additional references, Nature Research reporting summaries, source data, extended data, supplementary information, acknowledgements, peer review information; details of author contributions and competing interests; and statements of data and code availability are available at 10.1038/s41586-020-2308-7.

## Supplementary information

Supplementary Information This file contains Supplementary Methods and descriptions of Supplementary Analyses, including Supplementary Methods and Text, Supplementary Figures 1-11, Supplementary Tables 1-21, Data Availability, Supplementary References, and detailed descriptions of Supplementary Datasets.

Reporting Summary

Peer Review File Reviewer reports and authors' response from the peer review of this Article at Nature.

Supplementary Data This zipped file contains Supplementary Data items 1-14 - see Supplementary Information document for Supplementary Dataset guide.

## Data Availability

The gnomAD 2.1.1 dataset is available for download at http://gnomad.broadinstitute.org, where we have developed a browser for the dataset and provide files with detailed frequency and annotation information for each variant. There are no restrictions on the aggregate data released.

## Code availability

All code to perform quality control is provided at https://github.com/broadinstitute/gnomad_qc, and the code to perform all analyses and regenerate all the figures in this manuscript is provided at https://github.com/macarthur-lab/gnomad_lof. LOFTEE is available at https://github.com/konradjk/loftee. All code and software to reproduce figures are available in a Docker image at konradjk/gnomad_lof_paper:0.2.
