# Author Correction: The mutational constraint spectrum quantified from variation in 141,456 humans

**DOI:** 10.1038/s41586-020-03174-8

**Published:** 2021-02-03

**Authors:** Konrad J. Karczewski, Laurent C. Francioli, Grace Tiao, Beryl B. Cummings, Jessica Alföldi, Qingbo Wang, Ryan L. Collins, Kristen M. Laricchia, Andrea Ganna, Daniel P. Birnbaum, Laura D. Gauthier, Harrison Brand, Matthew Solomonson, Nicholas A. Watts, Daniel Rhodes, Moriel Singer-Berk, Eleina M. England, Eleanor G. Seaby, Jack A. Kosmicki, Raymond K. Walters, Katherine Tashman, Yossi Farjoun, Eric Banks, Timothy Poterba, Arcturus Wang, Cotton Seed, Nicola Whiffin, Jessica X. Chong, Kaitlin E. Samocha, Emma Pierce-Hoffman, Zachary Zappala, Anne H. O’Donnell-Luria, Eric Vallabh Minikel, Ben Weisburd, Monkol Lek, James S. Ware, Christopher Vittal, Irina M. Armean, Louis Bergelson, Kristian Cibulskis, Kristen M. Connolly, Miguel Covarrubias, Stacey Donnelly, Steven Ferriera, Stacey Gabriel, Jeff Gentry, Namrata Gupta, Thibault Jeandet, Diane Kaplan, Christopher Llanwarne, Ruchi Munshi, Sam Novod, Nikelle Petrillo, David Roazen, Valentin Ruano-Rubio, Andrea Saltzman, Molly Schleicher, Jose Soto, Kathleen Tibbetts, Charlotte Tolonen, Gordon Wade, Michael E. Talkowski, Genome Aggregation Database Consortium, Carlos A. Aguilar Salinas, Tariq Ahmad, Christine M. Albert, Diego Ardissino, Gil Atzmon, John Barnard, Laurent Beaugerie, Emelia J. Benjamin, Michael Boehnke, Lori L. Bonnycastle, Erwin P. Bottinger, Donald W. Bowden, Matthew J. Bown, John C. Chambers, Juliana C. Chan, Daniel Chasman, Judy Cho, Mina K. Chung, Bruce Cohen, Adolfo Correa, Dana Dabelea, Mark J. Daly, Dawood Darbar, Ravindranath Duggirala, Josée Dupuis, Patrick T. Ellinor, Roberto Elosua, Jeanette Erdmann, Tõnu Esko, Martti Färkkilä, Jose Florez, Andre Franke, Gad Getz, Benjamin Glaser, Stephen J. Glatt, David Goldstein, Clicerio Gonzalez, Leif Groop, Christopher Haiman, Craig Hanis, Matthew Harms, Mikko Hiltunen, Matti M. Holi, Christina M. Hultman, Mikko Kallela, Jaakko Kaprio, Sekar Kathiresan, Bong-Jo Kim, Young Jin Kim, George Kirov, Jaspal Kooner, Seppo Koskinen, Harlan M. Krumholz, Subra Kugathasan, Soo Heon Kwak, Markku Laakso, Terho Lehtimäki, Ruth J. F. Loos, Steven A. Lubitz, Ronald C. W. Ma, Daniel G. MacArthur, Jaume Marrugat, Kari M. Mattila, Steven McCarroll, Mark I. McCarthy, Dermot McGovern, Ruth McPherson, James B. Meigs, Olle Melander, Andres Metspalu, Benjamin M. Neale, Peter M. Nilsson, Michael C. O’Donovan, Dost Ongur, Lorena Orozco, Michael J. Owen, Colin N. A. Palmer, Aarno Palotie, Kyong Soo Park, Carlos Pato, Ann E. Pulver, Nazneen Rahman, Anne M. Remes, John D. Rioux, Samuli Ripatti, Dan M. Roden, Danish Saleheen, Veikko Salomaa, Nilesh J. Samani, Jeremiah Scharf, Heribert Schunkert, Moore B. Shoemaker, Pamela Sklar, Hilkka Soininen, Harry Sokol, Tim Spector, Patrick F. Sullivan, Jaana Suvisaari, E. Shyong Tai, Yik Ying Teo, Tuomi Tiinamaija, Ming Tsuang, Dan Turner, Teresa Tusie-Luna, Erkki Vartiainen, Marquis P. Vawter, James S. Ware, Hugh Watkins, Rinse K. Weersma, Maija Wessman, James G. Wilson, Ramnik J. Xavier, Benjamin M. Neale, Mark J. Daly, Daniel G. MacArthur

**Affiliations:** 1 Program in Medical and Population Genetics, Broad Institute of MIT and Harvard, Cambridge, MA USA; 2 Analytic and Translational Genetics Unit, Massachusetts General Hospital, Boston, MA USA; 3 Program in Biological and Biomedical Sciences, Harvard Medical School, Boston, MA USA; 4 Program in Bioinformatics and Integrative Genomics, Harvard Medical School, Boston, MA USA; 5 Center for Genomic Medicine, Massachusetts General Hospital, Boston, MA USA; 6 Institute for Molecular Medicine Finland, Helsinki, Finland; 7 Data Sciences Platform, Broad Institute of MIT and Harvard, Cambridge, MA USA; 8 Centre for Translational Bioinformatics, William Harvey Research Institute, Barts and the London School of Medicine and Dentistry, Queen Mary University of London and Barts Health NHS Trust, London, UK; 9 Stanley Center for Psychiatric Research, Broad Institute of MIT and Harvard, Cambridge, MA USA; 10 National Heart & Lung Institute and MRC London Institute of Medical Sciences, Imperial College London, London, UK; 11 Cardiovascular Research Centre, Royal Brompton & Harefield Hospitals NHS Trust, London, UK; 12 Department of Pediatrics, University of Washington, Seattle, WA USA; 13 Wellcome Sanger Institute, Wellcome Genome Campus, Hinxton, Cambridge UK; 14 Vertex Pharmaceuticals Inc, Boston, MA USA; 15 Division of Genetics and Genomics, Boston Children’s Hospital, Boston, MA USA; 16 Department of Pediatrics, Harvard Medical School, Boston, MA USA; 17 Department of Genetics, Yale School of Medicine, New Haven, CT USA; 18 Broad Genomics, Broad Institute of MIT and Harvard, Cambridge, MA USA; 19 Department of Neurology, Harvard Medical School, Boston, MA USA; 150 Present Address: Centre for Population Genomics, Garvan Institute of Medical Research, and UNSW Sydney, Sydney, New South Wales Australia; 151 Present Address: Centre for Population Genomics, Murdoch Children’s Research Institute, Melbourne, Victoria Australia; 20 Unidad de Investigacion de Enfermedades Metabolicas, Instituto Nacional de Ciencias Medicas y Nutricion, Mexico City, Mexico; 21 Peninsula College of Medicine and Dentistry, Exeter, UK; 22 Division of Preventive Medicine, Brigham and Women’s Hospital, Boston, MA USA; 23 Division of Cardiovascular Medicine, Brigham and Women’s Hospital and Harvard Medical School, Boston, MA USA; 24 Department of Cardiology, University Hospital, Parma, Italy; 25 Department of Biology, Faculty of Natural Sciences, University of Haifa, Haifa, Israel; 26 Department of Medicine, Albert Einstein College of Medicine, Bronx, NY USA; 27 Department of Genetics, Albert Einstein College of Medicine, Bronx, NY USA; 28 Department of Quantitative Health Sciences, Lerner Research Institute, Cleveland Clinic, Cleveland, OH USA; 29 Sorbonne Université, APHP, Gastroenterology Department, Saint Antoine Hospital, Paris, France; 30 Framingham Heart Study, National Heart, Lung, & Blood Institute and Boston University, Framingham, MA USA; 31 Department of Medicine, Boston University School of Medicine, Boston, MA USA; 32 Department of Epidemiology, Boston University School of Public Health, Boston, MA USA; 33 Department of Biostatistics, Center for Statistical Genetics, University of Michigan, Ann Arbor, MI USA; 34 National Human Genome Research Institute, National Institutes of Health, Bethesda, MD USA; 35 The Charles Bronfman Institute for Personalized Medicine, Icahn School of Medicine at Mount Sinai, New York, NY USA; 36 Department of Biochemistry, Wake Forest School of Medicine, Winston-Salem, NC USA; 37 Center for Genomics and Personalized Medicine Research, Wake Forest School of Medicine, Winston-Salem, NC USA; 38 Center for Diabetes Research, Wake Forest School of Medicine, Winston-Salem, NC USA; 39 Department of Cardiovascular Sciences, University of Leicester, Leicester, UK; 40 NIHR Leicester Biomedical Research Centre, Glenfield Hospital, Leicester, UK; 41 Department of Epidemiology and Biostatistics, Imperial College London, London, UK; 42 Department of Cardiology, Ealing Hospital NHS Trust, Southall, UK; 43 Imperial College Healthcare NHS Trust, Imperial College London, London, UK; 44 Department of Medicine and Therapeutics, The Chinese University of Hong Kong, Hong Kong, China; 45 Department of Medicine, Harvard Medical School, Boston, MA USA; 46 Program for Neuropsychiatric Research, McLean Hospital, Belmont, MA USA; 47 Department of Medicine, University of Mississippi Medical Center, Jackson, MI USA; 48 Department of Epidemiology, Colorado School of Public Health, Aurora, CO USA; 49 Department of Medicine and Pharmacology, University of Illinois at Chicago, Chicago, IL USA; 50 Department of Genetics, Texas Biomedical Research Institute, San Antonio, TX USA; 51 Department of Biostatistics, Boston University School of Public Health, Boston, MA USA; 52 Cardiac Arrhythmia Service and Cardiovascular Research Center, Massachusetts General Hospital, Boston, MA USA; 53 Cardiovascular Epidemiology and Genetics, Hospital del Mar Medical Research Institute (IMIM), Barcelona, Catalonia Spain; 54 Centro de Investigación Biomédica en Red Enfermedades Cardiovaculares (CIBERCV), Barcelona, Catalonia Spain; 55 Department of Medicine, Medical School, University of Vic-Central University of Catalonia, Vic, Catalonia Spain; 56 Institute for Cardiogenetics, University of Lübeck, Lübeck, Germany; 57 DZHK (German Research Centre for Cardiovascular Research), partner site Hamburg/Lübeck/Kiel, Lübeck, Germany; 58 University Heart Center Lübeck, Lübeck, Germany; 59 Estonian Genome Center, Institute of Genomics, University of Tartu, Tartu, Estonia; 60 Helsinki University and Helsinki University Hospital, Clinic of Gastroenterology, Helsinki, Finland; 61 Diabetes Unit, Massachusetts General Hospital, Boston, MA USA; 62 Center for Genomic Medicine, Massachusetts General Hospital, Boston, MA USA; 63 Program in Metabolism, Broad Institute of MIT and Harvard, Cambridge, MA USA; 64 Institute of Clinical Molecular Biology (IKMB), Christian-Albrechts-University of Kiel, Kiel, Germany; 65 Bioinformatics Consortium, Massachusetts General Hospital, Boston, MA USA; 66 Cancer Genome Computational Analysis, Broad Institute of MIT and Harvard, Cambridge, MA USA; 67 Department of Pathology, Massachusetts General Hospital, Boston, MA USA; 68 Cancer Center, Massachusetts General Hospital, Boston, MA USA; 69 Endocrinology and Metabolism Department, Hadassah-Hebrew University Medical Center, Jerusalem, Israel; 70 Department of Psychiatry and Behavioral Sciences, SUNY Upstate Medical University, Syracuse, NY USA; 71 Institute for Genomic Medicine, Columbia University Medical Center, Hammer Health Sciences, New York, NY USA; 72 Department of Genetics and Development, Columbia University Medical Center, Hammer Health Sciences, New York, NY USA; 73 Centro de Investigacion en Salud Poblacional, Instituto Nacional de Salud Publica, Cuernavaca, Mexico; 74 Genomics, Diabetes and Endocrinology, Lund University, Lund, Sweden; 75 Lund University Diabetes Centre, Malmö, Sweden; 76 Human Genetics Center, University of Texas Health Science Center at Houston, Houston, TX USA; 77 Department of Neurology, Columbia University, New York, NY USA; 78 Institute of Genomic Medicine, Columbia University, New York, NY USA; 79 Institute of Biomedicine, University of Eastern Finland, Kuopio, Finland; 80 Department of Psychiatry, Helsinki University Central Hospital, Lapinlahdentie, Helsinki, Finland; 81 Department of Medical Epidemiology and Biostatistics, Karolinska Institutet, Stockholm, Sweden; 82 Icahn School of Medicine at Mount Sinai, New York, NY USA; 83 Department of Neurology, Helsinki University Central Hospital, Helsinki, Finland; 84 Department of Public Health, Faculty of Medicine, University of Helsinki, Helsinki, Finland; 85 Cardiovascular Disease Initiative and Program in Medical and Population Genetics, Broad Institute of MIT and Harvard, Cambridge, MA USA; 86 Center for Genome Science, Korea National Institute of Health, Chungcheongbuk-do, South Korea; 87 MRC Centre for Neuropsychiatric Genetics & Genomics, Cardiff University School of Medicine, Cardiff, UK; 88 Department of Health, National Institute for Health and Welfare (THL), Helsinki, Finland; 89 Section of Cardiovascular Medicine, Department of Internal Medicine, Yale School of Medicine, New Haven, CT USA; 90 Division of Pediatric Gastroenterology, Emory University School of Medicine, Atlanta, GA USA; 91 Department of Internal Medicine, Seoul National University Hospital, Seoul, South Korea; 92 Institute of Clinical Medicine, The University of Eastern Finland, Kuopio, Finland; 93 Kuopio University Hospital, Kuopio, Finland; 94 Department of Clinical Chemistry, Fimlab Laboratories and Finnish Cardiovascular Research Center-Tampere, Faculty of Medicine and Health Technology, Tampere University, Tampere, Finland; 95 The Mindich Child Health and Development Institute, Icahn School of Medicine at Mount Sinai, New York, NY USA; 96 Li Ka Shing Institute of Health Sciences, The Chinese University of Hong Kong, Hong Kong, China; 97 Hong Kong Institute of Diabetes and Obesity, The Chinese University of Hong Kong, Hong Kong, China; 98 Cardiovascular Research REGICOR Group, Hospital del Mar Medical Research Institute (IMIM), Barcelona, Catalonia Spain; 99 Department of Genetics, Harvard Medical School, Boston, MA USA; 100 Oxford Centre for Diabetes, Endocrinology and Metabolism, University of Oxford, Churchill Hospital, Headington, Oxford UK; 101 Wellcome Centre for Human Genetics, University of Oxford, Oxford, UK; 102 Oxford NIHR Biomedical Research Centre, Oxford University Hospitals NHS Foundation Trust, John Radcliffe Hospital, Oxford, UK; 103 F Widjaja Foundation Inflammatory Bowel and Immunobiology Research Institute, Cedars-Sinai Medical Center, Los Angeles, CA USA; 104 Atherogenomics Laboratory, University of Ottawa Heart Institute, Ottawa, Canada; 105 Division of General Internal Medicine, Massachusetts General Hospital, Boston, MA USA; 106 Department of Clinical Sciences, University Hospital Malmo Clinical Research Center, Lund University, Malmo, Sweden; 107 Department of Clinical Sciences, Lund University, Skane University Hospital, Malmo, Sweden; 108 Instituto Nacional de Medicina Genómica (INMEGEN), Mexico City, Mexico; 109 Medical Research Institute, Ninewells Hospital and Medical School, University of Dundee, Dundee, UK; 110 Department of Molecular Medicine and Biopharmaceutical Sciences, Graduate School of Convergence Science and Technology, Seoul National University, Seoul, South Korea; 111 Department of Psychiatry, Keck School of Medicine at the University of Southern California, Los Angeles, CA USA; 112 Department of Psychiatry and Behavioral Sciences, Johns Hopkins University School of Medicine, Baltimore, MD USA; 113 Division of Genetics and Epidemiology, Institute of Cancer Research, London, UK; 114 Medical Research Center, Oulu University Hospital, Oulu, Finland and Research Unit of Clinical Neuroscience, Neurology, University of Oulu, Oulu, Finland; 115 Research Center, Montreal Heart Institute, Montreal, Quebec Canada; 116 Department of Medicine, Faculty of Medicine, Université de Montréal, Quebec, Canada; 117 Department of Biomedical Informatics, Vanderbilt University Medical Center, Nashville, TN USA; 118 Department of Medicine, Vanderbilt University Medical Center, Nashville, TN USA; 119 Department of Biostatistics and Epidemiology, Perelman School of Medicine at the University of Pennsylvania, Philadelphia, PA USA; 120 Department of Medicine, Perelman School of Medicine at the University of Pennsylvania, Philadelphia, PA USA; 121 Center for Non-Communicable Diseases, Karachi, Pakistan; 122 National Institute for Health and Welfare, Helsinki, Finland; 123 Deutsches Herzzentrum München, Munich, Germany; 124 Technische Universität München, Munich, Germany; 125 Division of Cardiovascular Medicine, Nashville VA Medical Center and Vanderbilt University, School of Medicine, Nashville, TN USA; 126 Department of Psychiatry, Icahn School of Medicine at Mount Sinai, New York, NY USA; 127 Department of Genetics and Genomic Sciences, Icahn School of Medicine at Mount Sinai, New York, NY USA; 128 Institute for Genomics and Multiscale Biology, Icahn School of Medicine at Mount Sinai, New York, NY USA; 129 Institute of Clinical Medicine, Neurology, University of Eastern Finlad, Kuopio, Finland; 130 Department of Twin Research and Genetic Epidemiology, King’s College London, London, UK; 131 Departments of Genetics and Psychiatry, University of North Carolina, Chapel Hill, NC USA; 132 Saw Swee Hock School of Public Health, National University of Singapore, National University Health System, Singapore, Singapore; 133 Department of Medicine, Yong Loo Lin School of Medicine, National University of Singapore, Singapore, Singapore; 134 Duke-NUS Graduate Medical School, Singapore, Singapore; 135 Life Sciences Institute, National University of Singapore, Singapore, Singapore; 136 Department of Statistics and Applied Probability, National University of Singapore, Singapore, Singapore; 137 Folkhälsan Institute of Genetics, Folkhälsan Research Center, Helsinki, Finland; 138 HUCH Abdominal Center, Helsinki University Hospital, Helsinki, Finland; 139 Center for Behavioral Genomics, Department of Psychiatry, University of California, San Diego, CA USA; 140 Institute of Genomic Medicine, University of California, San Diego, CA USA; 141 Juliet Keidan Instit.ute of Pediatric Gastroenterology, Shaare Zedek Medical Center, The Hebrew University of Jerusalem, Jerusalem, Israel; 142 Instituto de Investigaciones Biomédicas UNAM, Mexico City, Mexico; 143 Instituto Nacional de Ciencias Médicas y Nutrición Salvador Zubirán, Mexico City, Mexico; 144 Department of Psychiatry & Human Behavior, University of California Irvine, Irvine, CA USA; 145 Radcliffe Department of Medicine, University of Oxford, Oxford, UK; 146 Department of Gastroenterology and Hepatology, University of Groningen and University Medical Center Groningen, Groningen, The Netherlands; 147 Department of Physiology and Biophysics, University of Mississippi Medical Center, Jackson, MS USA; 148 Program in Infectious Disease and Microbiome, Broad Institute of MIT and Harvard, Cambridge, MA USA; 149 Center for Computational and Integrative Biology, Massachusetts General Hospital, Boston, MA USA

**Keywords:** Medical genomics, Rare variants

Correction to: *Nature* 10.1038/s41586-020-2308-7 Published online 27 May 2020

In this Article, author Marquis P. Vawter was missing from the Genome Aggregation Database Consortium list. They are associated with the affiliation: ‘Department of Psychiatry & Human Behavior, University of California Irvine, Irvine, CA, USA’, and contributed to the generation of the primary data incorporated into the gnomAD resource. In addition, in the legend to Fig. 1, ‘ten’ should have been ‘seven’ in the sentence: “**a**, Uniform manifold approximation and projection (UMAP)^46,47^ plot depicting the ancestral diversity of all individuals in gnomAD, using **seven** principal components.” The original Article has been corrected online.

