# Addendum: The mutational constraint spectrum quantified from variation in 141,456 humans

**DOI:** 10.1038/s41586-021-03758-y

**Published:** 2021-08-09

**Authors:** Sanna Gudmundsson, Konrad J. Karczewski, Laurent C. Francioli, Grace Tiao, Beryl B. Cummings, Jessica Alföldi, Qingbo Wang, Ryan L. Collins, Kristen M. Laricchia, Andrea Ganna, Daniel P. Birnbaum, Laura D. Gauthier, Harrison Brand, Matthew Solomonson, Nicholas A. Watts, Daniel Rhodes, Moriel Singer-Berk, Eleina M. England, Eleanor G. Seaby, Jack A. Kosmicki, Raymond K. Walters, Katherine Tashman, Yossi Farjoun, Eric Banks, Timothy Poterba, Arcturus Wang, Cotton Seed, Nicola Whiffin, Jessica X. Chong, Kaitlin E. Samocha, Emma Pierce-Hoffman, Zachary Zappala, Anne H. O’Donnell-Luria, Eric Vallabh Minikel, Ben Weisburd, Monkol Lek, James S. Ware, Christopher Vittal, Irina M. Armean, Louis Bergelson, Kristian Cibulskis, Kristen M. Connolly, Miguel Covarrubias, Stacey Donnelly, Steven Ferriera, Stacey Gabriel, Jeff Gentry, Namrata Gupta, Thibault Jeandet, Diane Kaplan, Christopher Llanwarne, Ruchi Munshi, Sam Novod, Nikelle Petrillo, David Roazen, Valentin Ruano-Rubio, Andrea Saltzman, Molly Schleicher, Jose Soto, Kathleen Tibbetts, Charlotte Tolonen, Gordon Wade, Michael E. Talkowski, Genome Aggregation Database Consortium, Carlos A. Aguilar Salinas, Tariq Ahmad, Christine M. Albert, Diego Ardissino, Gil Atzmon, John Barnard, Laurent Beaugerie, Emelia J. Benjamin, Michael Boehnke, Lori L. Bonnycastle, Erwin P. Bottinger, Donald W. Bowden, Matthew J. Bown, John C. Chambers, Juliana C. Chan, Daniel Chasman, Judy Cho, Mina K. Chung, Bruce Cohen, Adolfo Correa, Dana Dabelea, Mark J. Daly, Dawood Darbar, Ravindranath Duggirala, Josée Dupuis, Patrick T. Ellinor, Roberto Elosua, Jeanette Erdmann, Tõnu Esko, Martti Färkkilä, Jose Florez, Andre Franke, Gad Getz, Benjamin Glaser, Stephen J. Glatt, David Goldstein, Clicerio Gonzalez, Leif Groop, Christopher Haiman, Craig Hanis, Matthew Harms, Mikko Hiltunen, Matti M. Holi, Christina M. Hultman, Mikko Kallela, Jaakko Kaprio, Sekar Kathiresan, Bong-Jo Kim, Young Jin Kim, George Kirov, Jaspal Kooner, Seppo Koskinen, Harlan M. Krumholz, Subra Kugathasan, Soo Heon Kwak, Markku Laakso, Terho Lehtimäki, Ruth J. F. Loos, Steven A. Lubitz, Ronald C. W. Ma, Daniel G. MacArthur, Jaume Marrugat, Kari M. Mattila, Steven McCarroll, Mark I. McCarthy, Dermot McGovern, Ruth McPherson, James B. Meigs, Olle Melander, Andres Metspalu, Benjamin M. Neale, Peter M. Nilsson, Michael C. O’Donovan, Dost Ongur, Lorena Orozco, Michael J. Owen, Colin N. A. Palmer, Aarno Palotie, Kyong Soo Park, Carlos Pato, Ann E. Pulver, Nazneen Rahman, Anne M. Remes, John D. Rioux, Samuli Ripatti, Dan M. Roden, Danish Saleheen, Veikko Salomaa, Nilesh J. Samani, Jeremiah Scharf, Heribert Schunkert, Moore B. Shoemaker, Pamela Sklar, Hilkka Soininen, Harry Sokol, Tim Spector, Patrick F. Sullivan, Jaana Suvisaari, E. Shyong Tai, Yik Ying Teo, Tuomi Tiinamaija, Ming Tsuang, Dan Turner, Teresa Tusie-Luna, Erkki Vartiainen, Marquis P. Vawter, James S. Ware, Hugh Watkins, Rinse K. Weersma, Maija Wessman, James G. Wilson, Ramnik J. Xavier, Benjamin M. Neale, Mark J. Daly, Daniel G. MacArthur

**Affiliations:** 1 Program in Medical and Population Genetics, Broad Institute of MIT and Harvard, Cambridge, MA USA; 2 Analytic and Translational Genetics Unit, Massachusetts General Hospital, Boston, MA USA; 3 Program in Biological and Biomedical Sciences, Harvard Medical School, Boston, MA USA; 4 Program in Bioinformatics and Integrative Genomics, Harvard Medical School, Boston, MA USA; 5 Center for Genomic Medicine, Massachusetts General Hospital, Boston, MA USA; 6 Institute for Molecular Medicine Finland, Helsinki, Finland; 7 Data Sciences Platform, Broad Institute of MIT and Harvard, Cambridge, MA USA; 8 Centre for Translational Bioinformatics, William Harvey Research Institute, Barts and the London School of Medicine and Dentistry, Queen Mary University of London and Barts Health NHS Trust, London, UK; 9 Stanley Center for Psychiatric Research, Broad Institute of MIT and Harvard, Cambridge, MA USA; 10 National Heart & Lung Institute and MRC London Institute of Medical Sciences, Imperial College London, London, UK; 11 Cardiovascular Research Centre, Royal Brompton & Harefield Hospitals NHS Trust, London, UK; 12 Department of Pediatrics, University of Washington, Seattle, WA USA; 13 Wellcome Sanger Institute, Wellcome Genome Campus, Hinxton, Cambridge UK; 14 Vertex Pharmaceuticals Inc, Boston, MA USA; 15 Division of Genetics and Genomics, Boston Children’s Hospital, Boston, MA USA; 16 Department of Pediatrics, Harvard Medical School, Boston, MA USA; 17 Department of Genetics, Yale School of Medicine, New Haven, CT USA; 18 Broad Genomics, Broad Institute of MIT and Harvard, Cambridge, MA USA; 19 Department of Neurology, Harvard Medical School, Boston, MA USA; 150 Present Address: Centre for Population Genomics, Garvan Institute of Medical Research, and UNSW Sydney, Sydney, New South Wales Australia; 151 Present Address: Centre for Population Genomics, Murdoch Children’s Research Institute, Melbourne, Victoria Australia; 20 Unidad de Investigacion de Enfermedades Metabolicas, Instituto Nacional de Ciencias Medicas y Nutricion, Mexico City, Mexico; 21 Peninsula College of Medicine and Dentistry, Exeter, UK; 22 Division of Preventive Medicine, Brigham and Women’s Hospital, Boston, MA USA; 23 Division of Cardiovascular Medicine, Brigham and Women’s Hospital and Harvard Medical School, Boston, MA USA; 24 Department of Cardiology, University Hospital, Parma, Italy; 25 Department of Biology, Faculty of Natural Sciences, University of Haifa, Haifa, Israel; 26 Department of Medicine, Albert Einstein College of Medicine, Bronx, NY USA; 27 Department of Genetics, Albert Einstein College of Medicine, Bronx, NY USA; 28 Department of Quantitative Health Sciences, Lerner Research Institute, Cleveland Clinic, Cleveland, OH USA; 29 Sorbonne Université, APHP, Gastroenterology Department, Saint Antoine Hospital, Paris, France; 30 Framingham Heart Study, National Heart, Lung, & Blood Institute and Boston University, Framingham, MA USA; 31 Department of Medicine, Boston University School of Medicine, Boston, MA USA; 32 Department of Epidemiology, Boston University School of Public Health, Boston, MA USA; 33 Department of Biostatistics, Center for Statistical Genetics, University of Michigan, Ann Arbor, MI USA; 34 National Human Genome Research Institute, National Institutes of Health, Bethesda, MD USA; 35 The Charles Bronfman Institute for Personalized Medicine, Icahn School of Medicine at Mount Sinai, New York, NY USA; 36 Department of Biochemistry, Wake Forest School of Medicine, Winston-Salem, NC USA; 37 Center for Genomics and Personalized Medicine Research, Wake Forest School of Medicine, Winston-Salem, NC USA; 38 Center for Diabetes Research, Wake Forest School of Medicine, Winston-Salem, NC USA; 39 Department of Cardiovascular Sciences and NIHR Leicester Biomedical Research Centre, University of Leicester, Leicester, UK; 40 NIHR Leicester Biomedical Research Centre, Glenfield Hospital, Leicester, UK; 41 Department of Epidemiology and Biostatistics, Imperial College London, London, UK; 42 Department of Cardiology, Ealing Hospital NHS Trust, Southall, UK; 43 Imperial College Healthcare NHS Trust, Imperial College London, London, UK; 44 Department of Medicine and Therapeutics, The Chinese University of Hong Kong, Hong Kong, China; 45 Department of Medicine, Harvard Medical School, Boston, MA USA; 46 Program for Neuropsychiatric Research, McLean Hospital, Belmont, MA USA; 47 Department of Medicine, University of Mississippi Medical Center, Jackson, MI USA; 48 Department of Epidemiology, Colorado School of Public Health, Aurora, CO USA; 49 Department of Medicine and Pharmacology, University of Illinois at Chicago, Chicago, IL USA; 50 Department of Genetics, Texas Biomedical Research Institute, San Antonio, TX USA; 51 Department of Biostatistics, Boston University School of Public Health, Boston, MA USA; 52 Cardiac Arrhythmia Service and Cardiovascular Research Center, Massachusetts General Hospital, Boston, MA USA; 53 Cardiovascular Epidemiology and Genetics, Hospital del Mar Medical Research Institute (IMIM), Barcelona, Catalonia Spain; 54 Centro de Investigación Biomédica en Red Enfermedades Cardiovaculares (CIBERCV), Barcelona, Catalonia Spain; 55 Department of Medicine, Medical School, University of Vic-Central University of Catalonia, Vic, Catalonia Spain; 56 Institute for Cardiogenetics, University of Lübeck, Lübeck, Germany; 57 DZHK (German Research Centre for Cardiovascular Research), partner site Hamburg/Lübeck/Kiel, Lübeck, Germany; 58 University Heart Center Lübeck, Lübeck, Germany; 59 Estonian Genome Center, Institute of Genomics, University of Tartu, Tartu, Estonia; 60 Helsinki University and Helsinki University Hospital, Clinic of Gastroenterology, Helsinki, Finland; 61 Diabetes Unit, Massachusetts General Hospital, Boston, MA USA; 62 Center for Genomic Medicine, Massachusetts General Hospital, Boston, MA USA; 63 Program in Metabolism, Broad Institute of MIT and Harvard, Cambridge, MA USA; 64 Institute of Clinical Molecular Biology (IKMB), Christian-Albrechts-University of Kiel, Kiel, Germany; 65 Bioinformatics Consortium, Massachusetts General Hospital, Boston, MA USA; 66 Cancer Genome Computational Analysis Group, Broad Institute of MIT and Harvard, Cambridge, MA USA; 67 Department of Pathology, Massachusetts General Hospital, Boston, MA USA; 68 Cancer Center, Massachusetts General Hospital, Boston, MA USA; 69 Endocrinology and Metabolism Department, Hadassah-Hebrew University Medical Center, Jerusalem, Israel; 70 Department of Psychiatry and Behavioral Sciences, SUNY Upstate Medical University, Syracuse, NY USA; 71 Institute for Genomic Medicine, Columbia University Medical Center, Hammer Health Sciences, New York, NY USA; 72 Department of Genetics and Development, Columbia University Medical Center, Hammer Health Sciences, New York, NY USA; 73 Centro de Investigacion en Salud Poblacional, Instituto Nacional de Salud Publica, Cuernavaca, Mexico; 74 Genomics, Diabetes and Endocrinology, Lund University, Lund, Sweden; 75 Lund University Diabetes Centre, Malmö, Sweden; 76 Human Genetics Center, University of Texas Health Science Center at Houston, Houston, TX USA; 77 Department of Neurology, Columbia University, New York, NY USA; 78 Institute of Genomic Medicine, Columbia University, New York, NY USA; 79 Institute of Biomedicine, University of Eastern Finland, Kuopio, Finland; 80 Department of Psychiatry, Helsinki University Central Hospital, Lapinlahdentie, Helsinki, Finland; 81 Department of Medical Epidemiology and Biostatistics, Karolinska Institutet, Stockholm, Sweden; 82 Icahn School of Medicine at Mount Sinai, New York, NY USA; 83 Department of Neurology, Helsinki University Central Hospital, Helsinki, Finland; 84 Department of Public Health, Faculty of Medicine, University of Helsinki, Helsinki, Finland; 85 Cardiovascular Disease Initiative and Program in Medical and Population Genetics, Broad Institute of MIT and Harvard, Cambridge, MA USA; 86 Center for Genome Science, Korea National Institute of Health, Chungcheongbuk-do, South Korea; 87 MRC Centre for Neuropsychiatric Genetics & Genomics, Cardiff University School of Medicine, Cardiff, UK; 88 Department of Health, National Institute for Health and Welfare (THL), Helsinki, Finland; 89 Section of Cardiovascular Medicine, Department of Internal Medicine, Yale School of Medicine, New Haven, CT USA; 90 Division of Pediatric Gastroenterology, Emory University School of Medicine, Atlanta, GA USA; 91 Department of Internal Medicine, Seoul National University Hospital, Seoul, South Korea; 92 Institute of Clinical Medicine, The University of Eastern Finland, Kuopio, Finland; 93 Kuopio University Hospital, Kuopio, Finland; 94 Department of Clinical Chemistry, Fimlab Laboratories and Finnish Cardiovascular Research Center-Tampere, Faculty of Medicine and Health Technology, Tampere University, Tampere, Finland; 95 The Mindich Child Health and Development Institute, Icahn School of Medicine at Mount Sinai, New York, NY USA; 96 Li Ka Shing Institute of Health Sciences, The Chinese University of Hong Kong, Hong Kong, China; 97 Hong Kong Institute of Diabetes and Obesity, The Chinese University of Hong Kong, Hong Kong, China; 98 Cardiovascular Research REGICOR Group, Hospital del Mar Medical Research Institute (IMIM), Barcelona, Catalonia Spain; 99 Department of Genetics, Harvard Medical School, Boston, MA USA; 100 Oxford Centre for Diabetes, Endocrinology and Metabolism, University of Oxford, Churchill Hospital, Headington, Oxford UK; 101 Wellcome Centre for Human Genetics, University of Oxford, Oxford, UK; 102 Oxford NIHR Biomedical Research Centre, Oxford University Hospitals NHS Foundation Trust, John Radcliffe Hospital, Oxford, UK; 103 F Widjaja Foundation Inflammatory Bowel and Immunobiology Research Institute, Cedars-Sinai Medical Center, Los Angeles, CA USA; 104 Atherogenomics Laboratory, University of Ottawa Heart Institute, Ottawa, Canada; 105 Division of General Internal Medicine, Massachusetts General Hospital, Boston, MA USA; 106 Department of Clinical Sciences, University Hospital Malmo Clinical Research Center, Lund University, Malmo, Sweden; 107 Department of Clinical Sciences, Lund University, Skane University Hospital, Malmo, Sweden; 108 Instituto Nacional de Medicina Genómica (INMEGEN), Mexico City, Mexico; 109 Medical Research Institute, Ninewells Hospital and Medical School, University of Dundee, Dundee, UK; 110 Department of Molecular Medicine and Biopharmaceutical Sciences, Graduate School of Convergence Science and Technology, Seoul National University, Seoul, South Korea; 111 Department of Psychiatry, Keck School of Medicine at the University of Southern California, Los Angeles, CA USA; 112 Department of Psychiatry and Behavioral Sciences, Johns Hopkins University School of Medicine, Baltimore, MD USA; 113 Division of Genetics and Epidemiology, Institute of Cancer Research, London, UK; 114 Medical Research Center, Oulu University Hospital, Oulu, Finland and Research Unit of Clinical Neuroscience, Neurology, University of Oulu, Oulu, Finland; 115 Research Center, Montreal Heart Institute, Montreal, Quebec Canada; 116 Department of Medicine, Faculty of Medicine, Université de Montréal, Quebec, Canada; 117 Department of Biomedical Informatics, Vanderbilt University Medical Center, Nashville, TN USA; 118 Department of Medicine, Vanderbilt University Medical Center, Nashville, TN USA; 119 Department of Biostatistics and Epidemiology, Perelman School of Medicine at the University of Pennsylvania, Philadelphia, PA USA; 120 Department of Medicine, Perelman School of Medicine at the University of Pennsylvania, Philadelphia, PA USA; 121 Center for Non-Communicable Diseases, Karachi, Pakistan; 122 National Institute for Health and Welfare, Helsinki, Finland; 123 Deutsches Herzzentrum München, Munich, Germany; 124 Technische Universität München, Munich, Germany; 125 Division of Cardiovascular Medicine, Nashville VA Medical Center and Vanderbilt University, School of Medicine, Nashville, TN USA; 126 Department of Psychiatry, Icahn School of Medicine at Mount Sinai, New York, NY USA; 127 Department of Genetics and Genomic Sciences, Icahn School of Medicine at Mount Sinai, New York, NY USA; 128 Institute for Genomics and Multiscale Biology, Icahn School of Medicine at Mount Sinai, New York, NY USA; 129 Institute of Clinical Medicine, Neurology, University of Eastern Finlad, Kuopio, Finland; 130 Department of Twin Research and Genetic Epidemiology, King’s College London, London, UK; 131 Departments of Genetics and Psychiatry, University of North Carolina, Chapel Hill, NC USA; 132 Saw Swee Hock School of Public Health, National University of Singapore, National University Health System, Singapore, Singapore; 133 Department of Medicine, Yong Loo Lin School of Medicine, National University of Singapore, Singapore, Singapore; 134 Duke-NUS Graduate Medical School, Singapore, Singapore; 135 Life Sciences Institute, National University of Singapore, Singapore, Singapore; 136 Department of Statistics and Applied Probability, National University of Singapore, Singapore, Singapore; 137 Folkhälsan Institute of Genetics, Folkhälsan Research Center, Helsinki, Finland; 138 HUCH Abdominal Center, Helsinki University Hospital, Helsinki, Finland; 139 Center for Behavioral Genomics, Department of Psychiatry, University of California, San Diego, CA USA; 140 Institute of Genomic Medicine, University of California, San Diego, CA USA; 141 Juliet Keidan Institute of Pediatric Gastroenterology, Shaare Zedek Medical Center, The Hebrew University of Jerusalem, Jerusalem, Israel; 142 Instituto de Investigaciones Biomédicas UNAM, Mexico City, Mexico; 143 Instituto Nacional de Ciencias Médicas y Nutrición Salvador Zubirán, Mexico City, Mexico; 144 Radcliffe Department of Medicine, University of Oxford, Oxford, UK; 145 Department of Gastroenterology and Hepatology, University of Groningen and University Medical Center Groningen, Groningen, The Netherlands; 146 Department of Physiology and Biophysics, University of Mississippi Medical Center, Jackson, MS USA; 147 Program in Infectious Disease and Mi--crobiome, Broad Institute of MIT and Harvard, Cambridge, MA USA; 148 Center for Computational and Integrative Biology, Massachusetts General Hospital, Boston, MA USA; 149 Department of Psychiatry and Human Behavior, University of California Irvine, Irvine, CA USA

**Keywords:** Medical genomics, Rare variants

Addendum to: *Nature* 10.1038/s41586-020-2308-7 Published online 27 May 2020

This analysis explores the extent of loss-of-function (LoF) tolerance in human disease genes.

Databases of human population genetic variation, such as the Genome Aggregation Database (gnomAD), are generally expected to be depleted for variation with severe effects on health. As such, it is expected that genes that carry highly disruptive changes, predicted (p)LoF variants, in these databases are less likely to be responsible for severe human disease. However, the precise relationship between pLoF tolerance and human disease causation is not well-characterized.

In our Article, we reported a total of 2,636 variants in 1,815 genes that were homozygous in at least one individual and annotated as pLoF after applying both automated filtering and manual curation of both sequencing quality and functional annotation. We labelled these genes as ‘LoF-tolerant’, indicating that total functional loss of these genes appears to be compatible with life. This does not exclude the involvement of these genes in diseases compatible with presence in individuals in gnomAD^1^. Neither the ‘LoF Transcript Effect Estimator’ (LOFTEE) nor manual curation took previous gene–phenotype associations into account, as this would create a bias that affects downstream analyses and also may result in the spurious exclusion of true LoF-tolerant genes owing to previous false-positive reported associations with disease. This unbiased approach is appropriate for permitting downstream analyses, but it means that the enrichment of pLoF artefacts will remain higher in genes for which genetic disruption is genuinely associated with severe disease.

Prompted by comments on our original Article, we explored the degree to which our LoF-tolerant list includes genes associated with disease by manually curating the 158 genes (with 217 pLoF variants) on the LoF-tolerant list associated with autosomal recessive and X-linked traits in ‘Online Mendelian Inheritance in Man’ (OMIM) by an additional biocurator^1^.

Of these genes, 71% (*n* = 112) are associated with phenotypes that are likely to be found in gnomAD, on the basis of gnomAD inclusion criteria. These are phenotypes such as infertility, hearing or visual impairment, benign or mild metabolic or haematological phenotypes, expected at similar frequency as the general population (95 phenotypes) and, to a lesser extent, traits that are likely to be depleted from gnomAD, but for which someone with the condition may participate in a common disease study (17 phenotypes). We observed an overrepresentation of traits that are likely to be found (60% versus 33%) and an underrepresentation of traits that are not expected to be found (29% versus 53%) in gnomAD (early-onset severe or lethal rare disease that generally would restrict participation in genetic studies) versus a control set of 100 random selected autosomal recessive and X-linked OMIM traits (*P* = 3.0 × 10^−5^, Fisher’s exact test) (Fig. 1a). We performed a thorough literature review of the 46 phenotypes that were initially not expected to be found in gnomAD, which revealed that 35% (16 out of 46) can be explained by evidence of mechanism of disease not being LoF (*n* = 2), variable expressivity (*n* = 5) or penetrance (*n* = 3), phenotype being responsive to treatment (*n* = 4) and onset after age of the individual in gnomAD (*n* = 2) (Fig. 1b, blue).

**Fig. 1:**
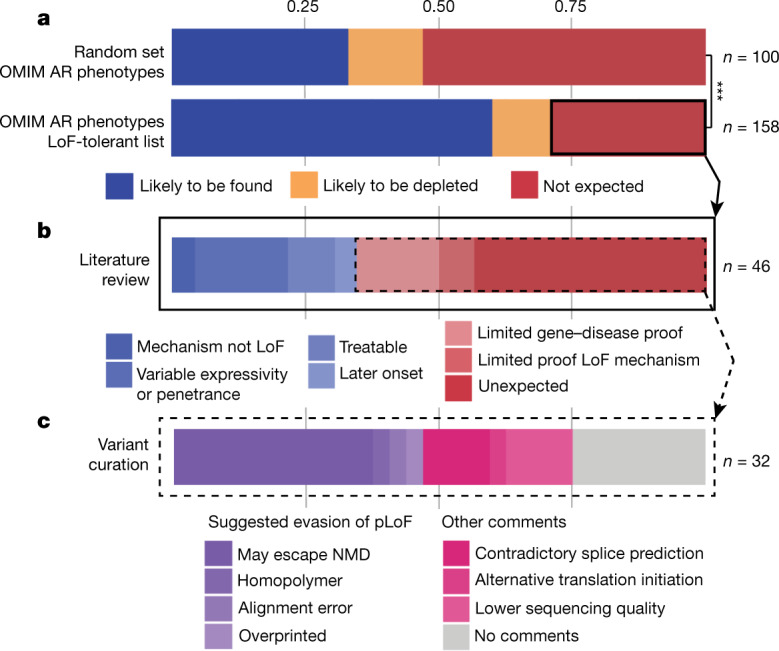
Assessment of pLoF variants in LoF-tolerant genes associated with autosomal recessive and X-linked phenotypes in OMIM. **a**, Autosomal recessive and X-linked (AR) OMIM phenotypes: likely to be found (blue), likely to be depleted (yellow) or not expected (red) to be found in gnomAD, for the 158 phenotypes associated with LoF-tolerant genes in gnomAD and a set of 100 randomly selected AR and X-linked OMIM traits. ***P = 3.0 × 10^−5^, Fisher’s exact test. **b**, Extended literature review of the 46 out of 158 OMIM phenotypes not expected to be found in gnomAD. **c**, Extended variant curation of 32 pLoF variants in 30 LoF-tolerant genes beyond criteria presented in our original Article revealed pLoF with suggested evasion of pLoF (purple), and pLoF with no conclusive (pink) or no evidence (grey) contradicting pLoF in these genes. NMD, nonsense-mediated decay. Further details are provided in Supplementary Table 1.

In contrast to what is expected to be found in gnomAD, 32 pLoF variants are in 30 genes for which homozygous LoF has been associated with severe or lethal phenotypes in OMIM. However, 10 of these 30 genes had a limited number of cases reported (*n* = 7) or no reported biallelic LoF variants in humans (*n* = 3) (Fig. 1b, light red) and only 5 genes meet current ClinGen standards for a known LoF mechanism^2^. We evaluated the 32 variants by applying more stringent criteria, and identified several cases in which a variety of mechanisms may result in an evasion of true loss of gene function. For 15 variants, we found evidence that disputed our previous prediction (Fig. 1c, purple), including variants that are suspected to escape nonsense-mediated decay but that did not meet the criteria for rescue applied in our original Article (*n* = 12), one variant that was within a small homopolymer and thus is more likely to represent a sequencing error, one alignment error, and one variant that is in an overprinted transcript and is more probably a synonymous variant in the most biologically relevant transcript. For the 17 variants for which we cannot identify conclusive (*n* = 9) (Fig. 1c, pink) or any (*n* = 8) (Fig. 1c, grey) evidence for evasion of pLoF, there are several explanations that even our stringent curation cannot confidently exclude: for example, sample swaps, a variety of residual sequencing and annotation artefact classes, the presence of an individual in gnomAD who does actually have the expected phenotype, or simply variable expressivity, late age of onset or reduced penetrance of the disease phenotype itself. Further details regarding variant curation are are available in Supplementary Table 1 and from https://gnomad.broadinstitute.org/downloads, or the curation data can be viewed at the respective gene page at https://gnomad.broadinstitute.org.

In summary, this result emphasizes the well-established need for extremely careful curation of any pLoF variant observed in a population database such as gnomAD, especially for genes for which such variants are expected to be deleterious. The variants curated here are found at low frequency and are enriched for both sequencing and annotation errors^3,4^. This enrichment is expected to be even larger in genes for which inactivation is associated with severe disease, because sequencing and annotation artefacts are distributed approximately uniformly across the genome, whereas true LoF variation is depleted in genes in which it results in a more detrimental effect. Although the pLoF variants found in the gnomAD dataset have been subjected to thorough quality control, any filtration process other than comprehensive experimental validation is insufficient to remove all artefacts.

In conclusion, population databases such as gnomAD are a powerful source of information when predicting human tolerance towards gene disruption. The list of LoF-tolerant genes identified in gnomAD is a useful class for downstream analysis that appears to largely comprise genes for which true homozygous disruption does not cause severe early-onset disease.

Authors S.G. and M.S.-B. carried out the analysis described in this Addendum. K.J.K., A.O.-L. and D.G.M. contributed to the experimental design, and A.O.-L. and D.G.M. supervised the work. S.G., M.S.-B., K.J.K., A.O.-L. and D.G.M. wrote the Addendum. A.O.-L. and D.G.M. contributed equally to this work.

We thank C. Arnoult, P. Ray and N. Thierry-Mieg for presenting the opportunity to further clarify the term LoF tolerance.

**Supplementary Information** is available in the online version of this Amendment.

## Supplementary information

Supplementary Table Homozygous pLoF variants in genes associated with autosomal recessive and X-linked phenotypes in OMIM.
