# Supplementary material for: The mutational constraint spectrum quantified from variation in 141,456 humans
Source: Nature. 2020 May 27;581(7809):434–43. doi: 10.1038/s41586-020-2308-7 (PMC7334197; doi:10.1038/s41586-020-2308-7)
Supplement: Supplementary file 3 — Reviewer reports and authors' response from the peer review of this Article at Nature. [file 41586_2020_2308_MOESM3_ESM.pdf]

natureresearch

Peer Review File

Manuscript Title: The mutational constraint spectrum quantified from variation in 141,456 humans

Editorial Notes:

Redactions – Third Party Material

Parts of this Peer Review File have been redacted as indicated to remove third-party material.

Reviewer Comments & Author Rebuttals

Reviewer Reports on the Initial Version:

Referee #1 (Remarks to the Author):

In this manuscript, Karczewski and colleagues describe the gnomAD resource -- aggregate variant calls derived from ~125K human exomes and ~15K human genomes that are already widely used by the research community. The manuscript focuses on predicted loss-of-function (pLOF) variants in particular. The paper is a follow up to the same groups' ExAC resource, published a few years ago, with the overall cohort approximately doubling in size and with markedly better representation of human populations around the world. As general statements, the work appears to be well done from a technical perspective, the analyses thoughtfully conducted, and the material clearly presented. It's obviously been an incredible amount of work to pull these data together, to reanalyze them in a coordinate fashion, and to serve them up to the community in a useful format.

That praise notwithstanding, I do have some high-level as well as more specific concerns.

Major comments

1. The relatively narrow focus on pLOF variation results in some real missed opportunities here. Virtually nothing is said about missense variation, non-canonical splice site variation, or constraint in the non-exome 98% of the genome, for which there are a respectable ~15K genomes available for analysis. My inference is that at least some of these analyses are being slated for other manuscripts, but to be frank I would have preferred an at least modestly more expanded scope. The consequence of this relatively narrow focus on pLOF variants is that the emphasized novelty here, apart from the substantially larger N (as well as what seems to be improvements in methodology throughout, which are not really emphasized), lies with the shift from pLI scores (a continuous metric, but one directed at predicting haploinsufficiency, a binary concept) to LOEUF scores (a continuous metric that reflects the upper bound on a confidence interval for the observed/expected ratio of pLOF variants), together with some of the specific analyses that are performed. This is certainly progress, but it leaves something to be desired if only because feels like a lot is being left on the table in terms of all of the things that one might imagine doing with these data that have nothing to do with pLOF variants.

2. Although the scope could be endless (and although I would love to see at least a bit on the most extremely constrained noncoding regions), the manuscript could be particularly strengthened by adding more content around missense variants in particular. Perhaps I should expect it from past papers, but I am quite surprised by EDF 6f-g in particular and suggest they be moved to the main figures. In particular, the fact that there are so few genes with substantial constraint for missense variants remains surprising to me. Given the considerably higher saturation of missense variants in gnomAD as compared to ExAC, can the authors consider adding regional missense constraint

analyses ala the Samocha et al. 2017 paper?

3. While I'm on those panels (EDF 6f-g), it would be helpful if the extremes, particularly on the excess observed vs. expected, points in all three panels could be labeled and ideally discussed. Are there any genes for which significantly more synonymous, missense or pLoF mutations are observed than were expected? Are these technical artifacts or biologically meaningful outliers?

4. The first two paragraphs, although well written, feel a bit misleading. They set up a strawman around the concept that breaking parts of a system can be useful for understanding it, but then mention Mendelian genetics almost as an afterthought. Understanding human biology through LoF mutations and their consequences has been the mainstay of human genetics for nearly a century. What is being done here builds directly on that foundation, rather than being consequent to a motivation drawn from the engineering field or from our inability to edit LoF mutations into humans. Describing this undertaking as the inverse of classical human genetics (i.e. genotype first, rather than phenotype first; looking for the absence of pLOF variants rather than their presence) would be a more honest representation of the field-specific context into which this work falls.

5. A few things about the LOEUF metric remain unclear to me:

a. No justification is provided for using the upper bound of the confidence interval rather than simply using the o/e ratio itself. I can understand the argument that might be made, but I can also imagine counterarguments. In any case, thought process by which this decision was made should be laid out. The confidence intervals seem to be quite wide? (I'm inferring this from the fact that the median o/e of 48% jumps to a median LOEFF of 0.962).
b. Unless I missed it, you show histograms of o/e and LOEUF scores, but no comparison of the metrics to one another. Related to point (a) above, it would be helpful to present scatter plots of o/e vs. LOEUF, and also o/e vs. pLI and LOEUF vs. pLI, to provide the reader with a better view on the extent to which they differ?
c. Many "summary plots" are presented, but I feel like I still lack a raw sense of how the wide the confidence intervals are for the o/e ratios that give rise to the LOEUF scores, as well as how these confidence intervals vary as a function of the o/e ratios. I suggest that you add a figure that shows the stacked means and confidence intervals for each gene, sorted by means (or a random subset of genes if the entire set can't be presented). To phrase it another way – a plot where every gene has its own line, and the genes are ranked by o/e, and the o/e and confidence intervals for each gene are shown.

6. What are the considerations involved in estimating selection coefficients from the observed vs. expected data? Rather than deciles or this upper bound o/e value, it would be very helpful to have these estimates, particularly in light of arguments made in this recent paper from Fuller et al. (https://www.nature.com/articles/s41588-019-0383-1) which I urge the authors to cite and discuss in relation to the shift from pLI to LOEUF (and ideally to motivate just going ahead and estimating the strength of selection on heterozygotes on the basis of the updated data in gnomAD).

7. There are many instances where the authors provide highly significant p-values but no corresponding information about the corresponding difference or effect size. As the authors well know, miniscule effects can have large p-values. I noticed this in particular in the paragraph beginning on line 284 (Biological properties…). However, if the authors could review the full paper for similar instances and provide corresponding effect sizes or fold differences or whatever is appropriate along with the p-values, that would be helpful.

8. My inference throughout is that when the authors are calculating things such as the proportion of observed variants out of all possible variants, they are including all possible substitutions (3 per site). However, it would be helpful to include at least a bit of information on the extent of "site

saturation". For example, when you say 17.2M and 262M variants, is this approaching ~50% of coding nucleotides and 10% of all nucleotides represented in the human genome, at which at least one of three possible single nucleotide changes is observed? Although the primary focus could/should remain w/ keeping all possible substitutions as the denominator, it would be helpful to know the per-site summary statistics as well. This is also relevant to the summary statistics provided at lines 166-169.

9. Variant calling & indels feel a bit swept under the rug, at least in the main text. I recognize that this is covered in the supplementary figures and methods (and I'm not asking for anything new to be done here; generally convinced), but it would be helpful to have at least a paragraph summarizing the key take-homes from EDFs 2-3.

10. It's not clear how the statement at lines 410-412, that 30% of coding genes are insufficiently powered for detection, is justified. I infer that you are using a cutoff of 10 pLOF mutations expected as the definition of sufficiently powered, but where does this come from? On a related point, at line 230, you state that the increased N from ExAC to gnomAD increases this proportion from 63% to 72%. The relatively marginal increase from doubling the N leads one to wonder – what population sizes are required to get this even higher? I guess my broader point is that a more formal analysis of power as a function of population size, even if you have to project a bit based on the existing data, would be very helpful (and justify the cutoff of 10 or whatever alternative is chosen). What population size would it take to get to 95% of genes with at least 10 pLOF mutations expected?

Additional comments:

11. The claim is made towards the end of the introduction that the metric "improves rare disease diagnosis" – is this actually done in the paper? It seems like this should be rephrased to be more in line with what is actually described in the manuscript (unless I missed something, which is possible).

12. For LOEUF, is is the 95% CI that is used? I assume so, but it should be stated.

13. More could be said, or at least a citation added, for the data points in Fig. 2E that might be explained by hypo-methylated CpGs giving rise to a lower rate of % observed? On a related point, in EDF 4b, is it possible to partition CpG sites on those that are broadly methylated vs. those that are not? (i.e. is it possible that you are completely saturating those that are consistently methylated?)

14. It would be helpful if the authors review the manuscript specifically to look gene lists that might be useful to readers, and to actually provide those as supplementary tables. For example, at line 216, a set of 1,752 genes that are likely to be tolerant to biallelic activation are referenced but a corresponding file/table not provided.

15. Line 161 – "all possible synonymous methylated CpG variants" – odd phrasing – do you mean all possible CG>TG changes? Or are you subsetting to sites that are consistently methylated? More clarity here would be helpful.

16. Fig 3d – the notion of "cell essential" is a little strange. What cell type? Are these genes that are consistently essential across CRISPR screens? More clarity on what these are would be good to include rather than requiring the reader to look at the reference.

17. In Fig. 5c, there are few enough points above the cutoff that it would be better if you could find a way to just label them all. But on a related point, I think the corresponding section of the discussion is too bold. Unless I'm missing something, the last sentence of the results feels too bold. The results primarily follow from a few phenotypes, all related to brain function, and possibly

conflated with one another. It would be better to restrict the claim to this subset of phenotypes rather than 'many heritable polygenic diseases and traits'.

18. Less a request than a suggestion, but I would love to see more discussion or at least some brief quantification of the proportion of top-decile LOEUF genes (i.e. most constrained; n = 1,920) in terms of what % are associated with a human phenotype, what % have an assigned function, what % have no known function, etc.

Referee #2 (Remarks to the Author):

Summary

In the manuscript entitled "Variation across 141,456 human genomes and exomes reveals the spectrum of loss-of-function tolerance across human protein-coding genes", Karczewski and colleagues describe the Genome Aggregation Database (gnomAD). This is a substantial augmentation to the Exome Aggregation (ExAC) database, including additional exome data as well as adding 15,708 genomes. The authors describe the data contained within this resource, the quality control measures, and highlight a particular variant class, predicted loss of function (pLOF). They develop a metric termed LOEUF that is used to categorize genes with respect to their tolerance for loss of function variation. They then provide examples demonstrating how this information can be used in disease gene research.

High level comments

The impact that the gnomAD and ExAC resource has had on both the research and clinical genomics communities cannot be overstated. The dedication this group has to making this data available to the research and clinical communities is exemplary. This project has been a flagship for demonstrating how large-scale sequencing coupled with summary data release can be transformative for the field.

With respect to this manuscript, the authors were wise to focus on a narrow aspect of this data and demonstrate how this specific variant type (pLOF) can contributes to disease gene research. The manuscript walks through how the LOEUF deciles are generated, and provides numerous analysis of how this can be used. However, the manuscript fails to connect this with specific biology that resonates in an impactful way. There are no specific examples pulled from the categories to make the data more meaningful. For example, when looking at figure 3, there is so much more information I want to know. This includes identifying specific genes that fall into expected LOEUF deciles, but also discussing the unexpected events. What are the haploinsufficient genes that are in the lower LOEUF deciles? Does any of this information confirm or refute known orthogonal data about genes in these bins? Perhaps even just noting where the ACMG59 fall in these deciles, or within these analysis would be useful. Though I understand that the ACMG59 may not be under the strongest constraint, a data set such as this could help guide the community about the best way to utilize this data.

I do think there are some other missed opportunities for educating users around some issues of this analysis. While this manuscript focuses on global analyses, it is important to remember that many users will look at individual genes, and understanding how individual genes may be impacted by analytical decisions is important for ensuring the data are used correctly.

More detailed comments

1. pLOF set definition: The authors are very well versed in the types of annotation errors that can lead to false variant calls, and understand the pLOFs are likely to be enriched in annotation errors. While I think the authors do a very commendable job of cleaning this dataset, I would urge the authors

to review a recent manuscript from Tuladhar et al., 2019 (http://dx.doi.org/10.1101/583138) which is focused on analyzing putative knockout alleles from CRISPR lines. In this manuscript, they find that 46% of edited lines marketed as knockouts, due to the presence of an indel, are not actually knockouts, but that some product is produced via other escape mechanisms. While I don't think the authors need to any additional experimental work for this manuscript, this reference, plus additional caution that individual pLOF events need to be followed up with functional assays to confirm actual LOF is warranted. Particularly because the authors go to so much effort to produce a high quality dataset, users need to be reminded that while collectively, the data are of high quality, individual events should be verified. It might be interesting to run LOFTEE on the dataset in the Tuladhar manuscript to see how many of the 'escapees' are flagged by LOFTEE.
2. Variant identification: There are two points to be raised here. The authors do a great job of trying to eliminate false positives, even at the expense of potentially losing true positives. However, the authors don't mention the impact of false negatives. For example, STRC has very poor coverage of exons 19-25 due to the presence of a paralogous sequence in the genome, complicating alignment and leading to low or no coverage. What percentage of the genome, particularly the clinically relevant genome, falls into this category? Does gnomAD do better/the same/worse in difficult regions such as those as described in Mandelker et al., 2016 (https://doi.org/10.1038/gim.2016.58)? Does the genome data help in some of these cases? Also, do the authors have any comments on the impact of genome data vs. exome data in terms of variant identification? What are the technical advantages/disadvantages of genome vs. exome? For example, when looking at PKD2 in the gnomAD browser, it looks as if genome data rescues poor coverage of the first exon as seen in exome data. How often does this sort of thing occur?
3. More details on specific genes: There are a few places that summary data is provided, and what I really want are the details. For example, on line 216, there is a statement about 1752 genes that are likely intolerant to biallelic activation- what are these genes? I expected a supplementary table, but there is none (though apologies if I missed this). I had the same reaction to Supplementary table 15- I would love to see a giant table, one row for each gene with the classification (column headers in this table) and LOEUF decile. This would really let me dig into some interesting stuff. Are there any surprising genes in these deciles (lines 245-249). In fact, some of the disease genes that unexpectedly fall into the lower LOEUF categories would be some of the first ones I'd want to test for an escape mechanism leading to expression despite the prediction of LOF.
4. Figures and data in general: I found the availability of numbers and consistent metrics that support data figures was inconsistent. Ideally, these numbers would be contained within the figures or at least the figure legend (for example, every time there is a correlation, I'd like to see the correlation values in the figure or legend, not just in the text). I found myself having to go back and forth between the text and figures a lot, and occasionally I thought I found inconsistencies, though I'm not always sure if the data are inconsistent, or I'm just having trouble matching the text to the appropriate figure. For example, line 225-226 notes 'the variation in the number of synonymous variants observed is accurately captured (r2=0.958)'. The data in extended figure 6f, which states r=0.9791- so consistent use of either r or r2 would be appreciated. Even just ensuring that numbers are in figure legends, if not the figure, is useful for more easily interpreting the figures. Please review that legends and colors are clear. For example, what do the colors in figure 5c mean? I spent an embarrassing amount of time looking for the 'circles' in extended Data Figure 1, to realized the rounded corner squares were what I should look for.
5. Assembly information: While I am sympathetic to the needs to use the woefully old GRCh37 assembly (I myself had to do this for a recent manuscript) it is useful to explain to users why this very old reference is used (page 3 of the supplement) and what the shortcomings are. While I firmly believe the reference assembly version used will not impact the overall findings of this paper, it may impact the information at any given locus. For example, a big focus of the GRCh38 update was to improve clinically relevant genes (for example, adding in 3 missing coding exons of Shank3, and adding a new paralog of KCNE1 which means many of the variants called in GRCh37 may actually be paralogous sequence variants) and users should understand these caveats. This also impacts the pLOF variant curation, as regions known to be different between GRCh37 and

GRCh38, as well as known assembly problems from the GRC would likely be useful in this analysis (supplemental page 32). Lastly, please

use consistent nomenclature when referring to the assembly. The official assembly name is GRCh37, but there are various distribution 'flavors'. And while I weep that this is the case, it is important to note the data source (hg19 specifically implies data from UCSC for example) so that these slight variances can be taken into account.
6. Variant annotation and constraint modeling: The supplement sections read as if they are stand alone sections. I understand why this happens and in many cases this is not a problem, but I had some trouble understanding where variant annotation, and some of the gene level metrics stop and the constraint modeling starts. As I was reading the main text, I was curious as to how regions of low coverage (and thus potential false negatives) impacted the LOEUF and gene level metrics. Does something look more intolerant to LOF because no variants are called because of low coverage? The constraint modeling section of the supplement explains how coverage is taken into account, but the variant annotation section, which has some information on gene level metrics, does not. My general assumption is that these two sections really work together in a way that is not entirely clear to me from reading the text, but perhaps I am wrong about that. It would be nice to clarify some of these metrics with some examples- i.e what calculating the data looks like on a well covered gene versus a genes like STRC, SMN1 and IKBKG (thank you for the lovely browser that made looking these examples up relatively straightforward!). Additionally, Figure 2D highlights that 30% of coding genes in the genome are still underpowered to detect constraint-how many ClinVar or ACMG59 genes fall into this category?
7. Exon level metrics: Have the authors considered calculating these metrics at the exon level rather than the gene level? Would this provide even more fine grained information? If a gene only has a small number of exons under constraint, could it end up in one of the higher LOEUF deciles depending on gene size? Could this potentially improve variant interpretation? Or would doing this require significantly more samples? There would likely be utility in doing this at the exon level if statistically achievable.

Referee #3 (Remarks to the Author):

In their manuscript entitled 'Variation across 141,456 human exomes and genomes reveals the spectrum of loss-of-function intolerance across human protein-coding genes' Karczewski et al. present the largest human exome/genome dataset published to date.
This is without a doubt a very valuable resource to the field of human genetics/genomics; clinical researchers, diagnostics, etc. Using a set of >440k high confidence pLoF variants, i.e. a set that is more than double the size of ExAC and by applying an improved model (utilizing methylation-base-pair level coverage correction and LOFTEE) they classified the level of LoF intolerance of all protein coding genes.
This expands beyond the use of ExAC for many aspects, which increases the usability to novel reads/users, e.g.:
- More than double sized dataset
- More populations represented
- Exomes plus genomes included
- Options to use dataset with or without certain sub-cohorts (e.g. non-cancer cohorts)
- Isoform refinement
The power of this dataset is confirmed by: a) constraint metric correlated with biological relevance (PPI; gene expression; disease association); b) the constraint metrics reflect model animal and cellular KO phenotypes; c) constraint can assist disease gene finding (ratio 15 higher likelihood for de novo mutations in developmental disease genes in LOEUF decile).

There are however several major aspects that require refinement, and several new aspects could additionally boost the scientific value, add novel insights or increase the usability even more.

1.) It would be interesting to the readers, in which aspects the authors improved over previous work: MacArthur et al. Science (2012) (DOI: 10.1126/science.1215040); Lek et al. Nature (2016)(DOI: 10.1038/nature19057); and which of the previous conclusions may have been falsified since then. It would be important to stress the novelty aspects of the current work (also to justify publication in this highest ranking journal).
2.) The authors should discuss their findings in light of the recent set of work on 'Genetic paradox explained by nonsense';https://www.nature.com/articles/d41586-019-00823-5
3.) Add a paragraph how many LoF allele human individuals carry on average, per population per frequency range
4.) The author should consider flagging genes for which the majority of pLoF variants appear in a) smaller allelic fractions indication somatic/mosaic state; b) in >average aged individuals. Both would be very indicative for 'drivers of clonal hematopoeisis'; and may prevent false interpretations of pLoF in genes like DNMT3A, ASXL1, TET2 (which in germline may very well cause severe developmental diseases caused by AD mutations) as well as flagging up novel genes with a similar mechanism and biology.
5.) Can the authors describe for which genes/exons the WGS vs WES data improve sensitivity ('dark areas' of exomes)?
6.) Can the authors provide data on compound heterozygous state of pLoF variants in individuals? This would be very informative for a) adding sensitivity that gene that can/cannot tolerate complete Kos; b) show alleles for which frameshifting variants are rescued by other frameshifting in cis in order to restore the reading-frame.
7.) Next to the CNV/SV dataset in preparation (Collins et al.); have the authors compared LOEFF decile genes for overlap with CNV morbidity map (Eichler lab; Cooper et al and Coe et al.); and HI scores by the Hurles lab?
8.) It would be very interesting to understand whether there are genes that are exclusive or enriched for certain types of pLoF. E.g. are there genes that show stop-gains only but no frameshifts or essential splice site pLoF?
9.) Are there any specific pLoF alleles that are significantly enriched in certain populations? E.g. are there any population specific PCSK9-like alleles?
10.) How many isoform specific effects are (not) re-solved by transferring from hg19 to hg38?
11.) The authors cross-reference several other manuscript that are under preparation which remain a bit difficult to judge (as not available in peer-reviewed versions yet), but the released preprints are in line with all claims made here.

Minor issues that may further improve the manuscript:
1.) Line 56: "model of human mutation" isn't this rather "mutation rate"
2.) Line 91: Mention somatic events (and differences in tissues source) as a source of 'false positive germline events'
3.) Line 161: Please add an explanation and citation to the synonymous methylated CpG variants – as the most mutable site of the human genome.
4.) Line 169 (and ext. fig 4): The authors should be able to model the amount of exomes/genomes required to robustly reach saturation across all mutational contexts.
5.) Lines 237-238: Could the author define how much the refined model and the increased sample size to the improved power?
6.) Line 475: change "sex aneuploid" to "sex chromosome aneuploid".
7.) Figure 6b: define "mu" in legend.
8.) Supplement: page 4; why was coverage capped at 100x, and are there any adverse effects expected for capping?
9.) Supplement: page 46: some references are not formatted correctly at first citation (Hamdan; Lelieveld).

Referee #4 (Remarks to the Author):

In, "Variation across 141,456 human exomes and genomes reveals the spectrum of loss-of-function intolerance across human protein-coding genes", Karczewski et al. describe a compilation of variants in exome and genome sequence data from over 100,000 individuals assembled from a variety of projects. They focus on predicted loss-of-function (pLOF) variants inferred to eliminate protein production, and describe pipelines to effectively remove erroneous pLOF variants. They then quantify the extent of observed pLOF variation across populations and genes, asses the relationship between pLOF and transcript expression, and define pLOF gene-level tolerance scores for application in human disease genetics.

In general, this work is of high technical quality (a few minor comments are provided below). Further, the authors are to be commended for their efforts to not only make the data public and usable but also to publish software to generate/parse/filter/etc. GnomAD/ExAC has been a highly impactful resource and this iteration is likely to continue in that regard.

However, my high-level opinion is that while the underlying resource is impressive, this manuscript is a narrow one documenting only incremental advances over previous work. The novelty here is largely due to the increased sample size and in refinements to the methods, e.g., the machine learning approaches to filter variants and the model to infer mutability, but the key concepts and conclusions have been previously published. For example, a key message in this paper, i.e., that mutational tolerance scores usefully separate genes according to phenotypic relevance, is similar to that of Petrovski et al., published in 2013. The distributional shifts presented here in Figures 3a,c,d and 5a, are similar to those shown in Petrovski et al. Figures 2 and 3; in fact, Petrovski et al. used nearly identical types of genes to make the same point (i.e., haploinsufficient, mouse-lethal, OMIM-dominant, OMIM-recessive, and neurodevelopmental-disorder genes). This manuscript is part of a large group of studies that use related methods and lead to similar conclusions about the inference of selective tolerance as a means to identify pathogenic variation (non-comprehensive examples beyond Petrovski et al. include Fu et al. 2013, Samocha et al. 2014, and Gussow et al. 2017).

Thus, the difference between this and previous work is of degree not kind. Towards that end, this analysis does not systematically and precisely measure improvement over previous work, nor is there a systematic delineation of the effects of the various sources of improvement described. For example, while sample size is analyzed in relation to variant saturation, no comparison of LOEUF gene group separation efficiencies (e.g., haploinsufficient, essential, ID/DD, etc) at various sample sizes is demonstrated. Similarly, the variant filtering and mutability models developed here are not contrasted with other models provided the same input data (e.g., RVIS on the same set of pLOF variants), nor are the effects of the differing refinements described here measured as isolated components (e.g., LOEUF on VQSR vs machine-learning-filtered variants or a simple mutability model vs a CpG/methylation/etc-defined model). While I find it highly likely that the results described here are non-trivially more powerful for separating genes known to be relevant to phenotype from those that are not, the improvements are likely to be modest; the more important, pragmatic effect on gene discovery per se is likely to be even smaller given that there is not a strict monotonic correlation between the distributional separations benchmarked here and novel disease gene prioritization effectiveness.

Other key results, such as those related to the contribution of errors to pLOF variants and the relationship between nonsense variants and expression are also conceptually similar to previously published studies, including some by many of the authors here (e.g., MacArthur et al. 2012, Bartha et al. 2015, Rivas et al. 2015, Balasubramanian et al. 2017, Ganna et al. 2018). The preexisting literature on de novo variation in ID/DD, another highlighted result in this manuscript, is too extensive to concisely summarize or cite here, but it is safe to say that the key results here (e.g., Figure 5a) have already been seen in numerous studies that use related approaches and

similar data.

I am not arguing that this manuscript offers nothing distinctive relative to the other cited manuscripts (and the other uncited manuscripts like them). Indeed, I find it likely that there are benefits to the increased sample size and methodological refinements described here. However, these differences are not systematically and precisely quantified, and even if they were I do not believe they would be conceptually or pragmatically large.

There are also some key details and points outsourced to accompanying manuscripts cited as being in preparation, including Cummings et al., Collins et al., Minikel et al., and Whiffin et al., suggesting result overlap that further undermines uniqueness and novelty here. While not cited as such, it appears that these manuscripts are available on Biorxiv (in my opinion, "in preparation" or "data not shown" citations are intrinsically inhibitory to meaningful review and should not be used). After reading these related Biorxiv documents, it is clear that these manuscripts as a group overlap extensively with one another, even beyond the fact that they are all derived from the same underlying genome/exome data. Consider the following (non-comprehensive) examples:

1. LOFTEE is a core method in this manuscript, Cummings et al, and Minikel et al., being used to provide the refined data product (collections of error-depleted pLOF variants) that drives key conclusions across all three manuscripts.

2. Much of the text in Cummings et al. is thematically highly consistent with key results in this manuscript, namely expression levels and distribution in relation to pLOF variation, both real and erroneous. Note, for example, content overlap between Cummings Figure 3 and Karczewski 4b-c and overlap between Cummings Figure 4 and Karczewski 2a.

3. Figure 1 from Minikel et al. is similar to Figure 2 in this manuscript, drawing from the same data and presenting very similar results (e.g., compare Minikel 1c with Karczewski 2c-d). Minikel Figure 1 furthermore appears to be very similar to Extended Figure 5 f-h in this manuscript; all these panels are scatter plots showing observed and expected counts of variants, subset by the same variant types using the same coloring scheme, and whose key conclusion is to indicate gene or transcript-level constraint differences on different categories of variation.

4. Collins Figure 6b and Karczewski 3b both appear to use the same data and lead to similar results, namely the correlation between rates of structural variant observation and constraint on pLOF SNVs.

While these examples of overlap are not plainly duplicative of one another, they tend to provide only mildly different perspectives on the same data and ultimately lead to similar high-level conclusions. In general, there are extensive redundancies across these five manuscripts, including: shared raw, intermediate, and endpoint datasets; shared methods for variant calling and filtration; similar individual results and figures; and shared high-level conclusions.

Thus, while I understand that "lump/split" decisions for manuscripts stemming from large team-driven genomic projects can be challenging, it is my opinion that the split decisions in this case resulted in a too thin manuscript that provides only incremental impact relative to both previously published and concurrently submitted papers. However, I find it likely that a more comprehensive manuscript that combines key points here with those from the companion manuscripts would be both more reader-friendly and more impactful. It could benefit from elimination of the redundancies and better highlighting of those results which are truly new. It would also provide a more cohesive description of GnomAD, the conclusions one can derive from it, and the impact it can have as a resource.

Minor technical comments:

Additional details on the "established gene lists" that drive key results are needed. While a github link is provided, precise descriptions of how they were defined need to be in the manuscript or supplement, along with a discussion about how their ascertainment may influence the correlations and trends observed. This is particularly true to the extent that there are any manual curation steps and to the extent that there may exist implicit or explicit circularities. If, for example, data from a previous generation of ExAC were used to define a given list of genes, then the results presented here might be at least partially tautological. On a related note, who performed these curations and to what extent did they also perform the analyses presented here? I do not doubt the general veracity of these results. However, to the extent that this manuscript is refining methods/data and not providing conceptually new approaches, precisely estimating the actual magnitude of individual refinements is particularly important; thus, any relevant biases in the use of these gene lists as a measure of performance should be removed or controlled for. Ideally, gene lists defined and curated by an independent group and in the absence of ExAC data would be used as validation (e.g., those used in Petrovski et al., which predate these analyses and, I believe, the existence of ExAC as a public resource).

Similar question relates to the process by which OMIM genes were defined as being discovered from WES/WGS vs linkage. Was this work done manually? How does it compare to other efforts (if any)? What about cases in which a combination of both linkage and WES/WGS were used? As per above, the effects of circularity are relevant here given the fact that ExAC has explicitly (e.g., by contributing to variant filtration) and implicitly (e.g., via use of intolerance scores in VUS evaluation) helped to identify some of the WES/WGS-discovered genes; this will likely be difficult to account for but clearly may confound the interpretation here.

While I have not attempted to run the software or thoroughly check the documentation, I have little doubt about the quality and utility of the software; such work is, in fact, one area where this group has a strong record and clearly deserves a lot of credit.

Author Rebuttals to Initial Comments:

Referee #1 (Remarks to the Author):

In this manuscript, Karczewski and colleagues describe the gnomAD resource -- aggregate variant calls derived from ~125K human exomes and ~15K human genomes that are already widely used by the research community. The manuscript focuses on predicted loss-of-function (pLOF) variants in particular. The paper is a follow up to the same groups' ExAC resource, published a few years ago, with the overall cohort approximately doubling in size and with markedly better representation of human populations around the world. As general statements, the work appears to be well done from a technical perspective, the analyses thoughtfully conducted, and the material clearly presented. It's obviously been an incredible amount of work to pull these data together, to reanalyze them in a coordinate fashion, and to serve them up to the community in a useful format.

That praise notwithstanding, I do have some high-level as well as more specific concerns.

We thank the reviewers for their comments. We have incorporated most of the reviewers' suggestions, which has improved the manuscript considerably. In particular, we have clarified many parts of the text and added Supplementary Figures and Tables to provide readers with some additional intuition behind the metrics developed and how they correlate with previous metrics of constraint (pLI). We have also added 11 Supplementary Datasets, including constraint summaries, downsampling summaries, and information on genes that are tolerant to homozygous inactivation and compare our metric to previous metrics of variant intolerance.

Major comments

1. The relatively narrow focus on pLOF variation results in some real missed opportunities here. Virtually nothing is said about missense variation, non-canonical splice site variation, or constraint in the non-exome 98% of the genome, for which there are a respectable ~15K genomes available for analysis. My inference is that at least some of these analyses are being slated for other manuscripts, but to be frank I would have preferred an at least modestly more expanded scope. The consequence of this relatively narrow focus on pLOF variants is that the emphasized novelty here, apart from the substantially larger N (as well as what seems to be improvements in methodology throughout, which are not really emphasized), lies with the shift from pLI scores (a continuous metric, but one directed at predicting haploinsufficiency, a binary concept) to LOEUF scores (a continuous metric that reflects the upper bound on a confidence interval for the observed/expected ratio of pLOF variants), together with some of the specific analyses that are performed. This is certainly progress, but it leaves something to be desired if only because feels like a lot is being left on the table in terms of all of the things that one might imagine doing with these data that have nothing to do with pLOF variants.

2. Although the scope could be endless (and although I would love to see at least a bit on the most extremely constrained noncoding regions), the manuscript could be particularly strengthened by adding more content around missense variants in particular. Perhaps I should expect it from past papers, but I am quite surprised by EDF 6f-g in particular and suggest they be moved to the main figures. In particular, the fact that there are so few genes with substantial constraint for missense variants remains surprising to me. Given the considerably higher saturation of missense variants in gnomAD as compared to ExAC, can the authors consider adding regional missense constraint analyses ala the Samocha et al. 2017 paper?

We agree with the reviewer about the wide variety of downstream analyses that can be performed with this data set, but we think that the focus on pLoF variants in this manuscript is important to ensure that the manuscript doesn't become too broad and thus superficial (we note that Reviewer #2 agrees with this). There are indeed many interesting features of missense variation that have been previously discussed[1,2]: as missense variants are an order of magnitude more plentiful than pLoF variants, we believe that the sample sizes in these previous data sets have been generally sufficient to characterize constraint against missense variation at the gene- and sub-gene-level. However, we've now added Supplementary Figure 8 to describe how pLoF and missense variant expectations (genes with over 5, 10, 20, 50, 100 variants) increase with sample size. We found that the increase in sample size from ExAC to gnomAD provides a much more significant increase of pLoF than missense variants.

With respect to non-canonical splice site variation, we characterize these in LOFTEE as "Other Splice" (OS) variants, but their relatively low occurrence and intermediate patterns of depletion in constrained genes (Extended Data Fig. 7d) precluded their inclusion in constraint calculations.

Unfortunately, any attempts at constraint against non-coding elements will be underpowered, even with 15K genomes[3]. At these sample sizes, we do have the power to investigate subsets of non-coding variants with predicted large functional impact, and have a companion manuscript that looks at one such class within 5'UTRs[4]. However, any more comprehensive effort is complicated by the fact that the functional impact of the vast majority of non-coding variants is as yet unknown.

3. While I'm on those panels (EDF 6f-g), it would be helpful if the extremes, particularly on the excess observed vs. expected, points in all three panels could be labeled and ideally discussed. Are there any genes for which significantly more synonymous, missense or pLoF mutations are observed than were expected? Are these technical artifacts or biologically meaningful outliers?

We have also been interested in these outliers, and investigated them at various points in both the ExAC and gnomAD data sets. Unfortunately, in our exploration of the most extreme examples (synonymous $z < -3.71$), the vast majority of these genes appear to be technical artifacts: the worst offenders are *AHNAK2*, *FLG*, and many of the *MUC* genes, which are known to have mapping artifacts, and paralogous genes such as the HIST1 complex. We note that approximately 32% of them (126/392) have a mappability score < 0.9, compared to 10% (1908/18839) of genes that are not outliers for number of synonymous variants. We have added a note to this effect in the Supplementary Information. Overall, while it is likely that there are interesting biological signals in these outliers, identifying those will require extremely careful filtering of all the noise resulting from a wide variety of technical errors, which we think falls outside the scope of this manuscript.

4. The first two paragraphs, although well written, feel a bit misleading. They set up a strawman around the concept that breaking parts of a system can be useful for understanding it, but

then mention Mendelian genetics almost as an afterthought. Understanding human biology through LoF mutations and their consequences has been the mainstay of human genetics for nearly a century. What is being done here builds directly on that foundation, rather than being consequent to a motivation drawn from the engineering field or from our inability to edit LoF mutations into humans. Describing this undertaking as the inverse of classical human genetics (i.e. genotype first, rather than phenotype first; looking for the absence of pLOF variants rather than their presence) would be a more honest representation of the field-specific context into which this work falls.

We agree that we insufficiently credited the role of Mendelian genetics in creating our current body of knowledge about human LoF variants and gene function. We have strengthened the mention of Mendelian disease genetics in the introduction, and also added an additional point about forward and reverse genetics approaches in the discussion.

5. A few things about the LOEUF metric remain unclear to me:

a. No justification is provided for using the upper bound of the confidence interval rather than simply using the o/e ratio itself. I can understand the argument that might be made, but I can also imagine counterarguments. In any case, thought process by which this decision was made should be laid out. The confidence intervals seem to be quite wide? (I'm inferring this from the fact that the median o/e of 48% jumps to a median LOEFF of 0.962).

We have clarified the use of this ratio in the text: "At current sample sizes, this metric enables the quantitative assessment of constraint with a built-in confidence value, distinguishing small genes (e.g. those with observed = 0, expected = 2; LOEUF = 1.34) from large genes (e.g. observed = 0, expected = 100; LOEUF = 0.03), while retaining the continuous properties of the direct estimate of the ratio (see Supplementary Information)." At significantly larger sample sizes, these values will converge and the direct use of the o/e ratio will be more intuitive, but it will likely require ~1 million individuals before we approach this point (estimated at 75% of genes with expected LoFs > 50; 3 million individuals to reach 90% of genes with expected LoFs > 50). These data are added as Supplementary Fig. 8.

b. Unless I missed it, you show histograms of o/e and LOEUF scores, but no comparison of the metrics to one another. Related to point (a) above, it would be helpful to present scatter plots of o/e vs. LOEUF, and also o/e vs. pLI and LOEUF vs. pLI, to provide the reader with a better view on the extent to which they differ?

This is a great suggestion - observing the relationship between these variables really highlights the continuity of LOEUF compared to pLI (which, though it is a value between 0 and 1, is not an appropriate metric to use in a continuous fashion). Further, this reiterates the point above that the confidence interval provides confidence around the value, which for large genes (and eventually at large sample sizes for smaller genes), converges at the o/e value. We have added these scatterplots as Supplementary Fig. 7.

c. Many "summary plots" are presented, but I feel like I still lack a raw sense of how the wide the confidence intervals are for the o/e ratios that give rise to the LOEUF scores, as well as how these confidence intervals vary as a function of the o/e ratios. I suggest that you add a figure that shows the stacked means and confidence intervals for each gene, sorted by means (or a random subset of genes if the entire set can't be presented). To phrase it another way – a plot where every gene has its own line, and the genes are ranked by o/e, and the o/e and confidence intervals for each gene are shown.

We have added this plot to the new Supplementary Fig. 7.

6. What are the considerations involved in estimating selection coefficients from the observed vs. expected data? Rather than deciles or this upper bound o/e value, it would be very helpful to have these estimates, particularly in light of arguments made in this recent paper from Fuller et al. (https://www.nature.com/articles/s41588-019-0383-1) which I urge the authors to cite and discuss in relation to the shift from pLI to LOEUF (and ideally to motivate just going ahead and estimating the strength of selection on heterozygotes on the basis of the updated data in gnomAD).

We agree that accurate selection coefficients would be helpful for interpretation, but generating robust and well-calibrated selection coefficients (as well as the associated uncertainties) would be a non-trivial exercise, and we are concerned about the many ways that such estimates could be miscalculated without a thorough analysis that would (we think) exceed the scope of this paper. In addition, our primary pragmatic goal is the prioritization of disease genes, and we have not yet seen evidence that selection coefficients improve this (our analysis of the s_het metric from Cassa *et al.* (2017) actually shows slightly worse performance for the classification of haploinsufficient disease genes than pLI, using ExAC data for both; we do not yet have corresponding values from gnomAD data).

We do agree that the Fuller *et al.* manuscript provides extremely important caveats regarding the interpretation of constraint-based metrics. We had previously cited this manuscript as a preprint in the discussion, but we have now updated the citation to its current published form. We also thoroughly agree that the estimation of well-calibrated selection coefficients would be very useful for understanding the properties of constraint against LoF variants - we hope that other groups will use the publicly available gnomAD data set to generate and explore such metrics.

7. There are many instances where the authors provide highly significant p-values but no corresponding information about the corresponding difference or effect size. As the authors well know, miniscule effects can have large p-values. I noticed this in particular in the paragraph beginning on line 284 (Biological properties...). However, if the authors could review the full paper for similar instances and provide corresponding effect sizes or fold differences or whatever is appropriate along with the p-values, that would be helpful.

We have added effect sizes and/or the relevant statistics (e.g. means for each group in a t-test) to all p-values.

8. My inference throughout is that when the authors are calculating things such as the proportion of observed variants out of all possible variants, they are including all possible substitutions (3 per site). However, it would be helpful to include at least a bit of information on the extent of "site saturation". For example, when you say 17.2M and 262M variants, is this approaching ~50% of coding nucleotides and 10% of all nucleotides represented in the human genome, at which at least one of three possible single nucleotide changes is observed? Although the primary focus could/should remain w/ keeping all possible substitutions as the denominator, it would be helpful to know the per-site summary statistics as well. This is also relevant to the summary statistics provided at lines 166-169.

We have now computed these statistics at the site-level, which are now in the Supplementary information: "The 14,078,157 SNVs in the exomes span 11,999,542 genomic positions, representing 20.1% of the 59,837,395 bases where calling was performed. When filtering observed and possible sites to a median of 30X coverage, we observe 21.9% of sites with at least one SNV. The 204,063,503 SNVs in the genomes span 192,608,400 genomic positions, representing 6.8% of the 2,831,728,308 bases where calling was performed."

9. Variant calling & indels feel a bit swept under the rug, at least in the main text. I recognize that this is covered in the supplementary figures and methods (and I'm not asking for anything new to be done here; generally convinced), but it would be helpful to have at least a paragraph summarizing the key take-homes from EDFs 2-3.

We have now added a paragraph summarizing EDFs 2 and 3 in the main text.

10. It's not clear how the statement at lines 410-412, that 30% of coding genes are insufficiently powered for detection, is justified. I infer that you are using a cutoff of 10 pLOF mutations expected as the definition of sufficiently powered, but where does this come from? On a related point, at line 230, you state that the increased N from ExAC to gnomAD increases this proportion from 63% to 72%. The relatively marginal increase from doubling the N leads one to wonder – what population

sizes are required to get this even higher? I guess my broader point is that a more formal analysis of power as a function of population size, even if you have to project a bit based on the existing data, would be very helpful (and justify the cutoff of 10 or whatever alternative is chosen). What population size would it take to get to 95% of genes with at least 10 pLOF mutations expected?

The cutoff of 10 was initially described in the supplemental information: “For many of the analyses in this manuscript, we filter the dataset to genes where we expect over 10 pLoF variants. This cutoff was chosen as the minimum number of expected pLoF variants that can result in membership in the most constrained bin (11.1 expected) or pLI > 0.95 (9.43 expected).” We have added a reference to this in the main text. Additionally, and more importantly, we have now added Supplementary Fig. 8 and Supplementary Dataset 12 that describes the proportion of genes with at least N pLoFs expected as a function of sample size. Based on these calculations, it would require ~625,000 samples to achieve 95% of genes with at least 10 pLoF mutations expected.

Additional comments:

11. The claim is made towards the end of the introduction that the metric “improves rare disease diagnosis” – is this actually done in the paper? It seems like this should be rephrased to be more in line with what is actually described in the manuscript (unless I missed something, which is possible).

We have clarified this sentence to “this metric improves interpretation of genetic variants influencing rare disease”.

12. For LOEUF, is is the 95% CI that is used? I assume so, but it should be stated.

We use the upper bound of the 90% CI - this is now more prominently noted in the main text.

13. More could be said, or at least a citation added, for the data points in Fig. 2E that might be explained by hypo-methylated CpGs giving rise to a lower rate of % observed? On a related point, in EDF 4b, is it possible to partition CpG sites on those that are broadly methylated vs. those that are not? (i.e. is it possible that you are completely saturating those that are consistently methylated?)

We have now split Fig. 1e and Extended Data Fig. 4b by methylation status and added these as Supplementary Fig. 5.

14. It would be helpful if the authors review the manuscript specifically to look gene lists that might be useful to readers, and to actually provide those as supplementary tables. For example, at line 216, a set of 1,752 genes that are likely to be tolerant to biallelic activation are referenced but a corresponding file/table not provided.

We have now added the established gene sets as Supplementary Fig. 9, as well as the list of bi-allelic inactivated genes as Supplementary Dataset 7. Further we have compared this latter gene set with mouse and cellular knockout data, and added it as Supplementary Table 19:

Supplementary Table 19 | Comparison of genes we observe homozygous deletion in gnomAD population with other gene lists. Fewer homozygous knockout tolerant genes are included in this comparison (n=1519 vs 1650) as 131 genes that did not have a unique gene symbol approved by HGNC. Further, we filtered out genes from the mouse and cell comparison sets that did not have LOEUF score. For gene set comparisons, the p-value was computed using a Fisher's exact test (two-sided) and for LOEUF comparisons, a t-test (two-sided) was used.

	Mouse Heterozygous KO		Mouse Homozygous KO		Cell Essential		
	Lethal	Others	Lethal	Others	Essential	Others	Mean LOEUF
Homozygous KO tolerant genes (n=1519)	12	1507	87	1432	6	1513	1.26
Remaining genes (n=17675)	383	17292	3647	14028	677	16998	0.91
Odds Ratio		0.36		0.23		0.10	
p-value		6.8×10^{-5}		9.1×10^{-57}		1.5×10^{-17}	$< 10^{-100}$

15. Line 161 – "all possible synonymous methylated CpG variants" – odd phrasing – do you mean all possible CG>TG changes? Or are you subsetting to sites that are consistently methylated? More clarity here would be helpful.

We have edited this text to read "all possible consistently methylated CpG to TpG transitions that would create synonymous variants in the human exome".

16. Fig 3d – the notion of "cell essential" is a little strange. What cell type? Are these genes that are consistently essential across CRISPR screens? More clarity on what these are would be good to include rather than requiring the reader to look at the reference.

This designation is pulled from the Hart et al., reference, but we have added a more detailed explanation in the supplementary text: "Specifically, in the study, Hart et. al. defined a set of essential genes using a strict Bayes Factor threshold, corresponding to >90% posterior probability of being essential for more than six cell lines out of minimum 7 to maximum 12 different screens in different cancer and immortalized cell lines. They defined nonessential genes based on low RNA expression level across 17 different cell lines, as well as curated shRNA screening results, and this was validated with CRISPR/Cas screening."

17. In Fig. 5c, there are few enough points above the cutoff that it would be better if you could find a way to just label them all. But on a related point, I think the corresponding section of the discussion is too bold. Unless I'm missing something, the last sentence of the results feels too bold. The results primarily follow from a few phenotypes, all related to brain function, and possibly conflated with one another. It would be better to restrict the claim to this subset of phenotypes rather than 'many heritable polygenic diseases and traits'.

With respect to Fig. 5c, we had tested layouts with all the points above the line labeled and could not find anything that was readable. However, we've now added Supplementary Table 17, listing any trait with $p < 1e-4$ and their summary statistics, and the full dataset as Supplementary Dataset 13. We have revised the sentence to read: "and suggests that some heritable polygenic diseases and traits, particularly cognitive/psychiatric ones, have an underlying genetic architecture driven substantially by constrained genes".

18. Less a request than a suggestion, but I would love to see more discussion or at least some brief quantification of the proportion of top-decile LOEUF genes (i.e. most constrained; n = 1,920) in terms of what % are associated with a human phenotype, what % have an assigned function, what % have no known function, etc.

We have found it quite difficult to rigorously define "assigned function" in a high-throughput way. However, we have characterized the % with no known ligands in Extended Data Fig. 8a, and the % associated with a disease phenotype in Extended Data Fig. 9a-b.

Referee #2 (Remarks to the Author):

Summary

In the manuscript entitled “Variation across 141,456 human genomes and exomes reveals the spectrum of loss-of-function tolerance across human protein-coding genes”, Karczewski and colleagues describe the Genome Aggregation Database (gnomAD). This is a substantial augmentation to the Exome Aggregation (ExAC) database, including additional exome data as well as adding 15,708 genomes. The authors describe the data contained within this resource, the quality control measures, and highlight a particular variant class, predicted loss of function (pLOF). They develop a metric termed LOEUF that is used to categorize genes with respect to their tolerance for loss of function variation. They then provide examples demonstrating how this information can be used in disease gene research.

High level comments

The impact that the gnomAD and ExAC resource has had on both the research and clinical genomics communities cannot be overstated. The dedication this group has to making this data available to the research and clinical communities is exemplary. This project has been a flagship for demonstrating how large-scale sequencing coupled with summary data release can be transformative for the field.

With respect to this manuscript, the authors were wise to focus on a narrow aspect of this data and demonstrate how this specific variant type (pLOF) can contributes to disease gene research. The manuscript walks through how the LOEUF deciles are generated, and provides numerous analysis of how this can be used. However, the manuscript fails to connect this with specific biology that resonates in an impactful way. There are no specific examples pulled from the categories to make the data more meaningful. For example, when looking at figure 3, there is so much more information I want to know. This includes identifying specific genes that fall into expected LOEUF deciles, but also discussing the unexpected events. What are the haploinsufficient genes that are in the lower LOEUF deciles? Does any of this information confirm or refute known orthogonal data about genes in these bins? Perhaps even just noting where the ACMG59 fall in these deciles, or within these analysis would be useful. Though I understand that the ACMG59 may not be under the strongest constraint, a data set such as this could help guide the community about the best way to utilize this data.

I do think there are some other missed opportunities for educating users around some issues of this analysis. While this manuscript focuses on global analyses, it is important to remember that many users will look at individual genes, and understanding how individual genes may be impacted by analytical decisions is important for ensuring the data are used correctly.

We thank the reviewer for these comments. We completely sympathize with the desire to look at individual outlier genes for multiple analyses - indeed, this desire, and the fact that we often can't predict which genes our users will find most interesting, has been a primary motivation for releasing the variant list in full to enable the community to perform their own analyses, and a browser for users to explore their favorite individual genes or gene sets.

We have added a discussion of the haploinsufficient genes that are unconstrained to the Supplementary Information: "Of the haploinsufficient genes, 80% were found in the two most constrained deciles of the genome. There were two genes that are in the haploinsufficient gene list, but with little evidence of constraint (in the 8th decile): *RNF135* (LOEUF = 1.44), which has limited support for pathogenicity[5]; and *IKBKG* (LOEUF = 1.37), which is poorly covered in gnomAD and whose first exon is lowly expressed, suggesting that the pLoFs in this gene are likely false positives." In general, we think that the ACMG59 genes are a rather confusing comparator in these analyses, due to their ascertainment on the basis of clinical utility rather than any metric that might correlate with selective constraint (such as age of onset, inheritance mode, or phenotypic severity). However, we show the distribution of the LOEUF scores for these genes below. We also note that 5 out of the 59 are not powered for constraint detection (fewer than 10 pLoFs expected); these are noted in the supplement: *SDHD*, *MYL3*, *VHL*, *MYL2*, *SDHAF2*.

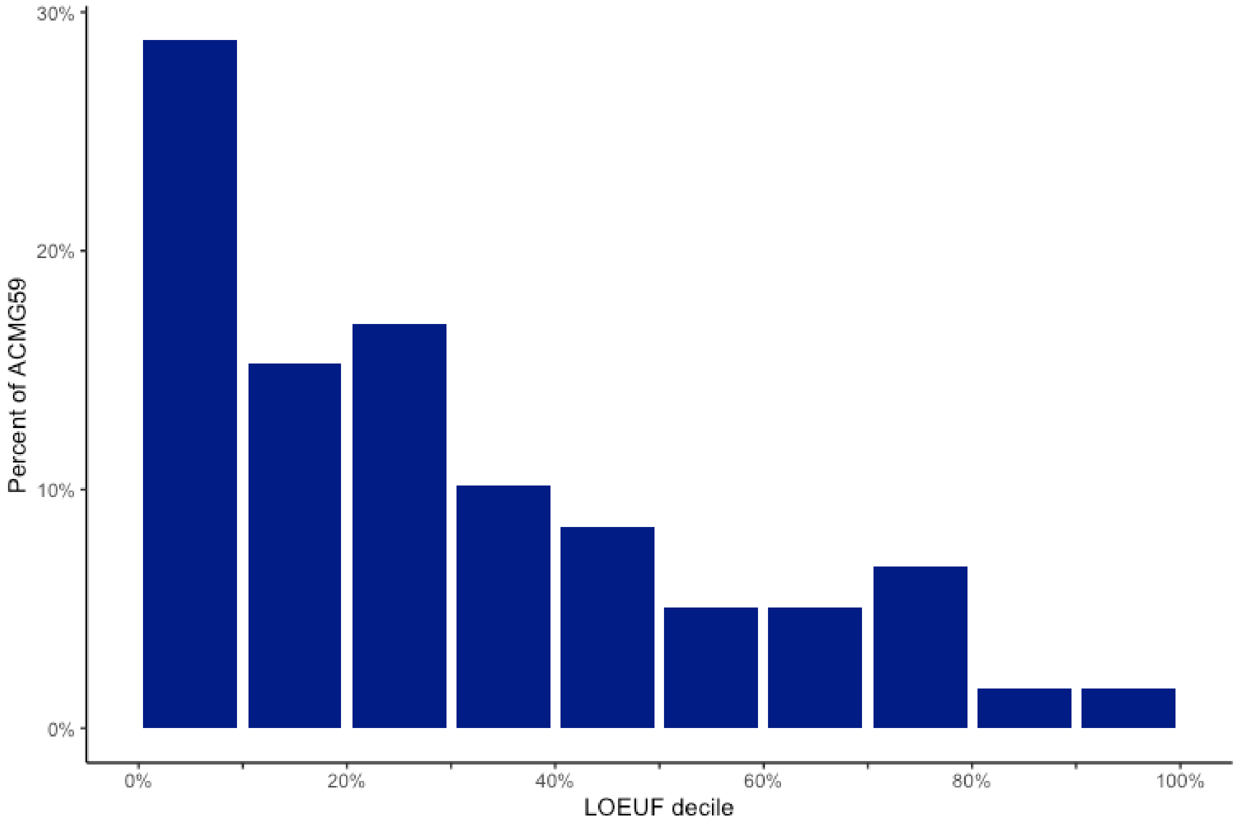

More detailed comments

1. pLOF set definition: The authors are very well versed in the types of annotation errors that can lead to false variant calls, and understand the pLOFs are likely to be enriched in annotation

errors. While I think the authors do a very commendable job of cleaning this dataset, I would urge the authors to review a recent manuscript from Tuladhar et al., 2019 (http://dx.doi.org/10.1101/583138) which is focused on analyzing putative knockout alleles from CRISPR lines. In this manuscript, they find that 46% of edited lines marketed as knockouts, due to the presence of an indel, are not actually knockouts, but that some product is produced via other escape mechanisms. While I don't think the authors need to any additional experimental work for this manuscript, this reference, plus additional caution that individual pLOF events need to be followed up with functional assays to confirm actual LOF is warranted. Particularly because the authors go to so much effort to produce a high quality dataset, users need to be reminded that while collectively, the data are of high quality, individual events should be verified. It might be interesting to run LOFTEE on the dataset in the Tuladhar manuscript to see how many of the 'escapees' are flagged by LOFTEE.

We've read this paper with great interest and agree that an exploration of further NMD-escape modes is worthwhile. Unfortunately, the Tuladhar paper does not appear to include a list of variants on which we could run LOFTEE. Nevertheless, we have cited this paper as well as the "Genetic paradox explained by nonsense" paper mentioned by Reviewer 3, and discussed the implications of our work around these: "However, some additional error modes may still exist, and indeed, several recent experiments have proposed uncharacterized NMD-escape mechanisms[6,7]."

2. Variant identification: There are two points to be raised here. The authors do a great job of trying to eliminate false positives, even at the expense of potentially losing true positives. However, the authors don't mention the impact of false negatives. For example, STRC has very poor coverage of exons 19-25 due to the presence of a paralogous sequence in the genome, complicating alignment and leading to low or no coverage. What percentage of the genome, particularly the clinically relevant genome, falls into this category? Does gnomAD do better/the same/worse in difficult regions such as those as described in Mandelker et al., 2016 (https://doi.org/10.1038/gim.2016.58)? Does the genome data help in some of these cases? Also, do the authors have any comments on the impact of genome data vs. exome data in terms of variant identification? What are the technical advantages/disadvantages of genome vs. exome? For example, when looking at PKD2 in the gnomAD browser, it looks as if genome data rescues poor coverage of the first exon as seen in exome data. How often does this sort of thing occur?

We thank the reviewer for this comment (and reviewer #3 for a similar comment). This analysis is somewhat complicated as gnomAD has aggregated data sequenced over a long period of time, spanning different capture kits / sequencing technologies. To evaluate the performance of those different platforms in the coding regions of the genome, we have now computed for each gene the proportion of bases that are well-covered (20x in at least 80% of the samples). We have added a section in the Supplementary Material showing that ~80% of protein-coding genes are well-captured by all technologies, whole-genome sequencing captures ~8% additional genes well, and about 2.5% of the genes are not captured by either. Further, we also showed that the majority of genes that

aren't well captured by whole-genome sequencing have poor mappability. We break these analyses down further by capture platform and sequencing technology in Supplementary Figures 3 and 4. Finally, we added a table with per-gene, per-platform coverage summary statistics as Supplementary Dataset 1 and for download at https://storage.googleapis.com/gnomad-public/papers/2019-flagship-lof/v1.1/summary_gene_coverage/gencode_grch37_gene_by_platform_coverage_summary.tsv.gz

3. More details on specific genes: There are a few places that summary data is provided, and what I really want are the details. For example, on line 216, there is a statement about 1752 genes that are likely intolerant to biallelic activation- what are these genes? I expected a supplementary table, but there is none (though apologies if I missed this). I had the same reaction to Supplementary table 15- I would love to see a giant table, one row for each gene with the classification (column headers in this table) and LOEUF decile. This would really let me dig into some interesting stuff. Are there any surprising genes in these deciles (lines 245-249). In fact, some of the disease genes that unexpectedly fall into the lower LOEUF categories would be some of the first ones I'd want to test for an escape mechanism leading to expression despite the prediction of LOF.

We have now added Supplementary Dataset 3 with a list of the genes tolerant of homozygous inactivation, and Supplementary Dataset 11 with all the constraint and summary metrics for each gene in the genome. While Supplementary Table 15 (now 17) has too many genes to list in a table, we have added Supplementary Figure 9, a high-resolution figure, where readers can zoom in to see specific genes/gene sets.

4. Figures and data in general: I found the availability of numbers and consistent metrics that support data figures was inconsistent. Ideally, these numbers would be contained within the figures or at least the figure legend (for example, every time there is a correlation, I'd like to see the correlation values in the figure or legend, not just in the text). I found myself having to go back and forth between the text and figures a lot, and occasionally I thought I found inconsistencies, though I'm not always sure if the data are inconsistent, or I'm just having trouble matching the text to the appropriate figure. For example, line 225-226 notes 'the variation in the number of synonymous variants observed is accurately captured (r2=0.958)'. The data in extended figure 6f, which states r=0.9791- so consistent use of either r or r2 would be appreciated. Even just ensuring that numbers are in figure legends, if not the figure, is useful for more easily interpreting the figures. Please review that legends and colors are clear. For example, what do the colors in figure 5c mean? I spent an embarrassing amount of time looking for the 'circles' in extended Data Figure 1, to realized the rounded corner squares were what I should look for.

We have now fixed the text to consistently use r rather than r^2, and fixed these and many of the other areas where statistics were missing or inconsistent. We have also clarified the use of color and shapes in the aforementioned figures.

5. Assembly information: While I am sympathetic to the needs to use the woefully old GRCh37 assembly (I myself had to do this for a recent manuscript) it is useful to explain to users why this very old reference is used (page 3 of the supplement) and what the shortcomings are. While I firmly believe the reference assembly version used will not impact the overall findings of this paper, it may impact the information at any given locus. For example, a big focus of the GRCh38 update was to improve clinically relevant genes (for example, adding in 3 missing coding exons of Shank3, and adding a new paralog of KCNE1 which means many of the variants called in GRCh37 may actually be paralogous sequence variants) and users should understand these caveats. This also impacts the pLOF variant curation, as regions known to be different between GRCh37 and GRCh38, as well as known assembly problems from the GRC would likely be useful in this analysis (supplemental page 32). Lastly, please use consistent nomenclature when referring to the assembly. The official assembly name is GRCh37, but there are various distribution 'flavors'. And while I weep that this is the case, it is important to note the data source (hg19 specifically implies data from UCSC for example) so that these slight variances can be taken into account.

The underlying data used for this manuscript (the exome and genome callsets) are now more than three years old, having been produced in 2016 (with most of the read mapping having been performed in 2015 or prior). While the GRCh38 assembly had already been produced at the time, the GRCh37 assembly was still the field standard. We agree that in 2019, producing large genomic resources based on the GRCh38 assembly is imperative, and is therefore what we plan to do for future versions of gnomAD. We have fixed all references to be GRCh37 rather than hg19.

6. Variant annotation and constraint modeling: The supplement sections read as if they are stand alone sections. I understand why this happens and in many cases this is not a problem, but I had some trouble understanding where variant annotation, and some of the gene level metrics stop and the constraint modeling starts. As I was reading the main text, I was curious as to how regions of low coverage (and thus potential false negatives) impacted the LOEUF and gene level metrics. Does something look more intolerant to LOF because no variants are called because of low coverage? The constraint modeling section of the supplement explains how coverage is taken into account, but the variant annotation section, which has some information on gene level metrics, does not. My general assumption is that these two sections really work together in a way that is not entirely clear to me from reading the text, but perhaps I am wrong about that. It would be nice to clarify some of these metrics with some examples - i.e what calculating the data looks like on a well covered gene versus a genes like STRC, SMN1 and IKBKG (thank you for the lovely browser that made looking these examples up relatively straightforward!). Additionally, Figure 2D highlights that 30% of coding genes in the genome are still underpowered to detect constraint - how many ClinVar or ACMG59 genes fall into this category?

The reviewer is correct about the treatment of coverage throughout the manuscript. For the purposes of tallying the *observed* number of variants (both for constraint and gene metrics), we require a genotype to have a depth of at least 10. Only in the constraint section do we explicitly model the mean coverage across individuals at a site in order to accurately estimate the expected number of pLoFs for genes with low coverage, which feeds into the LOEUF calculation. Thus, a gene will not look more intolerant to LoF simply because of low coverage, but instead, this would lead to a decrease in detection power, which is the desired behavior. Indeed, this does mean that the aggregate pLoF frequency metrics may be deflated at these genes; however, we have no way to explicitly correct for this as these are summary metrics derived straight from the data.

Of the ~28% of genes that are underpowered for constraint detection, these are - perhaps unsurprisingly - depleted for disease-associated genes. We have added a note to this effect in the Supplementary Information: "At present, 72.1% of genes (13841/19197) have > 10 pLoFs expected, including 86.5% of disease-associated genes from OMIM (2888/3340; OR = 0.45; Fisher's $p < 1 \times 10^{-100}$). Of the 59 genes satisfying ACMG criteria for reporting of secondary findings, only five are underpowered, or have fewer than ten pLoFs expected (*SDHD*, *MYL3*, *VHL*, *MYL2*, *SDHAF2*)."

7. Exon level metrics: Have the authors considered calculating these metrics at the exon level rather than the gene level? Would this provide even more fine grained information? If a gene only has a small number of exons under constraint, could it end up in one of the higher LOEUF deciles depending on gene size? Could this potentially improve variant interpretation? Or would doing this require significantly more samples? There would likely be utility in doing this at the exon level if statistically achievable.

Unfortunately, the calculation of these metrics at a per-exon level would be highly underpowered: even at the gene level, we only reach 10 expected pLoFs for ~70% of genes, and breaking this down by exon would reduce this number accordingly. However, the code provided can compute constraint against arbitrary bases, and thus will be usable when sample sizes grow. In the meantime, we have described a method that removes bases within exons with little to no evidence of transcript expression[8] that shows the power of this approach.

Referee #3 (Remarks to the Author):

In their manuscript entitled 'Variation across 141,456 human exomes and genomes reveals the spectrum of loss-of-function intolerance across human protein-coding genes' Karczewski et al. present the largest human exome/genome dataset published to date.

This is without a doubt a very valuable resource to the field of human genetics/genomics; clinical researchers, diagnostics, etc. Using a set of >440k high confidence pLoF variants, i.e. a set that is more than double the size of ExAC and by applying an improved model (utilizing methylation-base-

pair level coverage correction and LOFTEE) they classified the level of LoF intolerance of all protein coding genes.

This expands beyond the use of ExAC for many aspects, which increases the usability to novel reads/users, e.g.:

- More than double sized dataset
- More populations represented
- Exomes plus genomes included
- Options to use dataset with or without certain sub-cohorts (e.g. non-cancer cohorts)
- Isoform refinement

The power of this dataset is confirmed by: a) constraint metric correlated with biological relevance (PPI; gene expression; disease association); b) the constraint metrics reflect model animal and cellular KO phenotypes; c) constraint can assist disease gene finding (ratio 15 higher likelihood for de novo mutations in developmental disease genes in LOEUF decile).

There are however several major aspects that require refinement, and several new aspects could additionally boost the scientific value, add novel insights or increase the usability even more.

1.) It would be interesting to the readers, in which aspects the authors improved over previous work: MacArthur et al. Science (2012) (DOI: 10.1126/science.1215040); Lek et al. Nature (2016)(DOI: 10.1038/nature19057); and which of the previous conclusions may have been falsified since then.

We have added Supplementary Fig. 10 and 11, which both give a good sense of the increase in power for assessing constraint as sample sizes increase. We're not aware of any major conclusions from previous papers that have since been falsified, but we are now able to give a more refined estimate of the average number of LoF variants per individual (Supplementary Table 16, and Supplementary Datasets 8-9) - this number has stayed surprisingly consistent since the 2012 paper, despite substantial changes in sequencing accuracy and gene model curation over that period.

2.) The authors should discuss their findings in light of the recent set of work on 'Genetic paradox explained by nonsense'; https://www.nature.com/articles/d41586-019-00823-5

We've read this paper with great interest, but unfortunately the proposed mechanism cannot be assessed using genetic data alone. We have added a note about this paper to the discussion: "However, some additional error modes may still exist, and indeed, several recent experiments have proposed uncharacterized NMD-escape mechanisms[6,7]."

3.) Add a paragraph how many LoF allele human individuals carry on average, per population per frequency range

We have added a summary of this information as Supplementary Table 16, and a full breakdown in Supplementary Datasets 8-9.

4.) The author should consider flagging genes for which the majority of pLoF variants appear in a) smaller allelic fractions indication somatic/mosaic state; b) in >average aged individuals. Both would be very indicative for 'drivers of clonal hematopoeisis'; and may prevent false interpretations of pLoF in genes like DNMT3A, ASXL1, TET2 (which in germline may very well cause severe developmental diseases caused by AD mutations) as well as flagging up novel genes with a similar mechanism and biology.

This is a great suggestion. We have previously shown that pLoF variants in *ASXL1* are very clearly found in older individuals and at lower allele balances in ExAC[9], but haven't systematically explored the impact of CHIP using gnomAD. We have now performed a full analysis of this phenomenon and added a section to the supplementary information, "Genes affected by clonal hematopoiesis". We searched for genes in which LoF variants were present at lower allele balances in older individuals compared to synonymous variants. This analysis confirmed that significant signals of clonal hematopoiesis of indeterminate potential (CHIP) are present in the known CHIP-associated genes *DNMT3A*, *ASXL1*, and *TET2*, but did not reveal any novel genes passing a genome-wide significance threshold with a similar mechanism.

5.) Can the authors describe for which genes/exons the WGS vs WES data improve sensitivity ('dark areas' of exomes)?

Another great suggestion - this prompted us to look into how genomes add power even within protein-coding regions. We have now computed for each gene the proportion of bases that are well-covered (20x in at least 80% of the samples) for each of the sequencing platforms in gnomAD. We have added a section in the Supplementary Material explaining how this was computed, showed overall results in Supplementary Figures 4-5, and have released a file with coverage summary for each gene and each platform (https://storage.googleapis.com/gnomad-public/papers/2019-flagship-lof/v1.1/summary_gene_coverage/gencode_grch37_gene_by_platform_coverage_summary.tsv.gz).

6.) Can the authors provide data on compound heterozygous state of pLoF variants in individuals? This would be very informative for a) adding sensitivity that gene that can/cannot tolerate complete Kos; b) show alleles for which frameshifting variants are rescued by other frameshifting in cis in order to restore the reading-frame.

Analyses of compound heterozygosity require large-scale inference of variant phase, which is a worthwhile analysis, but one that will require substantial dedicated work and that we believe falls outside the scope of this paper.

However, we completely agree that the degree to which frameshifting variants are rescued by other frameshifting variants in *cis* is worthwhile, and we have generated the list of such indel pairs up to 30 bp distance each other. These have been made available at https://storage.googleapis.com/gnomad-public/release/2.1/frame_restoring_indels.tsv. As we felt these analyses were better suited to our companion manuscript on multi-nucleotide variants[10], we have also added a figure set to that manuscript to describe the basic property of such indel pairs, such as:

- The proportion of in-phase indel pairs is very low when the distance is >30 bp
- The most common pattern of frame-restoring indels results in 0bp insertion/deletion (e.g. 4bp deletion + 4bp insertion)
- such indel pairs are most commonly found in HLA genes

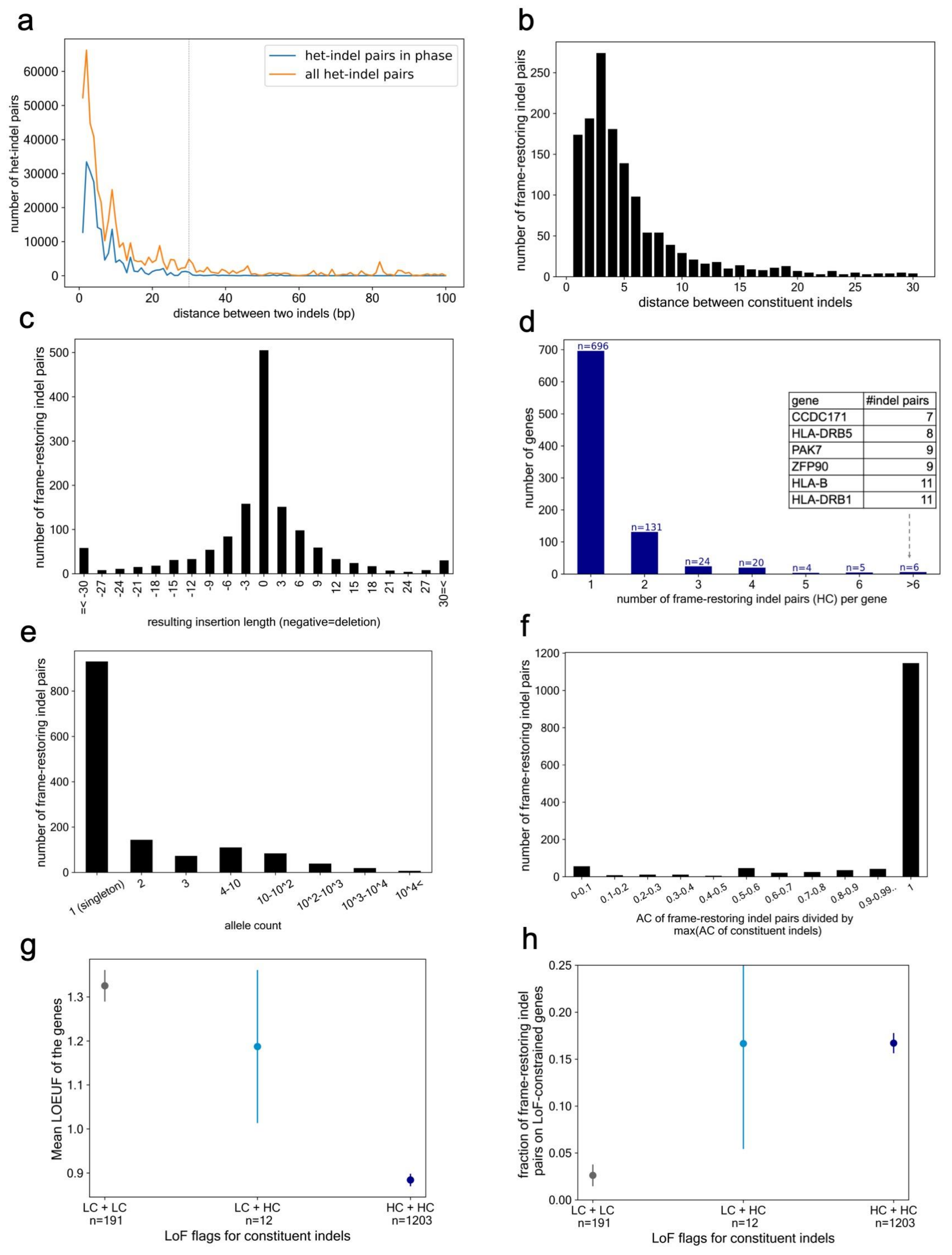

Figure S3 (of the gnomAD MNV paper) [10]. Properties of frame-restoring indel pairs

a, The number of indel pairs (orange = all, blue = phased) is shown as a function of distance between the indels. We set the threshold distance to be 30 as there are relatively few indel pairs past this distance. **b,** The distribution of the distance between indel pairs resulting in frame restoration (exome only, same for c~h). **c,** The distribution of the resulting insertion or deletion length for frame-

restoring indel pairs. **d,** The number of frame-restoring indel pairs per gene, and the list of genes with more than six such variants. **e-f,** The allele count distribution of frame-restoring indels **(e)** and the distribution of allele counts divided by the maximum allele count of constituent SNVs **(f)**. The value is exactly 1 (implying LD r^2 = 1) for 81.5% of overall frame-restoring indel pairs, suggesting that majority of such indel events are likely the result of one mutational event. **g-h,** The mean LOEUF (constraint) score **(g)** and the fraction of LoF-constrained genes for frame-restoring indel pairs **(h)**, per combination of LOFTEE filters of the constituent indels.

7.) Next to the CNV/SV dataset in preparation (Collins et al.); have the authors compared LOEFF decile genes for overlap with CNV morbidity map (Eichler lab; Cooper et al and Coe et al.); and HI scores by the Hurles lab?

The SV companion manuscript[11] has a comparison of the SV calls with the CNV morbidity map, and we have previously compared our constraint metrics to the HI scores from the Hurles lab and find a high correlation (Supplementary information of [1]).

8.) It would be very interesting to understand whether there are genes that are exclusive or enriched for certain types of pLoF. E.g. are there genes that show stop-gains only but no frameshifts or essential splice site pLoF?

Most genes, especially highly constrained genes, have fewer than 5-10 observed pLoF variants per gene, and thus, a systematic comparison within a gene across the three classes of pLoF variants is likely to be underpowered for most genes. For genes with many pLoF variants, many of these are likely to be false positives and a systematic assessment would yield primarily signals related to false positives (especially enrichment of indels at repetitive sites). While this analysis would be interesting, we think it will require a larger sample size and further improvements in variant filtering before the results are meaningful.

9.) Are there any specific pLoF alleles that are significantly enriched in certain populations? E.g. are there any population specific PCSK9-like alleles?

There are many pLoFs that are private to each population and it is quite difficult to assess the biological importance of enrichments of any given variant in the absence of associated phenotype data. The vast majority of pLoF variants are rare, found in one or only a few individuals (typically from the same population). Thus, the QQ plot for enrichments would be hyper-inflated, the multiple testing burden of such an analysis exorbitant (0.5M variants * 7 populations), and the interpretation of results very difficult.

10.) How many isoform specific effects are (not) re-solved by transferring from hg19 to hg38?

As this analysis was performed on hg19, we have not yet assessed the improvements added by alignments to hg38. The next gnomAD dataset will be natively aligned to hg38, which will enable comparisons of the two references.

Minor issues that may further improve the manuscript:

1.) Line 56: "model of human mutation" isn't this rather "mutation rate"

2.) Line 91: Mention somatic events (and differences in tissues source) as a source of 'false positive germline events'

3.) Line 161: Please add an explanation and citation to the synonymous methylated CpG variants – as the most mutable site of the human genome.

Thank you - these are now all corrected (the last of these is now: "These variants reflect the expected patterns of variation based on mutation and selection: we observe 84.9% of all possible consistently methylated CpG to TpG transitions that would create synonymous variants in the human exome (Supplementary Table 14), indicating that at this sample size we are beginning to approach mutational saturation of this highly mutable and weakly negatively selected variant class").

4.) Line 169 (and ext. fig 4): The authors should be able to model the amount of exomes/genomes required to robustly reach saturation across all mutational contexts.

This is definitely possible, and in fact we have previously described a method of modeling and predicting variant saturation at different sample sizes (up to 500K) and applied it to ExAC data (see [12], and especially Figure 1b).

5.) Lines 237-238: Could the author define how much the refined model and the increased sample size to the improved power?

Unfortunately not - while we agree that it would be useful to understand the relative impact of these two factors, it would be very expensive in computation and reformatting labor to re-run the new model on older data or *vice versa*.

6.) Line 475: change "sex aneuploid" to "sex chromosome aneuploid".

7.) Figure 6b: define "mu" in legend.

These are now spelled out.

8.) Supplement: page 4; why was coverage capped at 100x, and are there any adverse effects expected for capping?

The coverage was capped at 100X for efficiency of computing coverage; we have now added a note to this effect in the supplement. With respect to adverse effects, the effects of coverage on constraint calculations is shown in Extended Data Fig. 6e, and there is very little effect even above 50X.

9.) Supplement: page 46: some references are not formatted correctly at first citation (Hamdan; Lelieveld).

Thank you - these are now corrected.

Referee #4 (Remarks to the Author):

In, “Variation across 141,456 human exomes and genomes reveals the spectrum of loss-of-function intolerance across human protein-coding genes“, Karczewski et al. describe a compilation of variants in exome and genome sequence data from over 100,000 individuals assembled from a variety of projects. They focus on predicted loss-of-function (pLOF) variants inferred to eliminate protein production, and describe pipelines to effectively remove erroneous pLOF variants. They then quantify the extent of observed pLOF variation across populations and genes, asses the relationship between pLOF and transcript expression, and define pLOF gene-level tolerance scores for application in human disease genetics.

In general, this work is of high technical quality (a few minor comments are provided below). Further, the authors are to be commended for their efforts to not only make the data public and usable but also to publish software to generate/parse/filter/etc. GnomAD/ExAC has been a highly impactful resource and this iteration is likely to continue in that regard.

However, my high-level opinion is that while the underlying resource is impressive, this manuscript is a narrow one documenting only incremental advances over previous work. The novelty here is largely due to the increased sample size and in refinements to the methods, e.g., the machine learning approaches to filter variants and the model to infer mutability, but the key concepts and conclusions have been previously published. For example, a key message in this paper, i.e., that mutational tolerance scores usefully separate genes according to phenotypic relevance, is similar to that of Petrovski et al., published in 2013. The distributional shifts presented here in Figures 3a,c,d and 5a, are similar to those shown in Petrovski et al. Figures 2 and 3; in fact, Petrovski et al. used nearly identical types of genes to make the same point (i.e., haploinsufficient, mouse-lethal, OMIM-dominant, OMIM-recessive, and neurodevelopmental-disorder genes). This manuscript is part of a large group of studies that use related methods and lead to similar conclusions about the inference of selective tolerance as a means to identify pathogenic variation (non-comprehensive examples beyond Petrovski et al. include Fu et al. 2013, Samocha et al. 2014, and Gussow et al. 2017).

Thus, the difference between this and previous work is of degree not kind. Towards that end, this analysis does not systematically and precisely measure improvement over previous work, nor is there a systematic delineation of the effects of the various sources of improvement described. For example, while sample size is analyzed in relation to variant saturation, no comparison of LOEUF gene group separation efficiencies (e.g., haploinsufficient, essential, ID/DD, etc) at various sample sizes is demonstrated. Similarly, the variant filtering and mutability models developed here are not contrasted with other models provided the same input data (e.g., RVIS on the same set of pLOF variants), nor are the effects of the differing refinements described here measured as isolated components (e.g., LOEUF on VQSR vs machine-learning-filtered variants or a simple mutability model vs a CpG/methylation/etc-defined model). While I find it highly likely that the results described here are non-trivially more powerful for separating genes known to be relevant to phenotype from those that are not, the improvements are likely to be modest; the more important, pragmatic effect on gene discovery per se is likely to be even smaller given that there is not a strict monotonic correlation between the distributional separations benchmarked here and novel disease gene prioritization effectiveness.

While the overall concepts of LoF annotation and constraint have been previously described, we have added substantial variant filtration improvements (e.g. the RF filtering process and LOFTEE) and methodological improvements (such as changes to the underlying mutational expectation model, and the development of LOEUF, a continuous form of the previously published concepts; e.g. pLI). In combination with more than doubling the underlying sample size, these changes have considerably increased the resolution for the detection of LoF constraint in human genes.

A systematic assessment of the relative impact of each of the filtering and model components (and their combinations) would be extremely time-consuming. However, we have added a comparison of LOEUF to RVIS, which is described in the Supplementary Figure 10: while we could not find the RVIS code to run on the exact same set of pLoF variants, we used the publicly available set of RVIS scores on gnomAD variants that was available at http://genic-intolerance.org (RVIS_Unpublished_ExACv2_March2017.txt downloaded on July 15, 2019). Further, we computed the effect of increasing sample size on LOEUF, which is now shown in Supplementary Figure 11.

Comparison to previous metrics of essentiality

We compared LOEUF to previous metrics of genic essentiality, including pLI and RVIS. pLI was computed on the gnomAD exome variants in this manuscript as described previously[1] and RVIS[13] scores for gnomAD were downloaded from http://genic-intolerance.org/ (RVIS_Unpublished_ExACv2_March2017.txt downloaded on July 15, 2019). We selected two gold standard datasets for comparison: 1) the haploinsufficient gene list described in "Gene list comparisons", and 2) a union of the mouse heterozygous lethal and "cell essential" gene lists described in "Mouse and cell model comparisons." Using these genes as "true positives" and all other genes as "negatives," we created receiver operator characteristic (ROC) curves for each method and computed the area under the curve (AUC) as a performance assessment. LOEUF substantially outperforms RVIS for both gold standard sets, and performs similarly to pLI for identifying haploinsufficient genes and outperforms pLI for essential genes (Supplementary Fig. 10).

Supplementary Figure 10 | Comparison to other gene essentiality metrics. ROC curves for each gene essentiality metric, for discerning haploinsufficient genes (**a**) or mouse heterozygous lethal or cell essential genes (**b**).

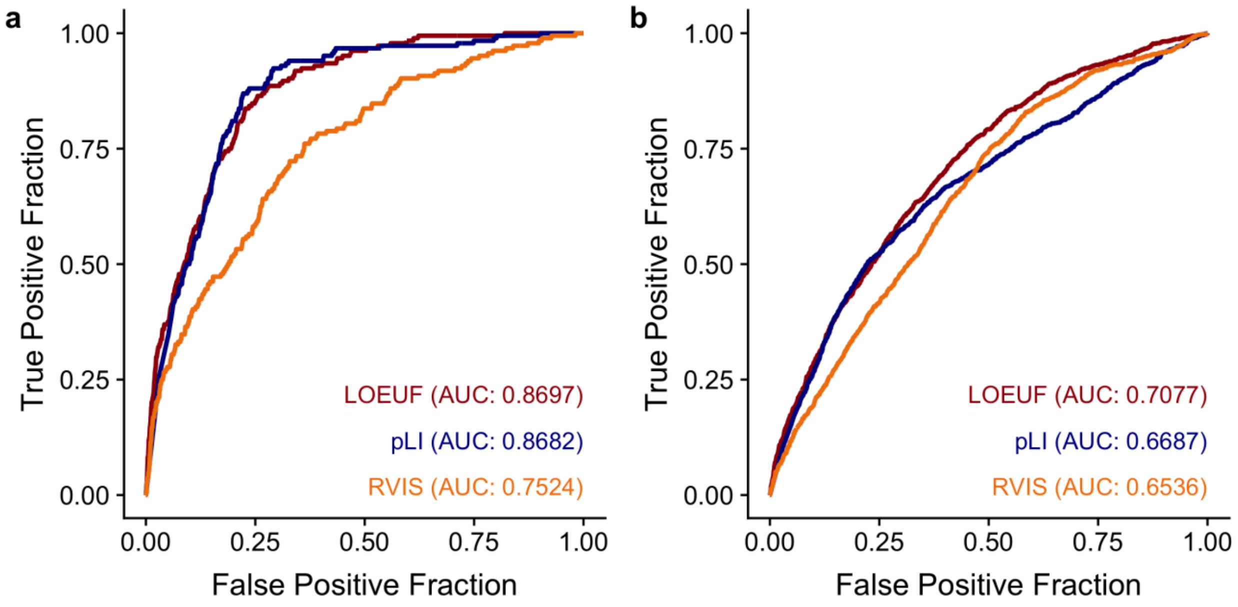

Performance as a function of sample size

We repeat the ROC process described above for each of the computed LOEUF scores for each downsampling of gnomAD and find that the performance of LOEUF is dependent on sample size and not yet saturated for identifying haploinsufficient genes (Supplementary Fig. 11).

Supplementary Figure 11 | Performance of LOEUF by sample size. Area under ROC curve (AUC) for LOEUF computed for various downsamplings of gnomAD, for discerning haploinsufficient genes (**a**) or mouse heterozygous lethal or cell essential genes (**b**).

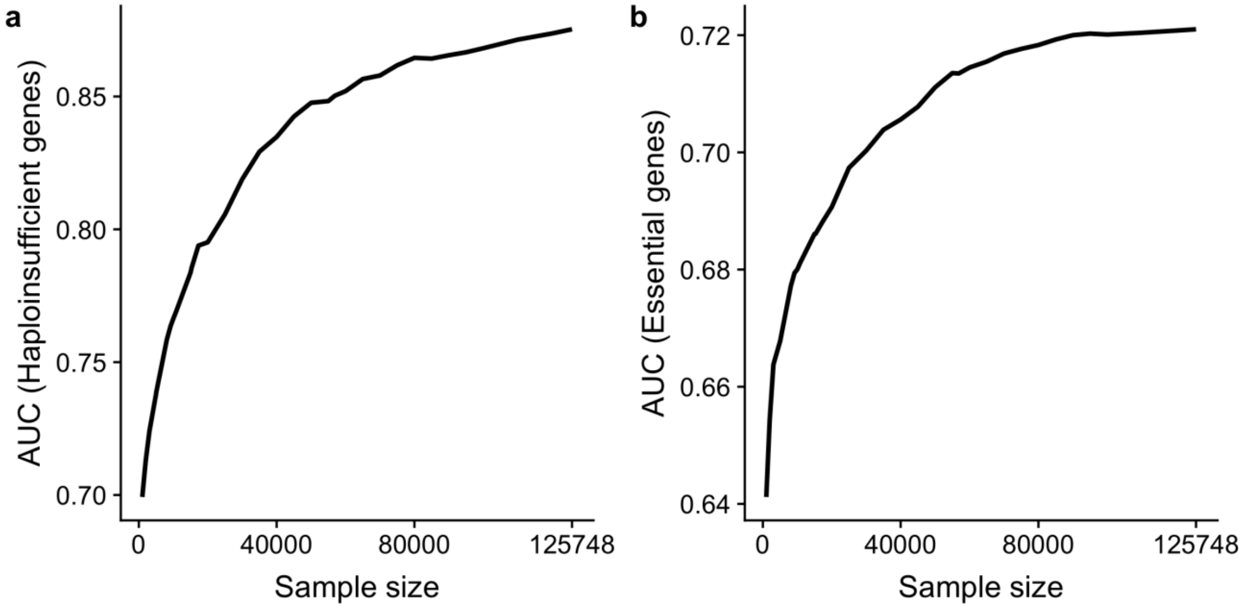

Other key results, such as those related to the contribution of errors to pLOF variants and the relationship between nonsense variants and expression are also conceptually similar to previously

published studies, including some by many of the authors here (e.g., MacArthur et al. 2012, Bartha et al. 2015, Rivas et al. 2015, Balasubramanian et al. 2017, Ganna et al. 2018).

As the reviewer previously noted, we are committed to rapid, open-source release of methods. This manuscript describes in detail the LOFTEE filtering strategy and software package, which, while it was used in previous work due to being freely available for years ahead of publication, has not yet been published in its current form. While LOFTEE implements many of the filters previously described, there are a number of optimizations, including a conservation-weighted base truncation scheme, splice-rescue variants, as well as the inclusion of non-canonical splice variants, that have not been previously described. We have now added a note about the splice rescue variants in the main text, and these are described in Extended Data Figure 7 and Supplementary information.

The preexisting literature on de novo variation in ID/DD, another highlighted result in this manuscript, is too extensive to concisely summarize or cite here, but it is safe to say that the key results here (e.g., Figure 5a) have already been seen in numerous studies that use related approaches and similar data.

While we agree that the enrichment of *de novo* variants in DD/ID patients has been previously described, the enrichments here are stronger than those in previous works, partly as the continuous nature of LOEUF permits the finer-grained exploration of highly-constrained genes.

I am not arguing that this manuscript offers nothing distinctive relative to the other cited manuscripts (and the other uncited manuscripts like them). Indeed, I find it likely that there are benefits to the increased sample size and methodological refinements described here. However, these differences are not systematically and precisely quantified, and even if they were I do not believe they would be conceptually or pragmatically large.

There are also some key details and points outsourced to accompanying manuscripts cited as being in preparation, including Cummings et al., Collins et al., Minikel et al., and Whiffin et al., suggesting result overlap that further undermines uniqueness and novelty here. While not cited as such, it appears that these manuscripts are available on Biorxiv (in my opinion, “in preparation” or “data not shown” citations are intrinsically inhibitory to meaningful review and should not be used). After reading these related Biorxiv documents, it is clear that these manuscripts as a group overlap extensively with one another, even beyond the fact that they are all derived from the same underlying genome/exome data. Consider the following (non-comprehensive) examples:

While all of the manuscripts in the gnomAD package are now available on bioRxiv, they were not live (and thus couldn't be fully cited) at the time of submission of this manuscript. We respectfully disagree with the proposed examples of redundancies between the papers (see below for specific responses). In fact, we find it a strength that the dataset can be used in different fashions and achieve consistent and consistently powerful results with multiple complementary approaches.

1. LOFTEE is a core method in this manuscript, Cummings et al, and Minikel et al., being used to provide the refined data product (collections of error-depleted pLOF variants) that drives key conclusions across all three manuscripts.

In these three papers, we describe three different strategies with different datasets and audiences. In Cummings et al., we describe the use of orthogonal expression (GTEx) data to improve variant, including pLoF, annotation. This is applied to the gnomAD dataset as it is the largest genetic variant dataset in existence, but is multi-purpose and could be adapted to any expression or genetic variant dataset. The methods described in Cummings et al. have almost no overlap with the methods described here, as they relate primarily to gene expression analysis. Meanwhile, Minikel et al. uses some of the data and results described in this paper to perform a detailed exploration of the use of pLoF variation for drug target discovery and validation, which falls well outside the scope of this manuscript. The fact that the same underlying data set and harmonized quality control and filtering approaches were used in these three papers to perform conceptually distinct analyses is, we would argue, a strength of this manuscript package rather than redundancy.

2. Much of the text in Cummings et al. is thematically highly consistent with key results in this manuscript, namely expression levels and distribution in relation to pLOF variation, both real and erroneous. Note, for example, content overlap between Cummings Figure 3 and Karczewski 4b-c and overlap between Cummings Figure 4 and Karczewski 2a.

Fig. 3 in Cummings et al. describes the MAPS score for **variants** in genes falling into each LOEUF decile, which is then split out by the proportion expressed of the variants. Fig. 4b in this work describes the proportion of tissues where the genes falling into each LOEUF decile, while 4c describes the percent of expression that derives from the constrained transcript (vs unconstrained transcripts). There is no overlap in these figures except the x-axis in the former, and the words "proportion" and "expressed" in the latter.

Fig. 4 in Cummings et al. and Fig. 2a here show the proportion filtered by variant classification by different methodologies. While these are conceptually similar, showing that a method filters more common variation than gold standard disease variation is a common way to assess the performance of a metric for binary metrics (as ROC curves are for quantitative metrics).

3. Figure 1 from Minikel et al. is similar to Figure 2 in this manuscript, drawing from the same data and presenting very similar results (e.g., compare Minikel 1c with Karczewski 2c-d). Minikel Figure 1 furthermore appears to be very similar to Extended Figure 5f-h in this manuscript; all these panels are scatter plots showing observed and expected counts of variants, subset by the same variant types using the same coloring scheme, and whose key conclusion is to indicate gene or transcript-level constraint differences on different categories of variation.

Fig. 1 in Minikel et al. was indeed similar to Extended Data Fig. 5f-h, which is in turn similar to Extended Data Fig. 5 in [1]. These are meant as orienting figures to illustrate the observed and expected models, which is why they are in the Extended Data Figures for the latter two cases. Minikel et al. has now been restructured in review, and this figure has been removed from that manuscript.

4. Collins Figure 6b and Karczewski 3b both appear to use the same data and lead to similar results, namely the correlation between rates of structural variant observation and constraint on pLOF SNVs.

Indeed, these figure panels do have a substantial overlap. However, in this manuscript, the panel is intended as a high-level summary of the SV result, and to orient readers that a companion manuscript describing structural variants is available, as the focus in this manuscript is SNVs and indels. In Collins et al., the result is further expanded on in comparison to other SV types and constraint metrics in order to draw conclusions about SVs that are not relevant to this manuscript.

While these examples of overlap are not plainly duplicative of one another, they tend to provide only mildly different perspectives on the same data and ultimately lead to similar high-level conclusions. In general, there are extensive redundancies across these five manuscripts, including: shared raw, intermediate, and endpoint datasets; shared methods for variant calling and filtration; similar individual results and figures; and shared high-level conclusions.

Thus, while I understand that "lump/split" decisions for manuscripts stemming from large team-driven genomic projects can be challenging, it is my opinion that the split decisions in this case resulted in a too thin manuscript that provides only incremental impact relative to both previously published and concurrently submitted papers. However, I find it likely that a more comprehensive manuscript that combines key points here with those from the companion manuscripts would be both more reader-friendly and more impactful. It could benefit from elimination of the redundancies and better highlighting of those results which are truly new. It would also provide a more cohesive description of GnomAD, the conclusions one can derive from it, and the impact it can have as a resource.

This manuscript is intended as a flagship manuscript describing multiple advances, including of the gnomAD dataset, sample and variant filtration, variant filtration using LOFTEE, improvements to the constraint process, and LOEUF. At its current length, it already includes a full manuscript, 26 figures, 21 tables, and 80 pages of supplementary material: lumping in the additional full-length manuscripts (which each have their own message and audience) would considerably increase the length and correspondingly, diffuse the focus of this manuscript. We also note that a degree of interdependency and consistency of ideas between papers is a necessary aspect of a manuscript package.

Minor technical comments:

Additional details on the "established gene lists" that drive key results are needed. While a github link is provided, precise descriptions of how they were defined need to be in the manuscript or supplement, along with a discussion about how their ascertainment may influence the correlations and trends observed. This is particularly true to the extent that there are any manual curation steps and to the extent that there may exist implicit or explicit circularities. If, for example, data from a previous generation of ExAC were used to define a given list of genes, then the results presented here might be at least partially tautological. On a related note, who performed these curations and to what extent did they also perform the analyses presented here? I do not doubt the general veracity of these results. However, to the extent that this manuscript is refining methods/data and not providing conceptually new approaches, precisely estimating the actual magnitude of individual refinements is particularly important; thus, any relevant biases in the use of these gene lists as a measure of performance should be removed or controlled for. Ideally, gene lists defined and curated by an independent group and in the absence of ExAC data would be used as validation (e.g., those used in Petrovski et al., which predate these analyses and, I believe, the existence of ExAC as a public resource).

The gene lists used are the same as those in the ExAC paper, and thus predate the ExAC and gnomAD resources, and we have added a note to this effect in the Supplementary Information. The data were curated by other groups, and those who did the analysis here did not feed back results of these analyses to the curators.

Similar question relates to the process by which OMIM genes were defined as being discovered from WES/WGS vs linkage. Was this work done manually? How does it compare to other efforts (if any)? What about cases in which a combination of both linkage and WES/WGS were used? As per above, the effects of circularity are relevant here given the fact that ExAC has explicitly (e.g., by contributing to variant filtration) and implicitly (e.g., via use of intolerance scores in VUS evaluation) helped to identify some of the WES/WGS-discovered genes; this will likely be difficult to account for but clearly may confound the interpretation here.

The genes were automatically curated as previously described[14]. This is now clarified in the supplement: “These genes were further filtered to those causal for monogenic conditions and divided (as in Chong et al., 2015[14]) into those discovered by whole-exome/whole-genome sequencing (WES/WGS) or previous techniques, such as mapping using linkage or large recurrent chromosomal microduplication/microdeletions, followed by candidate gene sequencing.” We are aware of no other efforts to curate OMIM data in this manner (OrphaNet does not record any information about discovery). With respect to circularity, these data were not shown in the original manuscript, but we note that the decrease in LOEUF scores begins in 2012 and remains for years afterwards, predating ExAC (first released in October 2014) and especially its widespread use, as can be seen in this figure below. A further analysis of this curated dataset shows a post-WES/WGS era enrichment for gene-disease relationships attributable to *de novo* variants, supporting our claim here [Bamshad et al., 2019; AJHG in press].
[redacted]

References

1. Analysis of protein-coding genetic variation in 60,706 humans. *Nature* **536,** 285–291 (2016).

2. Samocha, K. E. *et al.* Regional missense constraint improves variant deleteriousness prediction. *bioRxiv* **380,** 148353 (2017).

3. Short, P. J., Gallone, G., Geschwind, D. H., Barrett, J. C. & Hurles, M. E. <i>De novo</i> mutations in regulatory elements in neurodevelopmental disorders. *Nature* (2018). doi:10.1038/nature25983

4. Whiffin, N. *et al.* Characterising the loss-of-function impact of 5' untranslated region variants in whole genome sequence data from 15,708 individuals. *bioRxiv* **5,** 543504 (2019).

5. Wright, C. F. *et al.* Assessing the Pathogenicity, Penetrance, and Expressivity of Putative Disease-Causing Variants in a Population Setting. *Am J Hum Genet* **104,** 275–286 (2019).

6. El-Brolosy, M. A. *et al.* Genetic compensation triggered by mutant mRNA degradation. *Nature* **568,** 193–197 (2019).

7. Tuladhar, R. *et al.* CRISPR/Cas9-based mutagenesis frequently provokes on-target mRNA misregulation. *bioRxiv* **136,** 920 (2019).

8. Cummings, B. B. *et al.* Transcript expression-aware annotation improves rare variant discovery and interpretation. *bioRxiv* 554444 (2019). doi:10.1101/554444

9. Carlston, C. M. *et al.* Pathogenic ASXL1 somatic variants in reference databases complicate germline variant interpretation for Bohring-Opitz Syndrome. *Hum Mutat* **38,** 517–523 (2017).

10. Wang, Q. *et al.* Landscape of multi-nucleotide variants in 125,748 human exomes and 15,708 genomes. *bioRxiv* **45,** 573378 (2019).

11. Collins, R. L. *et al.* An open resource of structural variation for medical and population genetics. *bioRxiv* 578674 (2019). doi:10.1101/578674

12. Quantifying unobserved protein-coding variants in human populations provides a roadmap for large-scale sequencing projects. *Nat Comms* **7,** 13293 (2016).

13. Petrovski, S., Wang, Q., Heinzen, E. L., Allen, A. S. & Goldstein, D. B. Genic Intolerance to Functional Variation and the Interpretation of Personal Genomes. *PLoS Genet* **9,** e1003709 (2013).

14. The Genetic Basis of Mendelian Phenotypes: Discoveries, Challenges, and Opportunities. *Am J Hum Genet* **97,** 199–215 (2015).

Reviewer Reports on the First Revision:

Referee #1:

Overall the authors were responsive to my comments, and the manuscript is much improved.

My remaining major comment is that I continue to struggle with LOEUF as a metric. I don't believe

that it fully sunk in for me on my first read how conflated LOEUF is with gene length. I understand why you are doing it this way (i.e. using LOEUF instead of o/e), but there needs to be more transparency and care on this point. pLI may be disguising variability in intolerance, but LOEUF is disguising gene length as a confounder on confidence, and that needs to be discussed more explicitly.

First, you need to show the relationship, e.g. as a box plot of gene lengths for LOEUF deciles. Ideally this would be a main text panel.

Second, the fact that short genes tend to be given low LOEUF scores just because they are short needs to be made more explicit. The new sentence at line 273-275 is poorly written, and practically should be its own paragraph (that cites the above requested figure). I can see low LOEUF telling you something about constraint. But high LOEUF doesn't seem to tell you much of anything, as it conflates "not under constraint" and "too short to say anything meaningful". I would really like to see a main text full paragraph acknowledging and quantifying this limitation and its consequences.

Third, you do take care to control for length in some but not all of the subsequent analyses that use LOEUF. For example, throughout Fig. 3 (and Fig. 5 as well, perhaps?; possibly other figures, I'm just using these as examples), I recognize that the result is very likely to hold up, but it seems relevant to state whether or not there are differences in the gene length distribution between the classes of genes being compared. This would also help reinforce the point to the readers that paying attention to gene length is key. The authors should carefully go through the manuscript and make sure that all LOEUF-dependent analyses control for gene length.

Minor:

79 – "many of which" should be "most of which" or "the vast majority of which".

268 – It may be worth emphasizing more that the shape of EDF7a suggests a flattish rather than dichotomous distribution of o/e, which argues for o/e (or LOUEF) over pLI.

Referee #2:

In this manuscript version, Karczewski and colleagues make substantial improvements to the original manuscript. I think the authors have done an outstanding job responding to the comments of four reviewers, who clearly all came at this manuscript with different perspectives. I am especially pleased with the quality of the figures in this version of the manuscript. They are much clearer and easier to follow. I support publication of this article. Below, I note a couple of very minor issues.

line 236: Perhaps this was just me, but I had to read this sentence a few times to grok what was going on. I think it may benefit from clarifying that the 1,555 was in all populations (I think).

ED Figs. 4 and 6: I think these figures could benefit from a bit more padding between figures. The x-axis labels on the top graphs start to blend into where titles might be for the lower graphs. It took me a few minutes to orient here, and I think a bit of extra padding would help with this.

Supplement p. 4: "We mapped reads onto the human genome build 37..." Please specify the source for this specific set of FASTAs, as well as the decoys (if any) that were used for the alignment. And, thank you for clarifying assembly name usage (GRCh37) throughout the manuscript.

Supplement p. 51: Apologies if I missed this, but I didn't see a reference to a figure or data table

this section was referencing. You seem to have this in other parts of the supplement, and it is very useful to have that reference when going through this material.

Referee #3:

In their revised manuscript the authors have addressed most of my concerns and have significantly improved their manuscript during revision. They have also addressed valuable points raised by other reviewers, which again has led to an overall improved manuscript.

In particular, they have added valuable data following my suggestions:

- Suppl. Figs. 10 and 11 to show the increased power of the current dataset;

- Suppl. Table 17 (and respective datasets) show #pLoF per individual;

- The novel analysis on CHIP (Suppl. Table 16 and accompanying datasets) are valuable new data/analysis;

- A comparison of the added value of WGS over WES even for coding regions, summarized in Suppl. Figs. 3 and 4 (not as stated in rebuttal letter SF 4 and 5); also the provided list of coverages per gene (https://storage.googleapis.com/gnomad-public/papers/2019-flagship-lof/v1.1/summary_gene_coverage/gencode_grch37_gene_by_platform_coverage_summary.tsv.gz) is valuable to the community;

- The authors have added 'allele rescue by subsequent frameshifts' to the accompanying paper on MNVs.

While the overall quality has further improved, I see further improvements opportunities, i.e. here a few suggestions for minor revisions:

- It would be interesting to state in the main text that the number of LoF variants per individual is constant since the 2012 paper. This should be further specified in: total # pLoF, of which so many common, rare and private (as now shown in Suppl. Datasets 8-9; incorrectly stated in rebuttal letter to also be contained in Suppl. Table 16 – which in fact contains the CHIP data; should be Suppl. Table 17). (refers to rebuttal point 3.)

- Concerning the CHIP analysis (Suppl. Table 16) the authors should mention that missense/activating mutations have not been subject of the current study, but are a known important contributor to the CHIP phenomenon. The authors should also clarify that the age used was 'last known age of the individual' rather than 'age at sampling'. The authors conclude that no novel genes have been identified as such strong candidates as ASXL1, DNMT3A, and TET2. This, however, is not expected, as these have been known to be the three strongest drivers of clonal hematopoiesis. The power of the current dataset, however, should pinpoint other important but less strong drivers. Can the authors comment on the genes that show significant KS test and Moods median test p-values (<1.4x10-6) but 'only' an age difference of 55 vs 50 years? These are e.g. SHROOM3, EPB41L4A, CYP4B1, AMPD1, OR5K2, ANKDD1B, FAM58A, KRTAP4-8. (refers to rebuttal point 4.)

- While I agree with the authors that true compound heterozygosity requires large-scale inference of variant phase, it is, however, safe to assume that every individual that carries 2 pLoF variants in the same gene has a 50% chance that this is in cis or trans. Already having the information whether 2 rare/private LoFs are from the same or two independent individuals can be very useful; and this could significantly enrich the list of genes for which homozygous KOs have been (never) observed. (refers to rebuttal point 6.)

- While I can understand that the power is lacking to distinguish the three classes of pLoF, adding a simple ratio of stop, fs, splice-site would be useful to the reader. (refers to rebuttal point 8.)

Referee #4:

I have read through the response to reviewers. In general, the authors have been thoughtful and responsive to reviewer comments; there are no major concerns about the technical quality of the data, and the impact of the resource as a whole has been and will continue to be high.

However, I still am not convinced that the narrow focus on LOF variation is the most effective choice for presenting this work; within the current scope of this manuscript, the conceptual novelty is minimal and the technical novelty is modest (e.g., Supp. Fig. 10). I continue to think that the key results here should be combined with the distinct key results from the other GnomAD-related papers. I simply don't agree that the three overlapping papers use "different strategies with different datasets and audiences"; the redundancies, ranging from nearly literal duplication to conceptually similar even if technically distinct, remain extensive. While I understand the authors' concern that the current manuscript is already long, my concern is not related to length but novelty and impact. Further, a combined manuscript that highlighted the truly distinct parts of each paper and collapsed the redundant components would be substantially more concise than the summed length of the current collection of manuscripts. So the net effect would be to shorten rather than extend, in addition to better highlighting the truly novel elements.

That said, the nature and structure of the Nature-published form of these manuscript(s) is an editorial consideration about which I am happy to state my opinion and move on; I don't see a need for further rounds of revision or review.

Referee #5:

The manuscript by Karczewski et al. entitled "Variation across 141,456 human exomes and genomes reveals the spectrum of loss-of-function intolerance across human protein-coding genes" describes an impressively large-scale catalog of harmonized genetic data that was used to catalog predicted loss of function (pLoF) variants that may underlie rare diseases. This review focuses more on the software and code behind the manuscript than on the manuscript itself. The authors have done a laudable job of making all the code and data publicly available and provided ample documentation; however, I have two concerns: the authors do not have a unit tests in their python- and perl-based GitHub repositories that can be used to automatically review their code, and my attempts to reproduce the figures in R were unsuccessful due to a number of warnings and errors. I believe these concerns can be quickly resolved and will greatly improve the ability of others to reuse or reproduce the data and the analyses. It is worth noting that my lack of experience using the Google Cloud Platform Dataproc cluster limited my ability test Hail and the LOFTEE software in the cloud, so an additional review by someone who is familiar using these tools on the Google platform might be worthwhile.

Specific comments

The gnomAD browser (https://gnomad.broadinstitute.org/) accompanying the manuscript has a very nice user interface. I was able to easily view pLoF and other variants in my favorite genes. This web-browser is an excellent resource for those who wish to use a GUI to explore the data (e.g. clinicians, teachers, students, members of the who lack the computational expertise to sift through the raw data).

As described in the manuscript, all data processing and analyses were performed using Hail

(https://hail.is/), which is an open-source, Python-based library. The documentation for Hail 0.2 is very thorough, and I was able to successfully follow the local installation instructions and the GWAS tutorials with relative ease. This speaks very well for the potential to reproduce the analyses described in the manuscript. However, I was not able to install Hail on the HPC system I normally use (Stampede 2 at the Texas Advanced Computing Facility) nor was I able to install it the Cloud Platform Dataproc cluster (https://cloud.google.com/dataproc/) used by the authors. I am a first time Google Cloud user, so this doesn't really surprise me.

The hyperlink on p. 30 is a dead end. "The filtering frequency described previously13 is implemented in Hail (https://hail.is/docs/0.2/experimental.html#hail.experimental.filtering_allele_frequency).

In addition to the detailed supplementary materials, some of the co-authors wrote a blog post (https://macarthurlab.org/2018/10/17/gnomad-v2-1/) that provides a detailed walk-through of the scripts and the variables used to generate many of the figures in the manuscript. This is also a valuable resource for anyone wishing to reproduce the analyses.

I am concerned that none of the three repositories listed in the "code and software checklist" have clearly marked tests that could be used to automate the process of code review. I did find tests in https://github.com/macarthur-lab/gnomad_hail; however, this repository was not listed as critical to the manuscript. Also, it appears that these error messages are sent to a slack channel, which would be highly useful if you were a member of the slack channel but not so useful to someone outside the McArthur lab group. I am aware that the authors consider these repositories to be a collection of scripts rather than a software package; however, because the README files encourage others to use and modify the code, it would be very useful if the authors could add continuous integration (like Travis-CI (https://travis-ci.com/), which would allow automated testing when changes to code are made, and the addition of a badge (or shield) to the repo's README would give new users confidence that the code is working as expected.

The `gnomad_qc` repository is well organized, and the functions are well documented. This workflow describes in the repository corresponds nicely to the "Sample QC" section of the supplementary materials, so I could identify which functions correspond to steps outlined in the methods section. This repository also corresponds to ED Fig. 1, but I find this figure to be more confusing than helpful. It's not immediately obvious that the terse bullet points map onto the arrows between boxes. It would be more useful if panel 1a was broken down into panels 1a-g and if each arrow was labelled with the function(s) that is used to perform that action. By giving each step its own label, you can remove the text in the middle and more precisely refer to read to that specific part of the figure when describing the workflow in the methods section.

In `gnomad_qc/sample_qc/apply_hard_filters.py` on line 13, the authors use "cutoff of F<0.5 for females and F>0.8 for males for genomes"; however, on p. 8 of the suppl. methods, the authors state "For genomes… samples with F > 0.8 were classified as male and samples with F < 0.2 were classified as female." Which is correct: 0.2 or 0.5 for females?

In `gnomad_qc/sample_qc/apply_hard_filters.py` on line 31, the authors refer to a metadata file that by given to them by a colleague. Is this metadata public? Can it be referred to by a DOI? How does this comment about the peculiarity of the metadata affect the ability for someone else to remix or reuse this pipeline?

The R code in `gnomad_lof/R` is also well written and well documented. I especially like that all the libraries, custom aesthetics, custom functions, and aliases use are located in `constants.R`. This file does need to be sourced in `all_figures.R` for the contents to be loaded in the environment before generating the figures.

Of the 30+ R packages used, I had to install about 10 libraries. One way to make this repository

more reproducible would be to use Binder to create a shareable, interactive environment in the cloud following the instructions described at https://mybinder.readthedocs.io/en/latest/sample_repos.html#specifying-an-r-environment-with-a-runtime-txt-file.

`figure1()` did not return a figure. The error message was:

```
Error in download.file(url, fname) :
cannot open URL 'https://storage.googleapis.com/gnomad-public/papers/2019-flagship-lof/v1.0/summary_results/observed_possible_expanded_exomes.txt.bgz'
```

I went directly to that link in a browser and was told there was "No such object".

`figure2()`, `efigure5()`, `efigure6()` did return pdf files, but they had not content. There was no

`figure3()` did not return a figure. The error and warning messages were `Error: `by` required, because the data sources have no common variables` and `Unknown levels in `f`: all_ar, all_ad`. For this figure, I was able to manually run the code inside the functions well enough to partially generate Fig. 3a (because for some reason my gene_list only contains olfactory genes, so they were the only genes plots, rather than all three). Additionally, I think traced the error to `left_join(load_all_gene_list_data())` because, in my environment, both `gene_data` and `gene_lists` have a column called `gene` that could be used for joining, but something is going awry.

`figure4()` did not return a figure but did return a few warning messages. There first said "we couldn't map to STRING 0% of your identifiers`, which I think means all 100% of the strings were mapped, but the double negative is a little confusing. The second message occurred twice and said, "At centrality.c:2784 :closeness centrality is not well-defined for disconnected graphs".

`figure5()` almost caused R studio to crash (as predicted in the README), but my session powered through. I did get a pop-up message saying "some updates could not be installed because RStudio interrupted restart. I also don't know how to interpret this, but it might help you debug. As with Fig. 2, a pdf file was created but there were no pages.

`efigure2()` and `efigure3()` ran successfully and produced png and pdf files that look exactly like the figures in the manuscript. I also like that the authors use `ggarrange()` and `get_legend` to reproducibly label and arrange the figures and create a shared legend. On that note, I checked to see base R and/or all the R packages used were cited, and I did not find any citations for the software. I don't believe that there is a page limit to the supplementary materials, so it would be nice to acknowledge these open-source software packages. You can get these from the command line using, for example, `citation("cowplot")`.

I could not source "efig4_downsamplings.R" because https://storage.googleapis.com/gnomad-public/papers/2019-flagship-lof/v1.0/summary_results/observed_possible_expanded_exomes.txt.bgz was not found.

`source('efig7_constraint.R')` overloaded my ram to the extent that I couldn't even use the mouse or keyboard to quit R, so I chose to shut down my computer after a few minutes of listening to it work at max capacity. This is not good. One solution is to put a disclaimer in a comment next to this line to warn the user. A second solution would be to optimize the code, but I do not have a suggestion.

`source('efig8_biology.R')` returned the following error: `Error in .local(drv, ...) : Failed to

connect to database: Error: Unknown database 'tcrd520'` .

The GitHub repository `konradjk/loftee` contains the perl-based, Loss-Of-Function Transcript Effect Estimator (LOFTEE) package or Hail plugin that is used to filter and flag Loss-of-function mutations in conjunction with a clinical variation (ClinVar) dataset containing four hundred thousand variants and a SNV dataset with eight billion variants to annotate the 125,748 exomes. I do not know how to read PERL, so I can't evaluate the quality of the code, but the repo is well organized, and the README thoroughly describes the functions. Given the requirements (SAMtools, human genome, PhyloCSF) I did not even attempt to install the software locally. I usually run SAMtools on Stampede 2 at the Texas Advanced Computing, so I was going to test it there, but I couldn't install HAIL on Stampede. I also attempted to test Hail and LOFTEE on the Google Cloud Platform Dataproc cluster, but I'm a first-time user, and I wasn't able to overcome installation problems. For review purposes, it would be ideal if someone with Google Cloud expertise reviewed the software. For training purposes, creating a tutorial (video or blog post) about how to use LOFTEE with Hail on Google would be very valuable to those looking to use these tools for new analyses or to reproduce the analyses described in Karczewski et al.

Author Rebuttals to First Revision:

We thank all five reviewers for their comments, and have addressed them below. We believe this has improved the manuscript and especially the usability of the code now and for future projects.

In the process of manuscript revisions we identified an issue with undercalling of some homozygous genotypes due to low levels of contamination in a subset of gnomAD individuals. This issue, which affects a small fraction of genotypes at <2% of the variant sites in gnomAD, is spelled out in more detail in a separate preprint [Karczewski et al., 2019]. The affected variants have been flagged in the gnomAD browser and data release, and permanent fixes will be made in a future gnomAD release.

While a complete fix would require reprocessing the entire gnomAD data set, which is not viable for this manuscript release, we have thoroughly reviewed the impact of this error mode on all of the analyses presented in the gnomAD preprints. Fortunately, since the vast majority of our analyses rely only on site accuracy (which is unaffected) or allele frequencies (which are only very slightly altered), this impact is extremely modest. We have added a caveat to the two analyses that are non-trivially affected by this error mode: the curation of homozygous LoF-tolerant genes (for which some true LoF-tolerant genes may have been missed) and the generation of the composite LoF allele frequency, or CAF, genome-wide (will have been very slightly underestimated at a subset of genes). We believe the remainder of the paper is not materially affected by this error mode.

Referee #1 (Remarks to the Author):

Overall the authors were responsive to my comments, and the manuscript is much improved.

My remaining major comment is that I continue to struggle with LOEUF as a metric. I don't believe that it fully sunk in for me on my first read how conflated LOEUF is with gene length. I understand why you are doing it this way (i.e. using LOEUF instead of o/e), but there needs to be more transparency and care on this point. pLI may be disguising variability in intolerance but LOEUF is disguising gene length as a confounder on confidence, and that needs to be discussed more explicitly.

We thank the reviewer for these comments - unfortunately, all constraint metrics are confounded at least in some part by gene length. To make this clear for readers, we have added the relationship between LOEUF and gene length as an Extended Data Figure panel, clarified this in the text, and repeated all analyses with coding sequence length as a covariate, which have all remained highly significant.

First, you need to show the relationship, e.g. as a box plot of gene lengths for LOEUF deciles. Ideally this would be a main text panel.

We have now added this as a figure panel to Extended Data Fig. 7, as we don't have any main figures describing the technical process of LOEUF.

Second, the fact that short genes tend to be given low LOEUF scores just because they are short needs to be made more explicit. The new sentence at line 273-275 is poorly written, and practically should be its own paragraph (that cites the above requested figure). I can see low LOEUF telling you something about constraint. But high LOEUF doesn't seem to tell you much of anything, as it conflates "not under constraint" and "too short to say anything meaningful". I would really like to see a main text full paragraph acknowledging and quantifying this limitation and its consequences.

We have added a new paragraph to discuss this caveat. We have also added some text to the supplement discussing Extended Data Fig. 7d, outlining the approaches we've taken to reduce the impact of confounding by gene length on the analyses throughout the paper (described in more detail below): "LOEUF is correlated with coding sequence length (beta = -1.07×10^{-4}; $p < 10^{-100}$; Extended Data Fig. 7d): as a result, we have adjusted for gene length or removed genes with fewer than 10 expected pLoFs in all analyses."

The new paragraph reads:

"We note that the use of the upper bound means that LOEUF is a conservative metric in one direction: genes with low LOEUF scores are confidently depleted for pLoF variation, whereas genes with high LOEUF scores are a mixture of genes without depletion, and genes that are too small to obtain a precise estimate of the o/e ratio. In general, however, the scale of gnomAD means that gene length is rarely a substantive confounder for the analyses described here, and all downstream analyses are adjusted for coding sequence length or filtered to genes with at least 10 expected pLoFs (see Supplementary Information)."

Third, you do take care to control for length in some but not all of the subsequent analyses that use LOEUF. For example, on throughout Figure 3 (and Figure 5 as well, perhaps?; possibly other figures, I'm just using these as examples), I recognize that the result is very likely to hold up, but it seems relevant to state whether or not there are differences in the gene length distribution between the classes of genes being compared. This would also help reinforce the point to the readers that paying attention to gene length is key. The authors should carefully go through the manuscript and make sure that all LOEUF dependent analyses control for gene length.

This is entirely fair - our early analyses made us confident that the LOEUF results described in the paper weren't driven by confounding, but this was not adequately formally demonstrated in the paper. We have now reviewed all analyses described in the paper to investigate any impact of confounding by gene length. Our overall finding is that while gene length is indeed correlated with a variety of biological metrics, the correlations between LOEUF and these metrics is generally far stronger, and is not materially driven by gene length confounding. We have now added a coding sequence length adjustment to the supplement for the main and extended data figures where appropriate. These additions are enumerated below:

Fig. 3a: There was no statistical test done previously, but we have now added one to the supplement: "Membership in the haploinsufficient gene class is highly predicted by LOEUF (logistic regression beta = -4.3; p = 1.57 x 10^{-33}), even when adjusted for coding sequence length (p = 0.18 for the contribution of gene length in the joint model). Likewise, membership in the olfactory gene class is positively correlated with LOEUF (logistic regression beta = 3.4; p = 2.5 x 10^{-85}), even when adjusted for gene length (p = 0.023 for the contribution of gene length in the joint model)."

Fig. 3b: We have now adjusted for gene length and added this to the supplement: "The SV-derived observed:expected ratios are correlated with LOEUF (r = 0.13; p = 3.5 x 10^{-71}), after adjusting for gene length (p = 7.5 x 10^{-6} for the contribution of gene length)."

Fig. 3c-d: We have added statistical tests to the supplement, adjusting for gene length, for the mouse knockout and cell essential/non-essential genes: "Overlap with mouse heterozygous lethality

was significantly associated with LOEUF (logistic regression beta = -2.27; $p = 3.3 \times 10^{-52}$), even when adjusted for coding sequence length (beta = 3.3×10^{-5}; $p = 0.028$). LOEUF is also correlated with cell essentiality (logistic regression beta = -1.71; $p = 1.7 \times 10^{-65}$; coding sequence length: beta = 2.5×10^{-4}; $p = 2.4 \times 10^{-12}$) and non-essentiality (beta = 1.45; $p = 3.8 \times 10^{-71}$; coding sequence length: beta = -5.9×10^{-6}; $p = 0.84$)."

Fig. 4b: We have added to the supplement: "Overall, the number of tissues in which a canonical transcript is expressed is correlated with LOEUF (linear regression beta = -1.07; $p < 10^{-100}$) when adjusted for gene length (beta = -9.9×10^{-4}; $p = 10^{-53}$ for the contribution of gene length)."

Fig. 5a and Extended Data Fig. 9c: In this analysis, we have clarified in the supplement that "Genes were filtered to those with at least 10 expected pLoF variants."

Extended Data Fig. 8a: We had not previously performed an explicit statistical test for this analysis, but we have now added a set of tests for each category: "Each of these categories is significantly correlated with LOEUF in a joint logistic regression model with coding sequence length: Tclin (beta = -0.78; $p = 4 \times 10^{-18}$; cds length: beta = 2×10^{-6}; $p = 0.89$), Tchem (beta = -0.63; $p = 8 \times 10^{-30}$; cds length: beta = 5×10^{-6}; $p = 0.68$), Tbio (beta = -0.99; $p < 10^{-100}$; cds length: beta = 1.6×10^{-5}; $p = 0.07$), Tdark (beta = 1.17; $p < 10^{-100}$; cds length: beta = 2.7×10^{-5}; $p = 0.009$)."

Extended Data Fig. 8b: In a similar fashion to Fig. 4b, we repeated this analysis for all transcripts: "Similarly, the number of tissues in which a transcript is expressed is correlated with the transcript's LOEUF (linear regression beta = -5.2; $p < 10^{-100}$) when adjusted for gene length (beta = -9.4×10^{-5}; $p = 0.01$ for the contribution of gene length)."

Extended Data Fig. 9a: We have added a logistic regression model to the supplement for OMIM vs LOEUF adjusting for gene length: "In a logistic regression model with coding sequence length as a covariate, LOEUF is correlated with OMIM status (beta = -0.69; $p = 4 \times 10^{-61}$; gene length beta = 1.3×10^{-4}; $p = 1.2 \times 10^{-33}$)."

Extended Data Fig. 9b: We have added a logistic regression model to the supplement for NGS status vs LOEUF adjusting for gene length: "Within OMIM genes, LOEUF is correlated with discovery by WES/WGS compared to conventional approaches (beta = -0.69; $p = 2 \times 10^{-14}$) when adjusting for coding sequence length (beta = 7.8×10^{-6}; $p = 0.54$ for the contribution of gene length). "

Extended Data Fig. 9d: We have now filtered this analysis to include only genes with at least 10 pLoF variants expected, which is now properly described alongside Fig. 5a and Extended Data Fig. 9c.

Supplementary Fig. 10a-b: We have added a joint logistic regression model to the supplement: “In the logistic regression, LOEUF is highly correlated with membership in the haploinsufficient (beta = -2.6; $p = 4 \times 10^{-5}$) and essential (beta = -1.4; $p = 1.9 \times 10^{-25}$) gene lists, in a joint model with pLI (beta = 1.5; $p = 3 \times 10^{-4}$ and beta = 0.17; p = 0.15, respectively), RVIS (beta = -0.18; p = 0.05, and beta = -0.19; $p = 1.5 \times 10^{-5}$, respectively), and coding sequence length (beta = 4×10^{-6}; p = 0.92 and beta = -8×10^{-5}; $p = 7 \times 10^{-4}$, respectively).”

Minor:

79 – “many of which” should be “most of which” or “the vast majority of which”

We have modified this statement to be “most of which”.

268 – It may be worth emphasizing more that the shape of EDF7a suggests a flattish rather than dichotomous distribution of o/e, which argues for o/e (or LOUEF) over pLI

We have added a clause in the main text “that the distribution of o/e is not dichotomous, but continuous”.

Referee #2 (Remarks to the Author):

In this manuscript version, Karczewski and colleagues make substantial improve- ments to the original manuscript. I think the authors have done an outstanding job responding to the comments of 4 reviewers who clearly all came at this manuscript with different perspectives. I am especially pleased with the quality of the figures in this version of the manuscript. They are much clearer and easier to follow. I support publication of this article. Below, I note a couple of very minor issues.

line 236: perhaps this was just me, but I had to read this sentence a few times to grok what was going on. I think it may benefit from clarifying that the 1,555 was in all populations (I think).

We have changed "have an aggregate pLoF frequency of at least 0.1%" to "have an aggregate pLoF frequency at least 0.1% across all individuals in the dataset".

EDF 4 and EDF 6: I think these figures could benefit from a bit more padding between figures. The X-axis labels on the top graphs start to blend into where titles might be for the lower graphs. It took me a few minutes to orient here- and I think a bit of extra padding would help with this.

We have added more padding in Extended Data Figures 4 and 6.

Supplemental page 4: . "We mapped reads onto the human genome build 37. . . ". Please specify the source for this specific set of FASTAs, as well as the decoys (if any) that were used for the alignment. And, thank you for clarifying assembly name usage (GRCh37) throughout the manuscript.

We have added more description on the reference genome to the supplement: "The FASTA file can be found at ftp.ncbi.nlm.nih.gov/sra/reports/Assembly/GRCh37-HG19_Broad_variant/Homo_sapiens_assembly19.fasta, which has 85 contigs including a decoy (NC_007605, 171823bp)."

Supplemental page 51: Apologies if I missed this, but I didn't see a reference to a figure or data table this section was referencing. You seem to have this in other parts of the supplement, and it is very useful to have that reference when going through this material.

We apologize for not including a reference to this Figure. We have now edited the text and added the data file as Supplementary Dataset 13: "Supplementary Fig. 9 enumerates all the genes in Supplementary Table 18, which are also available as Supplementary Dataset 13."

Referee #3 (Remarks to the Author):

In their revised manuscript the authors have addressed most of my concerns and have significantly improved their manuscript during revision. They have also addressed valuable points raised by other reviewers, which again has led to an overall improved manuscript.

In particular, they have added valuable data following my suggestions:

- Supplementary Fig 10 and 11 to show the increased power of the current dataset.

- Supplementary Table 17 (and respective datasets) show #pLoF per individual.

- The novel analysis on CHIP (Supplementary Table 16 and accompanying datasets) are valuable new data/analysis.

- A comparison of the added value of WGS over WES even for coding regions, summarized in Supplementary Figures 3 and 4 (not as stated in rebuttal letter SF 4 and 5); also the provided list of coverages per gene (https://storage.googleapis.com/gnomad-public/papers/2019-flagship-lof/v1.1/summary_gene_coverage/gencode_grch37_gene_by_platform_coverage_summary.tsv.gz) is valuable to the community.

- The authors have added 'allele rescue by subsequent frameshifts' to the accompanying paper on MNVs.

While the overall quality has further improved, I see further improvements opportunities, i.e. here a few suggestions for minor revisions:

- It would be interesting to state in the main text that the number of LoF variants per individual is constant since the 2012 paper. This should be further specified in: total # pLoF, of which so many common, rare and private (as now shown in Suppl. Datasets 8-9; incorrectly stated in rebuttal letter to also be contained in Supplementary Table 16 – which in fact contains the CHIP data; should be Supplementary Table 17). (refers to rebuttal point 3.))

We have added a note to this effect in the main text ("The number of pLoF variants per individual is consistent with previous reports[3], and is highly dependent on frequency filters chosen (Supplementary Table 17)."), and expanded Supplementary Table 17 to include filters for rare and private variants.

- Concerning the CHIP analysis (Supplementary Table 16) the authors should mention, that missense/activating mutations have not been subject of the current study, but are a known important contributor to the CHIP phenomenon. The authors should also clarify that the age used was 'last known age of the individual' rather than 'age at sampling'. The authors conclude that no novel genes have been identified as such strong candidates as ASXL1, DNMT3A and TET2. This is however not expected, as these have been known to be the three strongest drivers of clonal hematopoiesis. The power of the current dataset however should pinpoint other important but less strong drivers. Can the authors comment on the genes that show significant KS test and Moods median test p-values (<1.4x10-6) but 'only' an age difference of 55 vs 50 years? These are e.g. SHROOM3, EPB41L4A, CYP4B1, AMPD1, OR5K2, ANKDD1B, FAM58A, KRTAP4-8. (refers to rebuttal point 4.))

We have added a discussion point to the supplement that “We focused our analysis on signals of pLoF variants though notably, CHIP can also be characterized by the accumulation of missense variants which would not have been revealed using our methods; future work to filter high-impact missense variants will enable a more complete understanding of CHIP.” Additionally, we have added clarification that “Cohorts vary in their reporting of age information. For example, some report age at diagnosis whereas others report the age at of the last patient visit. Age is therefore defined as the last known age of the individual and is not necessarily the age at sampling.” and have added a note to the Table legend to mention that this is the “last known age of the individual.” With respect to the genes that are significant but with small effect size, we note that residual technical artifacts (especially annotation errors at common pLoF variants) may skew the distributions and result in an inflated significance with small effect, and thus, wanted to focus on the genes with high impact. However, we release the full dataset as Supplementary Dataset 6 with all summary statistics and p-values for others to explore further.

- While I agree with the authors that true compound heterozygosity requires large-scale inference of variant phase. It is however safe to assume that every individual that carries 2 pLoF variants in the same gene has a 50% that this is in cis or trans. Already having the information whether 2 rare/private LoFs are from the same or two independent individuals can be very useful; and this could significantly enrich the list of genes for which homozygous KOs have been (never) observed. (refers to rebuttal point 6.))

We are also interested in this question, but it would require extensive analysis to properly address, and will also benefit substantially from later gnomAD versions with larger numbers of whole genomes. As such, we believe that this work falls beyond the scope of this manuscript and will need to be a future focus.

- While I can understand that the power is lacking to distinguish the three classes of pLoF; adding a simple ratio of stop, fs, splice-site would be useful to the reader. (refers to rebuttal point 8.)).

We have now added the distribution of these classes of variation in Supplementary Table 17.

Referee #4 (Remarks to the Author):

I have read through the response to reviewers. In general, the authors have been thoughtful and responsive to reviewer comments, there are no major concerns about the technical quality of the data, and the impact of the resource as a whole has been and will continue to be high.

However, I still am not convinced that the narrow focus on LOF variation is the most effective choice for presenting this work; within the current scope of this manuscript, the conceptual novelty is minimal and the technical novelty is modest (e.g., Supp Fig 10). I continue to think that the key results here should be combined with the distinct key results from the other GnomAD-related papers. I simply don't agree that the three overlapping papers use "different strategies with different datasets and audiences"; the redundancies, ranging from nearly literal duplication to conceptually similar even if technically distinct, remain extensive. While I understand the authors' concern that the current manuscript is already long, my concern is not related to length but novelty and impact. Further, a combined manuscript that highlighted the truly distinct parts of each paper and collapsed the redundant components would be substantially more concise than the summed length of the current collection of manuscripts. So the net effect would be to shorten rather than extend, in addition to better highlighting the truly novel elements.

That said, the nature and structure of the Nature-published form of these manuscript(s) is an editorial consideration about which I am happy to state my opinion and move on; I don't see a need for further rounds of revision or review.

Referee #5 (Remarks to the Author):

The manuscript by Karczewski et al. entitled "Variation across 141,456 human exomes and genomes reveals the spectrum of loss-of-function intolerance across human protein-coding genes" describes an impressively large-scale catalog of harmonized genetic data that was used to catalog predicted loss of function (pLoF) variants that may underlie rare diseases. This review focuses more on the software and code behind the manuscript than on the manuscript itself. The authors have done a laudable job of making all the code and data publicly available and provided ample documentation; however, I have two concerns: the authors do not have a unit tests in their python- and perl-based GitHub repositories that can be used to automatically review their code, and my attempts to reproduce the figures in R were unsuccessful due to a number of warnings and errors. I believe these concerns can be quickly resolved and will greatly improve the ability of others to reuse or reproduce the data and the analyses. It is worth noting that my lack of experience using the Google Cloud Platform Dataproc cluster limited my ability test Hail and the LOFTEE software in the cloud, so an additional review by someone who is familiar using these tools on the Google platform might be worthwhile.

We thank the new reviewer for their comments on the code. We have fixed the code and file hosting so that all the figures may be reproduced, except Extended Data Figure 9 for which we could not share some external data files. We have additionally created a Docker image (konradjk/gnomad_lof_paper:0.2) and verified that it also recreates all the figures. We address the comment about unit tests below.

Specific comments

The gnomAD browser (https://gnomad.broadinstitute.org/) accompanying the manuscript has a very nice user interface. I was able to easily view pLoF and other variants in my favorite genes. This web-browser is an excellent resource for those who wish to use a GUI to explore the data (e.g. clinicians, teachers, students, members of the who lack the computational expertise to sift through the raw data).

As described in the manuscript, all data processing and analyses were performed using Hail (https://hail.is/), which is an open-source, Python-based library. The documentation for Hail 0.2 is very thorough, and I was able to successfully follow the local installation instructions and the GWAS tutorials with relative ease. This speaks very well for the potential to reproduce the analyses described in the manuscript. However, I was not able to install Hail on the HPC system I normally use (Stampede 2 at the Texas Advanced Computing Facility) nor was I able to install it the Cloud Platform Dataproc cluster (https://cloud.google.com/dataproc/) used by the authors. I am a first time Google Cloud user, so this doesn't really surprise me.

The hyperlink on page 30 is a dead end. "The filtering frequency described previously13 is implemented in Hail (https://hail.is/docs/0.2/experimental.html#hail.experimental.filtering_allele_frequency)

This has now been fixed to:
https://hail.is/docs/0.2/experimental/index.html#hail.experimental.filtering_allele_frequency

In addition to the detailed supplementary materials, some of the co-authors wrote a blog post (https://macarthurlab.org/2018/10/17/gnomad-v2-1/) that provides a detailed walk-through of the scripts and the variables used to generate many of the figures in the manuscript. This is also a valuable resource for anyone wishing to reproduce the analyses.

We thank the reviewer for their comments, and are glad the browser and blog posts have been useful.

I am concerned that none of the three repositories listed in the "code and software checklist" have clearly marked tests that could be used to automate the process of code review. I did find tests in https://github.com/macarthur-lab/gnomad_hail; however, this repository was not listed as critical to the manuscript. Also, it appears that these error messages are sent to a Slack channel, which would be highly useful if you were a member of the slack channel but not so useful to someone outside the McArthur lab group. I am aware that the authors consider these repositories to be a collection of scripts rather than a software package; however, because the README files encourage others to use and modify the code, it would be very useful if the author could add continuous integration (like Travis-CI (https://travis-ci.com/) which would allow automated testing when changes to code are made, and the addition of a badge (or shield) to the repo's README would give new users

confidence that the code is working as expected.

We agree that unit tests are extremely important for production code, but a few things conspire to make this difficult. First and foremost, as we continue to build new large datasets, we are constantly working on making these functions more generic, and thus, their interface is regularly changing. These repos represent a snapshot of the current analysis, but we are factoring out many to create a generalizable toolkit for large-scale data analysis in Hail. We are building this out as we go, but it is a substantial effort that we will not be able to do in a reasonable timeframe for this manuscript. Many of the specific functions would require large test datasets (e.g. run_pca_with_relateds would require a full dataset), and so, are difficult to write comprehensive tests, and similarly, many of the functions require sample-level metadata that cannot be shared.

Note that while we did not write unit tests for these scripts, we did run many sanity checks on the data that was released, which is provided in `prepare_data_release.py` which was a form of test on the release file that helped us catch many bugs along the way. In particular, we checked the following:

- The fraction of filtered variants, broken down by allele type (SNV, indel) and site type (bi-allelic, multi-allelic), followed our general expectations (overall filtering numbers, SNV generally more confident than indels and bi-allelic sites overall more confident than multi-allelic sites.
- For all samples and for each of the subsets we created, that the allele count and allele number (and by definition allele frequency) for unfiltered samples was greater than 0 for all variants and was always smaller or equal to that of filtered samples.
- That the sum of allele count, allele number and number of homozygotes for all populations equals the total allele count, allele number and number of homozygotes respectively.
- For all samples, subsets and each (sub)population, that:
    - Allele count in males + allele count in females = total allele count

    - Allele number in males + allele number in females = total allele number
    - Homozygote count in males + Homozygote count in females = total Homozygote count
- That allele count and allele number on the Y chromosome were all 0 in females
- That all males were counted as hemizygous on the non-pseudoautosomal parts of chromosomes X and Y
- That all the quality metrics we annotated the data with did not have unexpected missingness

In summary, we agree that unit tests are valuable, and plan to incorporate these into future versions of the pipeline, but do not believe that it is necessary or feasible to include them in the codebase for this manuscript.

The gnomad_qc repository is well organized, and the functions are well documented. This workflow describes in the repository corresponds nicely to the "Sample QC" section of the supplementary materials, so I could identify which functions correspond to steps outlined in the methods section. This repository also corresponds to extended Data Figure 1, but I find this figure to be more confusing than helpful. It's not immediately obvious that the terse bullet points map onto the arrows between boxes. It would be more useful if panel 1a was broken down into panels 1a-g and if each arrow was labelled with the function(s) that is used to perform that action. By giving each step its own label, you can remove the text in the middle and more precisely refer to read to that specific part of the figure when describing the workflow in the methods section.

We thank the reviewer for this idea - we have added a mapping between the steps in Extended Data Fig. 1a and the sample QC code in the Supplementary information, and believe this has clarified our process:

"The pipeline is available in its entirety at https://github.com/macarthur-lab/gnomad_qc and is summarized in Extended Data Fig. 1a, where numbered steps correspond to the following scripts in the code repository:

1. Hard filtering: apply_hard_filters.py
2. Relatedness inference: joint_sample_qc.py
3. Ancestry inference: joint_sample_qc.py, assign_subpops.py
4. Platform inference: exomes_platform_pca.py
5. Population- and platform-specific outlier filtering: joint_sample_qc.py
6. Finalizing release callset: finalize_sample_qc.py"

In gnomad_qc/sample_qc/apply_hard_filters.py on line 13, the authors use a "cutoff of F<0.5 for females and F>0.8 for males for genomes"; however, on page 8 of the supplementary methods, the

authors state “For genomes... samples with F > 0.8 were classified as male and samples with F < 0.2 were classified as female.” Which is correct: 0.2 or 0.5 for females?

Thank you for catching this - indeed it should be 0.5 and this has now been fixed in the supplementary text.

In gnomad_qc/sample_qc/apply_hard_filters.py on line 31, the authors refer to a metadata file that by given to them by a colleague. Is this metadata public? Can it be referred to by a DOI? How does this comment about the peculiarity of the metadata affect the ability for someone else to remix or reuse this pipeline?

Unfortunately, this metadata file contains sample-level information which cannot be released to the public. This part of the code is provided only for reference and would need to be edited for use by others as it depends on the particular upstream processing steps of the QC pipeline.

The R code in gnomad_lof/R is also well written and well documented. I especially like that all the libraries, custom aesthetics, custom functions, and aliases use are located in constants.R. This file does need to be sourced in all_figures.R for the contents to be loaded in the environment before generating the figures.

Of the 30+ R packages use, I had to install about 10 libraries. One way to make this repository more reproducible would be to use Binder to create a shareable, interactive environment in the cloud following the instructions described at https://mybinder.readthedocs.io/en/latest/sample_repos.html#specifying-an-r-environment-with-a-runtime-txt-file

We thank the reviewer for this suggestion to ensure a reproducible environment. We have updated all the code to use the newest versions of all included libraries and have also created a Docker image to ensure that the code and data are in the same place and produce the desired output. We have tested this Docker on a Macbook Pro with 16 GB of RAM and it now creates all the figures without error.

figure1() did not return a figure. The error message was:

Error in download.file(url, fname) :

cannot open URL ' https://storage.googleapis.com/gnomad-public/papers/2019-flagship-lof/v1.0/summary_results/observed_possible_expanded_exomes.txt.bgz '

I went directly to that link in a browser and was told there was “No such object”.

We apologize for the inconvenience. We had identified a minor issue with this file, regenerated it, and moved it over to a new versioned directory. We have now updated the code to auto-detect which version is available and this should work now with the newest one.

figure2(), efigure5(), efigure6() did return pdf files, but they had not content. There were no error messages in the standard output that I could use to debug.

We apologize for the lack of figures generated. When the commands are run one-by-one, output is created, but the wrapper functions and scripts are missing crucial print functions that would generate the output when `source`d. We have now added these and this should generate output for all figures.

`figure3()` did not return a figure. The error and warning messages were `Error: `by` required, because the data sources have no common variables` and `Unknown levels in `f`: all_ar, all_ad`. For this figure, I was able to manually run the code inside the functions well enough to partially generate figure 3a (because for some reason my gene_list only contains olfactory genes, so they were the only genes plots, rather than all three). Additionally, I think traced the error to `left_join(load_all_gene_list_data())` because, in my environment, both `gene_data` and `gene_lists` have a column called `gene` that could be used for joining, but something is going awry.

This function relied on a specific location of gene list data, and silently produced no output rather than erroring(!). We have now added some of the gene lists to the repo, and added a call to download the others if they don’t exist. Thank you for spotting this.

figure4() did not return a figure but did return a few warning messages. There first said “we couldn’t map to STRING 0% of your identifiers”, which I think means all 100% of the strings were mapped, but the double negative is a little confusing. The second message occurred twice and said, “At centrality.c:2784 :closeness centrality is not well-defined for disconnected graphs”.

This figure should work now as well (the warnings are from STRINGdb and are unrelated).

figure5() almost caused R studio to crash (as predicted in the README), but my session powered through. I did get a pop-up message saying “some updates could not be installed because RStudio interrupted restart. I also don’t know how to interpret this, but it might help you debug. As with figure 2, a pdf file was created but there were no pages.

This one is a mystery to us. We have fixed the printing issue, but we do not see the update message. This function also works inside the Docker.

efigure2() and efigure3() ran successfully and produced png and pdf files that look exactly like the figures in the manuscript. I also like that the authors use ggarrange() and get_legend to reproducibly label and arrange the figures and create a shared legend. On that note, I checked to see base R and/or all the R packages used were cited and I did not find any citations for the software. I don’t believe that there is a page limit to the supplementary materials, so it would be nice to acknowledge these open-source software packages. You can get these from the command line using, for example, citation("cowplot").

We have enumerated and added citations for many of the packages used: “All analyses were done using R 3.6.1 with packages including tidyverse[62], broom[63], magrittr[64], readxl[65], plotROC[66], meta[67], STRINGdb[68], and tidygraph[69]. All visualizations were plotted in ggplot2[70], and aided by scales[71], ggridges[72], egg[73], ggpubr[74], ggrastr[75], cowplot[76], ggrepel[77], and ggwordcloud[78].”

I could not source “efig4_downsamplings.R” because https://storage.googleapis.com/gnomad-public/papers/2019-flagship-lof/v1.0/summary_results/observed_possible_expanded_exomes.txt.bgz was not found.

The fix applied for Figure 1 has also fixed this one as well.

source('efig7_constraint.R') overloaded my ram to the extent that I couldn’t even use the mouse our keyboard to quit R, so I chose to shut down my computer after a few minutes of listening to it work at max capacity. This is not good. One solution is to put a disclaimer in a comment next to this line to warn the user. A second solution would be to optimize the code, but I do not have a suggestion.

We have reduced the heavy computational burden by pre-computing the metrics in this script. We now load that data explicitly (but have retained the initial metric-generation code for reference).

source('efig8_biology.R') returned the following error: Error in .local(drv, ...) : Failed to connect to database: Error: Unknown database 'tcrd520'.

We have replicated this error. The Pharos database was updated in the meantime and we had hard-coded the version available at the time. We have instead downloaded the data needed into the repo to avoid the dependence on this database (and left the download code as-is for future reference).

The GitHub repository `konradjk/loftee` contains the perl-based, Loss-Of-Function Transcript Effect Estimator (LOFTEE) package or Hail plugin that is used to filter and flag Loss-of-function mutations in conjunction with a clinical variation (ClinVar) dataset containing four hundred thousand variants and a SNV dataset with eight billion variants to annotate the 125,748 exomes. I do not know how to read PERL, so I can't evaluate the quality of the code, but the repo is well organized, and the README thoroughly describes the functions. Given the requirements (SAMtools, human genome, PhyloCSF) I did not even attempt to install the software locally. I usually run SAMtools on Stampede 2 at the Texas Advanced Computing, so I was going to test it there, but I couldn't install HAIL on Stampede. I also attempted to test Hail and LOFTEE on the Google Cloud Platform Dataproc cluster, but I'm a first-time user, and I wasn't able to overcome installation problems.

For review purposes, it would be ideal if someone with Google Cloud expertise reviewed the software. For training purposes, creating a tutorial (video or blog post) about how to use LOFTEE with Hail on Google would be very valuable to those looking to use these tools for new analyses or to reproduce the analyses described in Karczewski et al.

The Hail website contains extensive documentation on using it in Google cloud, as well as running VEP (which includes LOFTEE by default). The software is updated very frequently, so a blog post or video would likely become out of date rather quickly; however, the Hail documentation is kept up-to-date with the code (e.g. VEP and LOFTEE documentation at https://hail.is/docs/0.2/methods/genetics.html#hail.methods.vep) and its usage on Google cloud (https://hail.is/docs/0.2/hail_on_the_cloud.html).

References

Karczewski, K. J., Gauthier, L. D. & Daly, M. J. Technical artifact drives apparent deviation from Hardy-Weinberg equilibrium at CCR5-Δ32 and other variants in gnomAD. bioRxiv 784157 (2019). doi:10.1101/784157

Reviewer Reports on the Second Revision:

Referees' comments:

Referee #1:

I apologize for my delayed reply here. I've finally had a chance to review the authors' responses to my last round of (additional) comments. They have been addressed very well, and I have no further concerns.

Referee #3:

The authors have addressed all my points satisfactorily, hence I would recommend this manuscript for publication now.

Referee #5:

I have reviewed the response to the reviewers' comments and the revised updated code and software.

I am very impressed and satisfied with the revision. The authors updated the R scripts, and I can confirm that I was able to reproduce all the figures (except for Ext. Data Figs. 8 and 9) as expected. Also, the authors' changes to satisfy other reviewer comments have greatly improved the figures themselves. I agree with the authors' rebuttal that unit tests are not suitable for the software, and I am very pleased that they have provided a docker image with all the data and code. I appreciate the extra effort they put into the revision, and I am confident that the readers of this paper will as well.

As a side note, the pdf that links directly to Ext. Data Fig. 1 is a new and improved figure; however, the merged file with the manuscript and figures has an old version of Ext. Data Fig. 1.
